# Impact of reference design on estimating SARS-CoV-2 lineage abundances from wastewater sequencing data

**DOI:** 10.1093/gigascience/giae051

**Published:** 2024-08-08

**Authors:** Eva Aßmann, Shelesh Agrawal, Laura Orschler, Sindy Böttcher, Susanne Lackner, Martin Hölzer

**Affiliations:** Genome Competence Center (MF1), Robert Koch Institute, Berlin 13353, Germany; Center for Artificial Intelligence in Public Health Research (ZKI-PH), Robert Koch Institute, Berlin 13353, Germany; Chair of Water and Environmental Biotechnology, Institute IWAR, Department of Civil and Environmental Engineering Sciences, Technical University of Darmstadt, Darmstadt 64287, Germany; Chair of Water and Environmental Biotechnology, Institute IWAR, Department of Civil and Environmental Engineering Sciences, Technical University of Darmstadt, Darmstadt 64287, Germany; Gastroenteritis and Hepatitis Pathogens and Enteroviruses, Robert Koch Institute, Berlin 13353, Germany; Chair of Water and Environmental Biotechnology, Institute IWAR, Department of Civil and Environmental Engineering Sciences, Technical University of Darmstadt, Darmstadt 64287, Germany; Genome Competence Center (MF1), Robert Koch Institute, Berlin 13353, Germany

**Keywords:** SARS-CoV-2, wastewater, sewage, abundance estimation, next-generation sequencing, benchmark

## Abstract

**Background:**

Sequencing of severe acute respiratory syndrome coronavirus 2 (SARS-CoV-2) RNA from wastewater samples has emerged as a valuable tool for detecting the presence and relative abundances of SARS-CoV-2 variants in a community. By analyzing the viral genetic material present in wastewater, researchers and public health authorities can gain early insights into the spread of virus lineages and emerging mutations. Constructing reference datasets from known SARS-CoV-2 lineages and their mutation profiles has become state-of-the-art for assigning viral lineages and their relative abundances from wastewater sequencing data. However, selecting reference sequences or mutations directly affects the predictive power.

**Results:**

Here, we show the impact of a *mutation-* and *sequence-based* reference reconstruction for SARS-CoV-2 abundance estimation. We benchmark 3 datasets: (i) synthetic “spike-in”’ mixtures; (ii) German wastewater samples from early 2021, mainly comprising Alpha; and (iii) samples obtained from wastewater at an international airport in Germany from the end of 2021, including first signals of Omicron. The 2 approaches differ in sublineage detection, with the marker *mutation-based* method, in particular, being challenged by the increasing number of mutations and lineages. However, the estimations of both approaches depend on selecting representative references and optimized parameter settings. By performing parameter escalation experiments, we demonstrate the effects of reference size and alternative allele frequency cutoffs for abundance estimation. We show how different parameter settings can lead to different results for our test datasets and illustrate the effects of virus lineage composition of wastewater samples and references.

**Conclusions:**

Our study highlights current computational challenges, focusing on the general reference design, which directly impacts abundance allocations. We illustrate advantages and disadvantages that may be relevant for further developments in the wastewater community and in the context of defining robust quality metrics.

## Background

Coronavirus disease 2019 (COVID-19), the highly contagious viral illness caused by severe acute respiratory syndrome coronavirus 2 (SARS-CoV-2), is the most consequential global health crisis since the era of the influenza pandemic of 1918. Since its discovery, SARS-CoV-2 has caused >775 million confirmed cases of COVID-19 [1], and currently >4,200 SARS-CoV-2 lineages have been defined by the *Pango* network [2–4]. Genome sequencing has played a central role during the COVID-19 pandemic and beyond in supporting public health agencies, monitoring emerging mutations in the SARS-CoV-2 genome, and advancing precision vaccinology and optimizing molecular tests [5–7]. Massive sequencing of clinical samples has made monitoring of emerging virus variants possible while emphasizing temporal and spatial variation. With ongoing transmission, further mutations occur in the genome that are part of the viral evolutionary process and result in unique fingerprints. These fingerprints, along with other metrics such as the number of samples with the same mutation profile and their geographic occurrence, are used to label SARS-CoV-2 variants, such as through the nomenclature system proposed and maintained by the Pangolin network and tool [2, 3]. These definitions of virus variants and lineages and the associated mutation profiles can then be used to search for and estimate the proportion of SARS-CoV-2 lineages in mixed samples (e.g., wastewater).

Sequencing capacity, however, is limited, cannot be sustained over the long term for so many clinical samples, and only allows extrapolation based on a relatively small fraction of all infections occurring during the pandemic. In addition, with decreasing incidence numbers, sampling and sequencing efforts are decreasing, raising the need for representative, medium-scale, and sustainable surveillance systems [7] or other approaches. From 1 January 2020 until 19 April 2023, 931,260 genome sequences of COVID-19–positive clinical samples from Germany have been uploaded to the international GISAID platform [8], representing a proportion of 2.426% out of a total of 38,388,247 reported SARS-CoV-2 cases in Germany [9]. In Germany and other countries, complete detection and sequencing of all positive cases were impossible due to the high infection numbers. However, wastewater-based epidemiology (WBE) has shown the potential to get a much broader snapshot of the SARS-CoV-2 variant circulation at a community level [10–15]. Integrating genome sequencing with WBE can provide information on circulating SARS-CoV-2 variants in a region [16, 17]. The sequencing methods commonly used in WBE are similar to the ones used for clinical samples, using a general strategy that employs the sequencing of the whole genome via amplification of small, specific regions of the SARS-CoV-2 genome (i.e., targeted sequencing of amplicons via predefined primer sequences) [11, 14, 18–20]. Targeted sequencing can achieve a high degree of coverage of informative regions of the genome and, most important, reveal to some extent which polymorphisms are linked, making it possible to track SARS-CoV-2 variants of concern (VOCs) and other virus variants.

A particular challenge in performing sequencing and analysis of SARS-CoV-2 from wastewater samples concerns the viral RNA present in many individual fragments rather than complete viral genomes. In addition, these fragments come from the excretions of many infected individuals, making it challenging, if not impossible, to reconstruct individual genomes using bioinformatic approaches like the ones developed for clinical samples of individual patients. Thus, the degradation and fragmentation of SARS-CoV-2 RNA, combined with the presence of multiple virus variants in wastewater samples, make it challenging to reconstruct reliable, complete consensus genomes, often resulting in sequences that represent either a mixture of lineages or predominantly the most abundant variant. In need of computational approaches to analyze mixed wastewater samples, several groups developed similar tools for quality control, sequencing data analysis, and SARS-CoV-2 lineage abundance estimation instead of reconstructing a single consensus genome [10, 16, 18, 21–31]; see Table 1.

**Table 1:** Collection of tools available for sequencing data analysis in WBE and SARS-CoV-2 lineage proportion estimation. We distinguish the tools roughly based on their approach to define a reference set into those using predefined marker mutations and those relying on full genome sequences or both. The 2 implementations we selected for reference construction and our comparison are indicated in bold. Please note that C-WAP [31] wraps multiple approaches while also including a new *mutation-based* tool, LINDEC.

*Mutation based*		
Tool	Citation	Code
**MAMUSS**	[20]	github.com/lifehashopes/MAMUSS
Freyja	[21]	github.com/andersen-lab/Freyja
Lineagespot	[22]	github.com/nikopech/lineagespot
LCS	[23]	github.com/rvalieris/LCS
Alcov	[24]	github.com/Ellmen/alcov
VaQuERo	[16]	github.com/fabou-uobaf/VaQuERo
MMMVI	[25]	github.com/dorbarker/voc-identify
PiGx	[26]	github.com/BIMSBbioinfo/pigx_sars-cov-2
SAMRefiner	[18]	github.com/degregory/SAM_Refiner
COJAC	[10]	github.com/cbg-ethz/cojac
wastewaterSPAdes	[30]	—
gromstole	—	github.com/PoonLab/gromstole
CovMix	—	github.com/chrisquince/covmix

*Sequence Based*		
Tool	Citation	Code
**VLQ-nf**	this study	https://github.com/rki-mf1/VLQ-nf
VLQ	[29]	github.com/baymlab/wastewater_analysis
VirPool	[27]	github.com/fmfi-compbio/virpool
V-pipe SC2	[28]	cbg-ethz.github.io/V-pipe/sars-cov-2

*Mutation Based And Sequence Based*		
Tool	Citation	Code
C-WAP	[31]	github.com/CFSAN-Biostatistics/C-WAP

Most approaches focus on detecting predefined characteristic marker mutations in the sequenced reads and utilize this information for abundance estimation. Common to all these tools is that they require a reference set of either signature marker mutations (hereafter called *mutation based*) or complete genome sequences (hereafter called *sequence based*) from which characteristic mutation profiles or *k*-mers (short subsequences of length *k*) are derived.

Kayikcioglu et al. [31] compared the performance of 5 selected approaches for SARS-CoV-2 lineage abundance estimation on simulated and publicly available mixed population samples. They found that Kallisto [32], as first suggested by Baaijens et al. [29], followed by Freyja [21], achieved most accurate estimations. Sutcliffe et al. [33] compared 9 computational tools using simulated genomic data in another recent study. Among other things, they tested how the background noise of a previously unknown lineage affects quantification, finding a weak but significant effect on the estimate of the frequency of known lineages that are part of the reference.

In a *mutation-based* approach, to estimate the proportion of specific SARS-CoV-2 variants present in a mixed sample, mutations or combinations of mutations characteristic or unique for these variants based on clinical samples can be compared with the mutations detectable in the sample. In principle, and as implemented in a previously used approach [20] (which we refer to here as MAMUSS, Table 1), the occurrence of mutations can be represented by the value of the relative abundance of a VOC or other viral variant. First, the frequency of occurrence of each mutation is calculated from the multiplication of the reads and the allele frequency. The relative abundance describes the percentage ratio of the sum of the read abundance of the characteristic mutations of a SARS-CoV-2 virus variant and the sum of the read abundance of all mutations found in a sample. Accordingly, only the previously selected virus variants and signature mutations that form the reference set are evaluated, and others that may occur in the sample are ignored. Another prominent *mutation-based* approach is implemented in the tool Freyja [21]. Freyja solves the demixing problem to recover relative lineage abundances from mixed SARS-CoV-2 samples using lineage-determining mutational “barcodes” derived from the UShER global phylogenetic tree [34]. Using mutation abundances and sequencing depth measurements at each position in the genome, Freyja estimates the abundance of lineages in the sample.

As a different methodological approach to reconstruct a reference, the full genome sequence information can be used to automatically select appropriate features (e.g., signature mutations, *k*-mers) and to use them to evaluate the proportions of SARS-CoV-2 variants in wastewater samples instead of a preselected set of marker mutations (*sequence based*) [27–29] (Table 1). Again, information derived from sequencing of clinical samples and their lineage annotation are used to generate a representative reference dataset that can be then searched via established (pseudo)-alignment methods such as Kallisto [32], as suggested by Baaijens et al. in their VQL tool [29].

**Figure 1:**
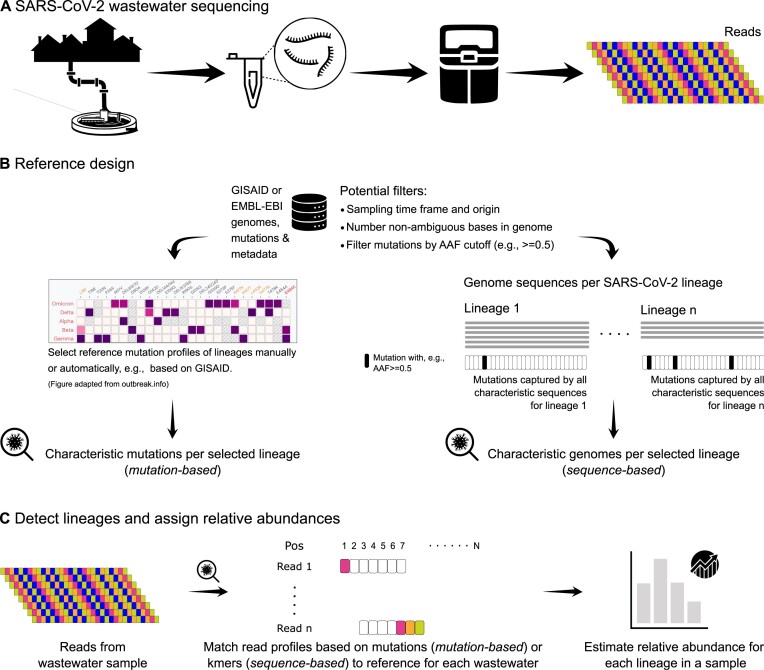
Schematic overview of reference design and lineage abundance estimation from SARS-CoV-2 wastewater sequencing data. (A) Wastewater samples are collected from sewer influent, for example. RNA is extracted and, in the context of SARS-CoV-2, usually amplified as cDNA using established primer schemes and then sequenced to obtain short snippets of viral RNA (*reads*). (B) Current methods (Table 1 for lineage assignment and abundance estimation) need a reference dataset, usually constructed from genomes and mutations derived from clinical sequencing and patient samples. Here, we distinguish 2 general approaches to design the reference, where either marker mutations are preselected (*mutation based*) or full-genome sequences are selected (*sequence based*). (C) The data analysis part may differ considerably depending on the implementation. However, all tools attempt to assign known lineages and estimate their frequency in the mixed sample based on mutations that can be detected in the reads. Our study uses MAMUSS as an exemplary *mutation-based* approach based on a 2-indicator classification and preselected marker mutations characteristic for certain lineages [20]. For the *sequence-based* approach, we use a Nextflow implementation (VLQ-nf) of the slightly adjusted VLQ pipeline as proposed by Baaijens et al. [29] and is based on the tool Kallisto. AAF: alternative allele frequency, used as a cutoff to define a mutation as a feature.

In this study, we specifically investigated the effects of reference design and composition on the assignment of relative abundances of SARS-CoV-2 lineages from wastewater sequencing data. As mentioned, various tools have been developed during the pandemic (Table 1), and they all have different facets in calculating relative abundances [17, 31, 33]. Here, we tested MAMUSS as a *mutation-based* reference representative and VLQ-nf as a *sequence-based* reference representative on 3 datasets: (i) a synthetic scenario of “spike-in” mixture samples; (ii) samples from Germany from a European wastewater study from early 2021, mainly comprising the VOC Alpha [12]; and (iii) a sample obtained from wastewater sequencing at the international airport in Frankfurt am Main, Germany, from the end of 2021, including first signals of the VOC Omicron [20]. The 2 approaches for lineage abundance estimation (*mutation based*/*sequence based*) are mainly distinguished by the input dataset used for the reference set design and subsequent lineage assignment (Fig. 1). Here, we compare exemplary implementations of both general approaches. MAMUSS, as previously applied in [20], implements a representative basic workflow for the *mutation-based* approach focusing on unique marker mutations. For the *sequence-based* approach, we use pseudo-alignments via Kallisto [32] as proposed initially by [29] and their VLQ tool. Based on their idea and scripts, we implemented a slightly modified version of VLQ in a Nextflow [35] pipeline that we call VLQ-nf [36]. We chose VLQ for our *sequence-based* method because it relies on Kallisto as an established tool for quantifying transcripts [32]. A major benefit of implementing the representative methods was the complete control over code, parameters, and inputs, which allowed us to understand better, compare, and interpret the results of our benchmark study and the effects on the reference design. For all 3 datasets, we deliberately selected data from 1 sequencing technology, Ion Torrent, to demonstrate reference design and *mutation*/*sequence-based* effects in a specific, controlled context with which we have much experience [12, 20, 37]. However, it must be noted as a limitation of our study that we are only investigating 1 sequencing technology.

We show that both the *mutation-based* and *sequence-based* approach can reflect the proportions of SARS-CoV-2 lineages in the different samples but also comprise differences in resolution and the detection of similar sublineages depending on the reference set. Both approaches also show advantages and disadvantages in selecting signature marker mutations and genome sequences, respectively. For the *mutation-based* approach as implemented in MAMUSS, it became more and more challenging to select (sub)lineage-defining marker mutations that provide robust assignments in the context of the increasing diversity and convergent evolution of SARS-CoV-2 lineages.

## Data Description

We selected 3 wastewater datasets for our comparison to cover (i) a synthetic scenario of “spike-in” mixture samples (*Standards; n* = 16 samples); (ii) actual wastewater samples from early 2021 from a large European study and collected in Germany [12], mainly comprising the VOC Alpha (*Pan-EU-GER; n* = 7 samples); and (iii) 1 sample from the end of 2021, including first signals of the VOC Omicron obtained from wastewater at the international airport in Frankfurt am Main, Germany (*FFM-Airport; n* = 1 sample) [20]. The *Standards* comprise RNA from 10 SARS-CoV-2 variants (including the original Wuhan-Hu-1 A.1 lineage), which were mixed in different proportions to generate 16 samples for library preparation and sequencing via Ion Torrent (Table 2). The sequencing data for the *Standards* benchmark are available under the NCBI BioProject number PRJNA912560. Please note that no real wastewater was used to construct the *Standards* (see Methods). The Pan-EU WBE study produced high-quality sequencing data for SARS-CoV-2 wastewater samples across 20 European countries, including 54 municipalities, and is available under the NCBI BioProject number PRJNA736964 [12]. We selected the 7 German samples from this study (SRX11122519 and SRX11122521–SRX11122526; *Pan-EU-GER*) for our benchmark, which were sampled in March 2021 and mainly cover the rise of the VOC Alpha during that time. Lastly, we obtained 1 sample (SRR17258654; NCBI BioProject number PRJNA789814) from wastewater sampling in November 2021 at the international airport in Frankfurt am Main (*FFM-Airport*), where we found first signals and low proportions of the VOC Omicron arriving during that time in Germany [20]. Note that we deliberately selected Ion Torrent as sequencing technology to harmonize between the selected datasets and to focus on the reference design and *mutation*/*sequence-based* effects in a specific, controlled context with which we have much experience [12, 20, 37] (see also “Potential implications” section).

**Table 2:** Composition of synthetic mixture “spike-in” *Standards*. Here we show the proportions of which different SARS-CoV-2 lineages were mixed to generate a collection of artificial samples for our benchmark. For example, the sample Mix_01 comprises 25% original Wuhan-Hu-1 A.1 and 75% Alpha B.1.1.7 ($0.25_{org}-0.75_{alpha}$). All samples were sequenced with Ion Torrent and raw data are available under BioProject number PRJNA912560 in the NCBI Sequence Read Archive. Please note that no real wastewater was used to construct these synthetic mixtures because we wanted to reduce any side effects for our *gold standard* in the context of this study.

Sample ID	Composition
Mix_01	$0.25_{org}-0.75_{alpha}$
Mix_02	$0.25_{org}-0.25_{beta}-0.5_{alpha}$
Mix_03	$0.25_{alpha}-0.25_{beta}-0.25_{gamma}-0.25_{org}$
Mix_04	$0.5_{org}-0.5_{iota}$
Mix_05	$0.25_{org}-0.25_{iota}-0.5_{{omiBA2.5}}$
Mix_06	$0.25_{alpha}-0.25_{iota}-0.25_{{omiBA2.5}}-0.25_{omiBA2}$
Mix_07	$0.5_{{omiBA2.5}}-0.5_{omiBA2}$
Mix_08	$0.25_{org}-0.25_{alpha}-0.25_{{omiBA2.5}}-0.25_{omiBA2}$
Mix_09	$0.5_{deltaAY1}-0.5_{deltaAY2}$
Mix_10	$0.25_{deltaAY1}-0.25_{deltaAY2}-0.5_{delta}$
Mix_11	$0.25_{deltaAY1}-0.25_{deltaAY2}-0.5_{omiBA1}$
Mix_12	$0.25_{deltaAY1}-0.25_{deltaAY2}-0.25_{{omiBA1}}-0.25_{{omiBA2.5}}$
Mix_13	$0.25_{deltaAY1}-0.25_{deltaAY2}-0.25_{omiBA1}-0.25_{omiBA2}$
Mix_14	$0.5_{delta}-0.25_{omiBA1}-0.25_{omiBA2}$
Mix_15	$0.25_{deltaAY1}-0.25_{deltaAY2}-0.25_{omiBA1}-0.25_{{omiBA2.5}}$
Mix_16	$0.25_{alpha}-0.25_{delta}-0.25_{omiBA1}-0.25_{omiBA2}$

$_{org}$ – Wuhan-Hu-1 A.1; $_{alpha}$ – Alpha B.1.1.7; $_{beta}$ – Beta B.1.351; $_{gamma}$ – Gamma P.1; $_{iota}$ – Iota B.1.526; $_{delta}$ – Delta B.1.617.2; $_{deltaAY1}$ – Delta AY.1; $_{deltaAY2}$ – Delta AY.2; $_{omiBA1}$ – Omicron BA.1; $_{omiBA2}$ – Omicron BA.2; $_{omiBA2.5}$ – Omicron BA.2.5

## Analyses

### Both the *mutation-based* and *sequence-based* approaches yield similar SARS-CoV-2 lineage proportions for mixed *Standard* samples but differ on sublineage level

We analyzed our *Standards* dataset (Table 2) using the *sequence-based* approach implemented in VLQ-nf and an implementation of a *mutation-based* approach, MAMUSS (Table 1). Given ground-truth knowledge, we assessed the qualitative and quantitative performance of both methods, yielding controlled insights into the strengths and limitations of each approach. When comparing the results with the actual sample composition in the following sections, we define a false-positive hit as a lineage that was estimated with a frequency above zero without being included in the sample mixture. Analogously, we define a false-negative hit as a lineage that was not detected by a tool even though it is included in the sample mixture by design.

**Figure 2:**
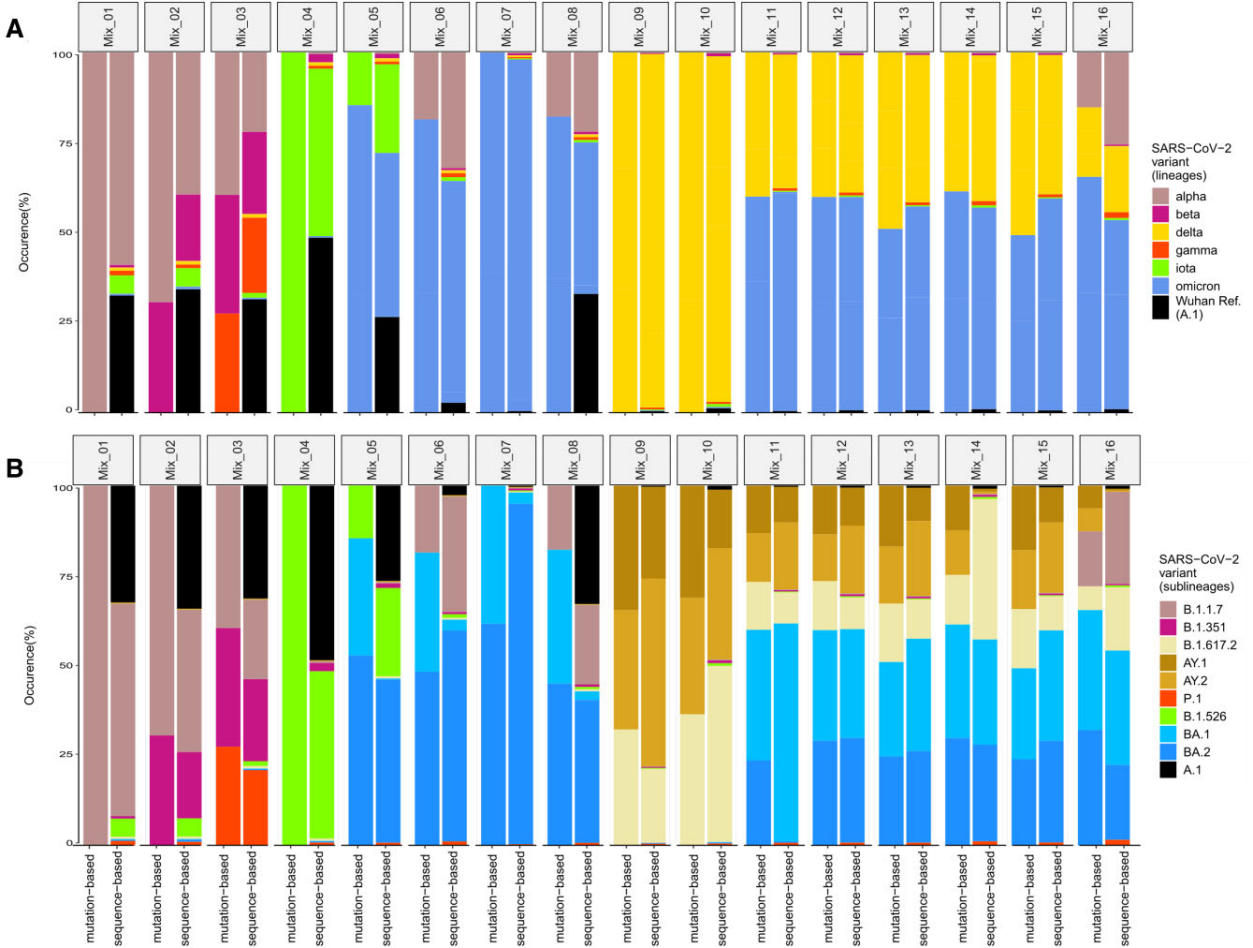
Comparison of the occurrence of predefined mixtures of SARS-CoV-2 variants (*Standards*) (A) at Pangolin parent lineage level and (B) at Pangolin sublineage resolution based on the *sequence-based* (VLQ-nf) and *mutation-based* (MAMUSS) approach.

VLQ-nf detected all correct spike-in lineages across all samples. The output for every sample showed, however, a certain amount of false-positive predictions comprising lineages that are part of our reference set but not used as spike-ins (Fig. 2). We observed the most consistent false-positive estimations for Gamma (P.1), with up to 1.61% abundance across all samples. In contrast, MAMUSS did not detect all spike-in lineages but showed more robust results in quantifying fewer false positives in the samples (Fig. 2).

When comparing false detection and over- or underestimation for both approaches, we partly observed similar patterns among specific groups of lineages: the *mutation-based* approach showed a bias in samples comprising A.1 toward not being able to detect A.1. In sample Mix_06, the *mutation-based* approach could not detect Iota (B.1.526) and falsely detected BA.1. The *sequence-based* approach considerably underestimated B.1.526 in Mix_06, whereas it falsely detected B.1.526 in Mix_01 and Mix_02.

Furthermore, both approaches showed distinct patterns of false estimation among B.1.617.2 (Delta) and its sublineages AY.1 and AY.2. In samples containing no Delta and only Delta sublineages, both approaches falsely detected Delta while underestimating AY.1 or AY.2. In samples containing only Delta and no Delta sublineages, MAMUSS falsely detected AY.1 and AY.2, while underestimating Delta. In samples containing Delta and Delta sublineages, VLQ-nf overestimated Delta and underestimated AY.1, while MAMUSS overestimated Delta sublineages and underestimated Delta.

Both approaches estimated BA.1 and BA.2 without distinct conflicts among each other. We observed slight over- or underestimation in the abundance of Omicron lineages to co-occur with underestimation of Delta sublineages in samples Mix_10–16.

Finally, we found both approaches to match the ground-truth proportions of the *Standards* samples well on the parent lineage level. On the sublineage level, we found the false-negative detection of B.1.526 in sample Mix_06 and the quantification conflicts among Delta (sub)lineages to be the most prominent differences between both approaches. For the *mutation-based* approach, we found the false-negative detection of A.1 to be the second most prominent shortcoming observed in this experiment.

### VLQ-nf detects Alpha sublineages while MAMUSS finds distinctly larger abundances for rising lineages Beta, Gamma, and Delta in the *Pan-EU-GER* data

We analyzed German samples from the Pan-EU study [12] using both approaches to assess their performance on wastewater sequencing data. In the lack of ground-truth knowledge, we evaluated both approaches by relating the lineage predictions and quantification to the pandemic background in Germany based on data from clinical sampling strategies. Moreover, we performed experiments on wastewater sequencing data to evaluate the potential benefits of wastewater-based surveillance compared to clinically based data.

According to global surveillance projects based on clinical genomic sequence data [38–41], the pandemic situation in Europe from February until April 2021 was mainly dominated by the SARS-CoV-2 lineages Alpha, Beta, cases of B.1.177 and sublineages, B.1.258 and sublineages, and B.1.160 (Supplementary Fig. S1). The pandemic situation in Germany at that time was mainly dominated by VOCs Alpha and Beta as well as lineages B.1.177.86, B.1.177.81, B.1.258, B.1.177, and B.1.160. According to GISAID submissions during that time [7], approximately the same lineages and multiple other low-abundant global and European sublineages were reported from clinical sampling strategies. Here we focused the comparison on the lineages Alpha (B.1.1.7), Beta (B.1.351), Gamma (P.1), Delta (B.1.617.2), and the respective sublineages, as those were or became the dominant lineages around the time of wastewater sampling in Germany in the context of the Pan-EU project [12].

**Figure 3:**
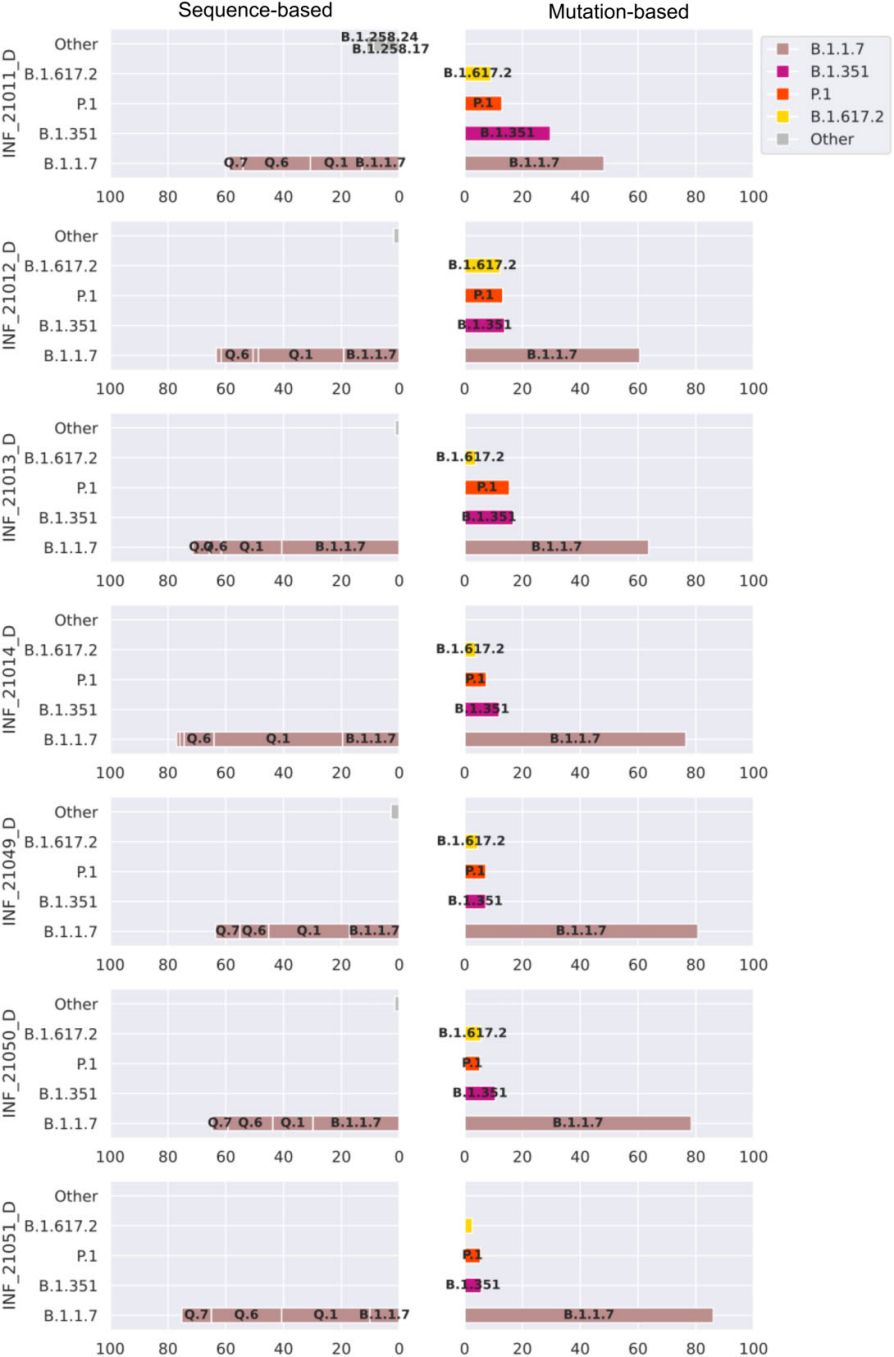
Comparison of the results for the *Pan-EU-GER* analysis using VLQ-nf (*sequence based*, left) versus MAMUSS (*mutation based*, right). Abundance predictions are plotted above a cutoff of 1% abundance and labeled at a threshold of 3% abundance. VLQ-nf detected abundances for B.1.617.2, P.1, and B.1.351 sublineages below 1%, which are not visible at the scale of this figure. The x-axis shows the percentage of predicted lineage abundances for the *Pan-EU-GER* analysis.

With VLQ-nf, we quantified the lineage and sublineage level. In comparison, MAMUSS predicted lineage abundances only at the parent level (Fig. 3). Both approaches predicted Alpha (sub)lineages to be the most abundant lineages in the dataset. Specifically, the *sequence-based* approach found Alpha sublineages Q.1 and Q.7 to be the most abundant. Yet, those Alpha sublineages were not reported among the most frequent cases based on clinical sampling strategies (see Supplementary Fig. S1). However, this is not necessarily the case, as the SARS-CoV-2 lineages can circulate in different proportions in wastewater and clinical environments. We also need to take into account the dynamic nomenclature system. The discrepancy could also be due to the retrospective definition and late classification of Alpha sublineages and again emphasizes the potential influence of reference bias. We also detected Beta, Gamma, and Delta (sub)lineages at abundances below 1%, which are not visible at the scale of Fig. 3. In contrast, we found distinctly larger abundances of Beta, Gamma, and Delta in the samples using MAMUSS.

### *Mutation*- and *sequence-based* approaches recover a similar Omicron proportion from an early airport wastewater sample

We used both approaches to analyze a real wastewater sequencing sample (SRR17258654, *FFM-Airport*) [20]. We compared lineage predictions and quantification against the pandemic background in Europe and South Africa at the time of wastewater sampling. We evaluated both approaches in terms of their ability to detect (sub)lineages at low abundances, specifically to detect low-abundant signals of Omicron BA.1, which was the dominant Omicron sublineage circulating during that time (BA.2 was not yet detected in clinical or wastewater sequencing data).

The pandemic situation in Europe and South Africa from October to December 2021 was dominated by Delta sublineages and increasing incidences of Omicron and its sublineages [38–41] (Supplementary Fig. S2). According to GISAID submissions, mostly Delta sublineages and a few cases of Omicron and other minor global sublineages were reported based on clinical sampling strategies.

**Figure 4:**
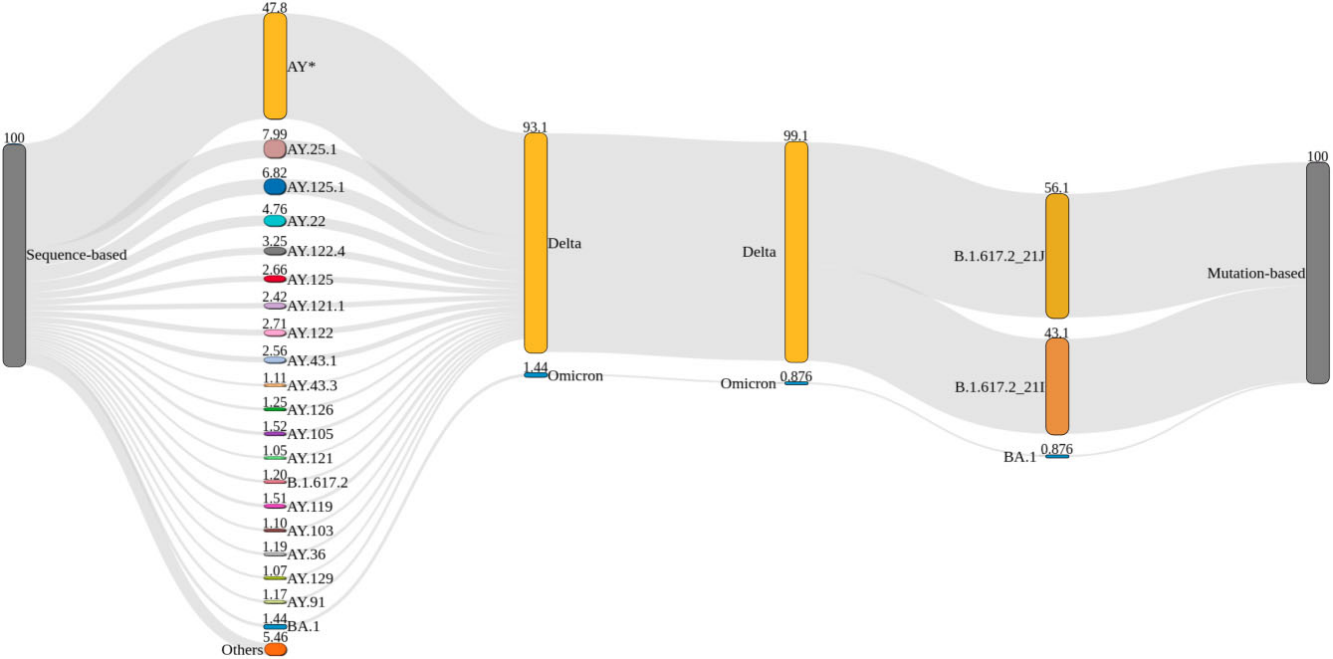
Sankey plot comparing the detected lineage proportions for the *sequence-based* approach (VQL-nf, left) and the *mutation-based* approach (MAMUSS, right) for 1 airport wastewater sample (SRR17258654) [20]. Both approaches detect a similar amount of Delta and Omicron (BA.1) in the sample. At the same time, VQL-nf can achieve a higher sublineage resolution (AY lineages) based on the full genome information in the reconstructed reference index and utilizing pseudo-alignments. MAMUSS can, as configured for this analysis and based on the limited reference set, distinguish between 2 slightly different B.1.617.2 clades as defined by Nextstrain. For the *sequence-based* approach, only lineages with a proportion of at least 1% are shown, and all other AY sublineages are pooled in AY* and all other lineages in “Others.”

With VLQ-nf, we detected many Delta sublineages at abundances ranging from less than 1% to around 8% that in sum contribute over 93% abundance in the wastewater sample (Fig. 4). Roughly half of the detected Delta sublineages were estimated with abundances of less than 1%. In terms of Omicron, VLQ-nf detected BA.1 with 1.44%. Finally, we observed lineages and sublineages from other families with abundances of less than 1% (“Other”).

We observed a similar lineage abundance profile with MAMUSS. We found that most abundance consists of 2 approximately equally abundant Delta sublineages. We detected a small proportion close to 1% of Omicron. Compared to VLQ-nf, we did not find any low-abundant quantification for other (sub)lineages, explained by the smaller reference dataset only composed of a particular collection of marker mutations.

We found that the estimated abundance profiles of lineages from both approaches matched well with the pandemic background in Europe and South Africa at the time of wastewater sampling. However, when considering abundance estimations of the *sequence-based* approach at the sublineage level, we discovered differences regarding the most abundantly predicted Delta sublineages compared to the more prominent Delta sublineages derived from clinical sampling strategies in European and South African GISAID submissions (compare Fig. 4 and Supplementary Fig. S2). The *sequence-based* approach predicted AY.25.1, AY.125.1, AY.122.4, AY.121, and AY.43.1 to be most abundant in the analyzed sample. In contrast, GISAID submissions showed AY.4, AY.43, AY.122, AY.4.2, AY.126, AY.4.2.2, and AY.98 as the most frequent Delta sublineages in Europe during that time. Additionally, we found AY.45, AY.32, AY.91, AY.116, AY.122, AY.6, and AY.46 to be the highest reported Delta sublineages in South Africa. While our predictions do not match the clinically reported frequencies, some of our predictions belong to the same lineage family as the most frequently reported lineages from clinical sampling (e.g., AY.43.1 is a sublineage of AY.43, AY.122.4 is a sublineage of AY.122, and AY.125.1 is a sublineage of AY.125, which we found among the 20 most frequently reported lineages in Europe using VLQ-nf).

### Alternative allele frequency and size of reference database impact the *sequence-based* method, but the effects also depend on lineage composition in the sample

To better understand the impact of specific parameters on the performance of the *sequence-based* method, we performed parameter escalation experiments (see Methods) on the *Standards* benchmark set as well as the *Pan-EU-GER* and *FFM-Airport* datasets. Due to the similar findings for all 3 datasets, here we only present the results based on the *Standards* and refer to the results of the *Pan-EU-GER* and *FFM-Airport* datasets in the supplementary material (subsection “Alternative allele frequency and size of reference database impact the *sequence-based* method but the effects are dependent on sample composition”). We investigated the impact of reference construction parameters on lineage proportion estimation and aimed at uncovering the potential bias of the pseudo-alignment implemented in the *sequence-based* method. Specifically, we focused on the alternative allele frequency (AAF) threshold and the maximum number of sequences per lineage. The AAF threshold defines the minimum alternative allele frequency for a mutation to be considered characteristic of a lineage. First, genome sequences are added as lineage references so that each mutation that exceeds the AAF threshold is detected at least once by as few sequences as possible. Next, additional genomes are added until the maximum number of sequences per lineage is reached. Thus, the AAF threshold controls the level of genomic variation captured for each lineage, and the maximum number of sequences per lineage controls the reference size.

#### Standards

**Figure 5:**
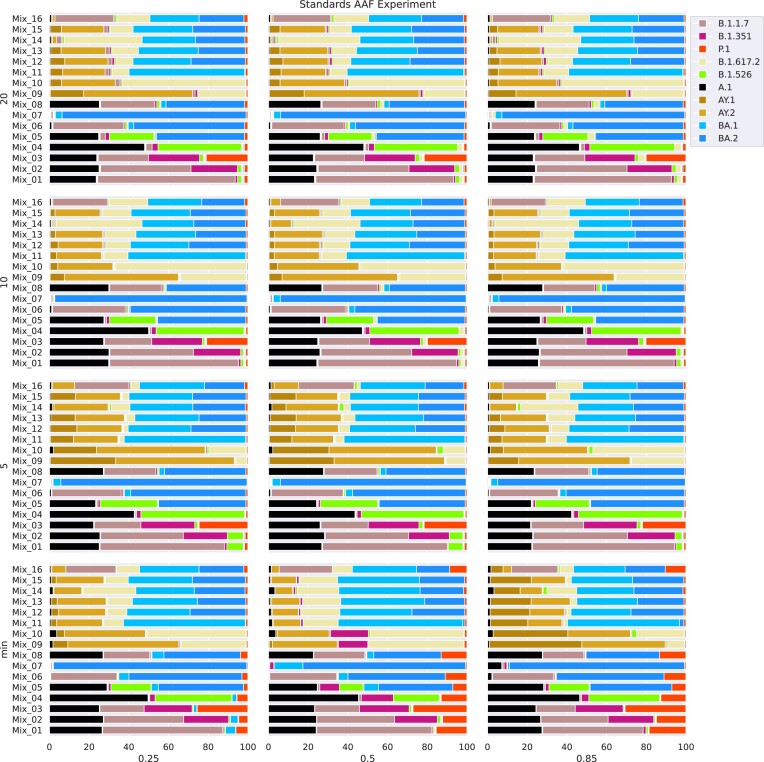
Results for the parameter escalation experiments on the *Standards* samples using the *sequence-based* method (VLQ-nf). We analyzed the *Standards* with different parameterizations for reference construction (x-axis: increasing AAF threshold, y-axis: increasing maximum number of sequences per lineage). VLQ-nf, using pseudo-alignments, detected all lineages and estimated abundance profiles well across most samples and parameter settings. However, we also observed prominent detection ambiguities among Delta and its sublineages and found consistently low-abundant false positives for specific groups of lineages. Continuously increasing or decreasing parameter settings caused heterogeneous changes in the estimated abundance proportions across samples. The *sequence-based* method showed to perform better when using a reference set larger than the minimum reference size. Still, we found noise levels to increase distinctly when using the maximum reference size among the considered settings.

Across most *Standards* samples and experiments, VLQ-nf detected all spike-in lineages and predicted reasonable estimates (Fig. 5). However, we consistently observed low-abundant false-positive hits in all of our mixed samples, comprising lineages that are part of the reference index but not used as spike-ins. We found the most prominent false-positive detection to be Gamma. We observed similar patterns of false-positive detection and false estimation among specific groups of lineages across all parameter settings: for the first 8 samples Mix_01 to Mix_08, most cases of false estimation of spike-in lineage abundances occurred alongside false positives or negatives of B.1.526 and false positives of BA.1. For the samples Mix_09 to Mix_16, we observed most detection conflicts to involve ambiguities among Delta and its sublineages AY.1 and AY.2.

We found that the detection and quantification performance of the *sequence-based* method via VLQ-nf changed with varying thresholds for the alternative allele frequency and maximum number of genomes per reference lineage. Specifically, we found those changes to vary across samples and observed them not to behave identically with consistent parameter changes. For example, at the minimum reference size (Supplementary Table S1), we observed abundance predictions for samples Mix_09 and Mix_11–16 to first improve with an increasing AAF threshold. However, with a further increasing AAF threshold, we observed more false estimations of Delta sublineages. Furthermore, although Mix_10 shares most of its spike-in lineages with Mix_09, the performance of abundance estimations for sample Mix_10 first decreased and then improved again when increasing the AAF threshold.

We made a similar observation for the maximum number of sequences per lineage. With an AAF threshold of 0.5, the abundance estimates for Mix_01 improved with increasing number of reference genomes per lineage, while we found them to deteriorate for Mix_09, which includes a distinctly different sample composition. Overall, we found lineage abundance estimations to become slightly more robust across varying AAF thresholds with increasing reference size. This is best reflected in the abundance profiles for samples Mix_09 to Mix_15 when looking at the proportional changes across increasing AAF settings for the minimum reference size throughout the reference with 10 sequences per lineage.

Finally, we found that the AAF threshold and the reference size affect the performance of the *sequence-based* method. Although we did not observe a clear and consistent pattern of impact, we found that the effects of varying parameter settings may depend on the sample composition. Specifically, we observed the strongest impact of parameter changes for samples containing lineages with a higher degree of shared genomic similarity. Also, we found the AAF threshold to affect estimates slightly more than the reference size. We detected similar results for the *Pan-EU-GER* and *FFM-Airport* datasets. We provide details for these 2 datasets in the supplementary material (see Supplementary Figs. S3 and S4).

#### Final choice of parameters for benchmark reference construction

Within the scope of the parameter escalation experiments described here, we wanted to determine parameters with a good prediction performance without manipulating the benchmark in favor of the *sequence-based* approach (VQL-nf). Finally, based on our parameter testing and the 3 different datasets, we chose an AAF threshold of 0.25 and a reference size of at most 5 sequences per lineage. This threshold allowed us to limit the size of the reference dataset and still allows reasonable detection and quantification results across all 3 benchmark datasets while keeping computational resources moderate.

## Discussion

It is apparent that the composition of the reference used must have a large impact on the determination of relative SARS-CoV-2 abundances in wastewater sequence data. Especially given the dynamic and constantly updated SARS-CoV-2 lineage definitions [3], the reference genome sequences and the signature mutations derived from them also change frequently. Of course, the various tools (Table 1) and their parameters developed for estimating the relative abundance of lineages from wastewater sequencing data also have an impact. Here, however, we have specifically focused on the effects of the reference design.

We selected 2 general approaches to design reference datasets and estimate SARS-CoV-2 lineage proportions from wastewater sequencing samples (Fig. 1). On the one hand, selected marker mutations that are characteristic for certain SARS-CoV-2 lineages can be used for annotation and lineage proportion estimation (*mutation based*, MAMUSS). Here, the read sequences derived from a wastewater sample are mapped against a reference genome from which differences (mutations) are detected and compared against the selected marker mutations. On the other hand, full SARS-CoV-2 genome sequences can be used to create a reference index without prior collection of specific mutations (*sequence based*, VLQ-nf). Here, the problem of selecting appropriate marker mutations is shifted to selecting representative lineages from which features for the classification task are derived. An exemplary implementation of this approach based on the pseudo-aligner Kallisto [32] was recently proposed by Baaijens et al. [29]. Based on their work, we developed a Nextflow pipeline for higher automation and reproducibility and detecting SARS-CoV-2 lineage proportions from wastewater data using pseudo-alignments (VLQ-nf). In this approach, a selection of whole-genome SARS-CoV-2 sequences (target reference set) and the reads (query) are composed into *k*-mers, which are then efficiently compared to quantify lineage abundances, similar to quantifying gene expression in an RNA sequencing study.

To benchmark reference designs from both methods (*mutation based* via MAMUSS, *sequence based* via VLQ-nf), we selected 3 test scenarios: (i) a spike-in experiment with different SARS-CoV-2 lineage mixes, (ii) samples obtained for Germany from a Pan-EU wastewater study, and (iii) a wastewater sample from a German airport during the time when Omicron emerged.

In general, both approaches detected SARS-CoV-2 lineage abundances from our test cases. The most remarkable difference was in the number of detected sublineages, which also directly correlates with the reference design. VLQ-nf generally detected a larger diversity of sublineages in comparison to MAMUSS, which can be explained by the underlying reference indices. It became increasingly difficult to select a representative set of marker mutations for the *mutation-based* approach and the implementation we used as more and more (sub)lineages were defined and there was overlap in mutations (convergent evolution). In contrast, the *sequence-based* approach as suggested by Baaijens et al. [29] can build a reference index on a large collection of SARS-CoV-2 full genome sequences derived from clinical samples and thus, potentially, better reflect diversity on sublineage levels. However, we also observed a certain amount of *noise* in the pseudo-alignment results, causing potential false-positive hits in our test datasets. Other approaches, like Freyja [21], partly tackle this problem by deriving signature mutation profiles automatically, for example, using the whole phylogenetic diversity of current SARS-CoV-2 sequences reflected in an UShER tree [34]. However, here we have also observed that the inclusion of a large diversity in the reference can lead to distributed abundance assignments between closely related (sub)lineages, reducing the true relative abundance of a lineage (Supplementary Figs. S5 and S6). Of course, the impact can be reduced by limiting lineage coverage to a specific time period, but this, in turn, can also affect frequency assignments.

In more detail, both approaches performed similarly in detecting and estimating spike-in lineage abundances for the *Standards* dataset (Fig. 2). The predictions are more similar on the parent-lineage level compared to the sublineage level. If their estimations differ, this can be mostly attributed to differences in the mutations/lineages included in the respective reference data: for both approaches, the final predictions heavily depend on the construction of the reference dataset. In addition, both approaches had difficulties differentiating closely related sublineages correctly.

For the *Pan-EU-GER* dataset, both approaches reflect well the pandemic background in Germany during the time of sampling, but we detected some limitations and potential sources for bias: the choice of marker mutations and reference lineages impacts the level of detection (i.e. lineage- vs. sublineage-level estimations) but also the amount of low-abundance detection. Potentially, everything that is defined in the reference dataset can also be detected, which might lead to an increased number of false-positive predictions. The whole-genome sequences or mutations used to create the reference index impact the degree of ambiguity and, thus, (low-abundant) false-positive detection. This may explain why both approaches predicted distinctly different abundances on the parent-lineage level compared to the other 2 benchmark experiments. Therefore, we think that especially the *sequence-based* approach requires the definition of a false-positive threshold to differentiate between low-abundant false-positive hits and low-abundant true positives.

Both approaches also detected low-frequency lineages for the *FFM-Airport* dataset. Again, the *sequence-based* approach detects a distinctly higher amount of low-abundant lineages, also reflecting the higher diversity of the reference index.

We performed an additional parameter benchmark to identify important key parameters impacting the *sequence-based* pseudo-alignment approach using VLQ-nf. One parameter that strongly affects the results is the AAF cutoff. In connection with the reference size (the number of genomes), we observed different effects of changing the AAF. Our experiments also showed that the effect of the same parameter changes (increasing or decreasing AAF) does not yield consistent results among the different datasets. The degree of lineage ambiguity depends on the considered composition of lineages and sublineages. The effect of included/excluded mutations due to adjusted AAF parameter settings is variable, as different mutations have different effects in differentiating lineages. The effect of those parameter changes is most notable among more similar lineages. We also observed that with a larger reference size, the effect of the AAF parameter becomes smaller and overall abundance estimations improve. One explanation might be that by adding further reference genomes for a lineage, low-frequency mutations are implicitly introduced and increase the genomic variation that is represented by the reference dataset. These additional low-frequency mutations might support the differentiation of certain (sub)lineages better and thus slightly improve abundance estimations.

## Potential Implications

In this study, we focus exclusively on Ion Torrent sequencing data to specifically investigate the influence of reference database composition and analysis parameters on lineage abundance estimates in wastewater sequencing. While acknowledging that incorporating data from additional platforms like PacBio, Nanopore, and Illumina could broaden the analysis of variability and robustness, we chose Ion Torrent due to its established efficacy in achieving high horizontal genome coverage in our sequencing runs [12, 20, 37], critical for assessing the impact of reference bias. This focused approach allows us to explore the considerable effects that reference selection and analytical settings have on lineage abundance results, a crucial area for accurate viral surveillance. Future studies might explore a comparative analysis across different platforms to enhance understanding of lineage composition and abundance estimation in wastewater samples. However, our current study is intentionally limited to specific research objectives related to reference bias in a *mutation-based* and *sequence-based* setting and in the context of declining clinical sequencing and the dilution of available reference sequences.

Further, we only selected 2 exemplary implementations of the *mutation-* and *sequence-based* approaches MAMUSS and VLQ-nf, respectively, out of an increasing number of scripts, tools, and pipelines becoming available for computational SARS-CoV-2 lineage estimation from wastewater sequencing (Table 1) [10, 16, 18, 21–31]. Thus, our benchmark results also reflect and are limited by the individual characteristics of these 2 implementations. However, we focused on these 2 approaches to investigate the impact of reference design using implementations where we could easily control parameters and input—similar to the decision for the Ion Torrent technology. Currently, a comprehensive benchmark comparison for the existing SARS-CoV-2 wastewater analysis tools is lacking. The developers of Freyja compared a selection of tools on a spike-in mixed sample [21] where they found that Freyja outperformed VLQ [29] in accuracy at higher expected proportions and observed noticeably longer computation times for both VLQ and LCS [23]. To counteract the effect on lineage abundance detection, some methods filter the mutations considered for lineage assignment based on sequencing depth [16] or adjust their mathematical model for differences in depth and coverage and expected error rates [21, 27]. Similarly, the PiGx tool addresses the limitations of estimating lineages at low abundances by weighting specific signature mutations for lineages that are expected to occur at low frequencies [26]. Another recent study compared 9 computational tools but only used simulated genomic data [33]. As a next step, a broader evaluation of all available tools for analyzing SARS-CoV-2 wastewater sequencing data is urgently needed to guide usage and further development [42].

## Conclusion

Academic researchers have pioneered wastewater monitoring of SARS-CoV-2 and overcome several technical and methodological challenges [15]. Thanks to these efforts, wastewater-based pathogen surveillance has rapidly become a valuable public health tool for detecting SARS-CoV-2 that can excellently complement syndromic surveillance or other monitoring tools. However, public health authorities are now faced with the task of integrating these achievements into robust and continuous public health surveillance systems that can be operated and expanded over the long term. Performance parameters must be defined and communicated to the public health authorities to include wastewater-based pathogen surveillance data. In this context, continuous updating of reference datasets, in the context of retrospective analyses or time series, is essential to ensure comparability between time points. For example, genomic sequences of newly defined lineages might already be present in wastewater samples from previous weeks. However, bioinformatic analysis of previous samples could not detect the novel lineage because it was not included in the reference dataset at that time point. Continuously updated reference datasets can support comparing and interpreting wastewater sequencing time-series data. Yet, harmonizing the reference used would require recalculating older abundance estimates, which may conflict with the standard reporting requirements of public health authorities. However, this problem is not specific to wastewater-based SARS-CoV-2 sequencing data but also applies to genomics sequencing of patient samples. One solution might be to not only focus on lineages but also report mutations that are not affected by any nomenclature scheme and are not subject to delayed definitions. On the other hand, it is undeniable that lineages played a crucial role in communication during the COVID-19 pandemic. Recently, McBroome et al. [43] proposed a novel framework for a more automated and scalable designation of viral pathogen lineages from (clinical) genomic data.

Wastewater sequencing data also offer the potential to uncover *cryptic* (novel, undescribed) lineages, although resolving the full genomic profile of those solely from wastewater data still poses several challenges [11, 21]. In this context, approaches utilizing artificial intelligence might present a promising next step for the improved detection of cryptic SARS-CoV-2 lineages from wastewater sequencing data and increasing trends, although, right now, not much in use [44]. However, first studies appear that use machine learning for the early detection of new signals from wastewater data and the description of potential new SARS-CoV-2 lineages [45, 46]. Finally, the lessons learned from the sequencing efforts and implementations for SARS-CoV-2 detection from wastewater sequencing data can and should be adapted to other pathogens to further advance wastewater genomic surveillance efforts.

## Methods

### Benchmark dataset 1: *Standards*

We procured synthetic SARS-CoV-2 RNA samples (Twist Biosciences), which were used to prepare 16 different mixtures (Table 2) containing different SARS-CoV-2 variants. From the pooled RNA, cDNA was synthesized using SuperScript VILO Master Mix (Thermofisher Scientific), followed by library preparation using the Ion AmpliSeq SARS-CoV-2 Research Panel (Thermofisher Scientific) according to the manufacturer’s instructions. This panel consists of 237 primer pairs, resulting in an amplicon length range of 125–275 bp, which cover the near-full genome of SARS-CoV-2. We performed 2 sequencing runs to achieve at least 1 million mapped reads per sample. For each sequencing run, 8 libraries were multiplexed and sequenced using an Ion Torrent 530 chip on an Ion S5 sequencer (Thermofisher Scientific) according to the manufacturer’s instructions. The raw sequence data were uploaded to the NCBI Sequence Read Archive under BioProject number PRJNA912560.

### Data processing: *mutation-based* reference design and lineage proportion estimation via MAMUSS

We used the SARS-CoV-2 Research Plug-in Package, which we installed in our Ion Torrent Suite software (v5.12.2) of Ion S5 sequence. We used the SARS_CoV_2_coverageAnalysis (v5.16) plugin [47], which maps the generated reads to a SARS-CoV-2 reference genome (Wuhan-Hu-1-NC_045512/MN908947.3), using TMAP software included in the Torrent Suite. The summary of mapping of each sample mentioned in Table 2 is provided in Supplementary Table S2. For mutation calls, additional Ion Torrent plugins were used as described previously [37] and detailed below. First, all single nucleotide variants were called using Variant Caller (v5.12.0.4) with “Generic - S5/S5XL (510/520/530) - Somatic - Low Stringency” default parameters. Then, for annotation and determination of the base substitution effect, we used COVID19AnnotateSnpEff (v1.3.0.2), a plugin developed explicitly for SARS-CoV-2 and based on the original SnpEff [48]. To construct reference marker mutation sets for MAMUSS, we used data from GISAID [8]. For each SARS-CoV-2 variant, we downloaded the variant surveillance database and selected complete clinical genome sequences, followed by counting the prevalence of its associated mutations. The 50 most prevalent mutations associated with each variant were used as a reference marker mutation set. The lineage abundance estimation is based on the read depth and allele frequency of each mutation detected in a wastewater sample followed by a 2-indicator classification and comparison to the preselected marker mutations characteristic for certain lineages. For further details, see the MAMUSS GitHub repository [49].

### Data processing: *sequence-based* reference design and lineage proportion estimation via VLQ-nf

Instead of relying only on manually or algorithmically selected marker mutations, another computational approach utilizes, in a first step, full genome information. For example, Baaijens et al. [29] presented a method to estimate the abundance of variants in wastewater samples based on well-established computational techniques initially used for RNA sequencing quantification. Here, the main idea is that quantifying different transcripts derived from the same gene is computationally similar to the abundance estimation of different SARS-CoV-2 lineages derived from the same parental genome. Via Kallisto [32], they perform pseudo-alignments of the raw reads against an index of preselected and downsampled full genome SARS-CoV-2 sequences with respective lineage information. Therefore, their approach may be less influenced by the preselection of mutations based on clinical relevance, frequency, or other parameters that mostly drive *mutation-based* tools and thus may be better suited for sublineage discrimination. The approach comprises 2 steps: (i) selecting reference genome sequences for index construction and (ii) pseudo-alignment of the reads and lineage abundance estimation. First, a reference dataset of SARS-CoV-2 genome sequences must be selected. For that, we use data from GISAID [8] and filter for human–host sequences, N-count information, pangolin annotation [2, 3], origin (country, continent), and sampling date. These metadata are used to preselect sequences based on geographic origin (continent, country), a sampling time frame, and the number of N bases. Next, the pipeline performs a variant calling against a reference sequence (per default index Wuhan-Hu-1, NC_045512.2) and subsequently samples sequences to select characteristic mutation profiles for each input lineage. Within a lineage, sequences are sampled based on an alternative allele frequency cutoff (e.g., AAF >0.5) so that each mutation is represented at least once until an upper limit of sequences per lineage is reached. From this downsampled and representative set of full genome sequences, a Kallisto index is constructed. Now, the raw reads from a FASTQ file are pseudo-aligned against this index and lineage abundances are quantified. This is done by estimating for each read the probability of originating from each genome sequence in the reference using expectation maximization and finally aggregating the resulting probabilities across the lineage labels associated with every reference genome.

For our comparative study, we used the initial idea and code base from Baaijens et al. [29, 50] and implemented a Nextflow pipeline [35, 36] with the purpose of automating the steps and making our analyses fully reproducible. In this context, we discovered some issues in the pipeline version 61dd29df* of Baaijens et al. and implemented minor adjustments. This includes updating data-processing scripts according to the most recent GISAID data format and allowing the sequence selection based on alternate allele frequencies to consider multiallelic sites. Meanwhile, the authors have addressed those issues with similar code changes in their current pipeline version. In pipeline version 61dd29df*, sequences are selected for the reference index if they carry an AAF filter passing mutation that is not yet covered until the reference set for the respective lineage meets the maximum allowed number of sequences. We wanted to provide the possibility for using a minimum reference setup to reduce data storage requirements and allow exploring the impact of different AAF thresholds on abundance estimation. Subsequently, we adjusted the AAF filter to first sample a minimum set of genome sequences so that all passing mutations are included at least once, before increasing the reference set to the number of maximum sequences per lineage. We ran our pipeline version v1.0.0 for all analyses in this benchmark study.

### Reconstruction of indices for the *sequence-based* approach

The *sequence-based* (VLQ-nf) approach highly depends on the selection and reconstruction of the reference dataset for the Kallisto index. Thus, we reconstructed different indices for our 3 benchmark datasets to mimic the pandemic situation during the time of sampling. We used GISAID data for all indices and extracted subsets based on metadata filters.

For the benchmark of the 16 mixed *Standards*, we constructed a reference dataset comprising the included SARS-CoV-2 lineages. We selected a time frame of 2 weeks around the peak of global incidences[38, 39] for each lineage included in the mix (Table 3). We only kept records with at least 29,500 nonambiguous bases. Because we also included the original Wuhan-Hu-1 reference sequence in mixed samples Mix_01 to Mix_05 and Mix_08, we first excluded all A.1 sequences from the preselected set. Then, we selected reference sequences with characteristic mutation profiles for all lineages except A.1 as described before, allowing a maximum number of 5 sequences per lineage. Then, we added the sampled A.1 sequences again to the final reference set, as otherwise the A.1 sequences would have been excluded by the pipeline because they do not show any AAF in comparison to the Wuhan-Hu-1 reference. On average, we selected 5 sequences for a lineage to capture every mutation against the wild-type with an AAF >0.25 (within-lineage variation) and a maximum of 5 allowed sequences per lineage.

**Table 3:** For each lineage in the *Standards* dataset, we selected the time frame where infection numbers peaked globally [38]. Based on the listed time frames, we sampled genome sequences from GISAID for reference reconstruction. We downloaded the GISAID records on 2 March 2022.

Lineage	Time frame
A.1	2020-03-01:2020-03-14
B.1.1.7	2021-05-01:2021-05-14
B.1.351	2021-01-20:2021-02-02
P.1	2021-04-20:2021-05-03
B.1.526	2021-03-20:2021-04-02
BA.2	2022-02-01:2022-02-14
BA.1	2021-12-01:2021-12-14
B.1.617.2	2021-06-25:2021-07-08
AY.1	2021-08-01:2021-08-14
AY.2	2021-06-25:2021-07-08

For the *Pan-EU-GER* samples (collected between 10 and 30 March 2021), we reconstructed the reference from clinical GISAID records we downloaded on 27 January 2022. We selected only European sequences sampled between 1 February 2021 and 30 April 2021, with at least 29,500 nonambiguous bases. To reflect the influx of variants from other European countries, we have not only selected sequences from Germany. On average, we then selected 3 sequences per lineage to capture every mutation against the wild-type with an AAF >0.25 (within-lineage variation) and allowing at most 5 reference sequences per lineage.

For the *FFM-Airport* dataset, we reconstructed the reference from GISAID records we downloaded on 11 February 2022. We selected genome sequences from European and South African clinical records sampled between 1 October 2021 and 31 December 2021, again with at least 29,500 nonambiguous bases. On average, 4 sequences were selected for a lineage to capture every mutation against the wild-type with an AAF >0.25 (within-lineage variation). Again, we allowed at most 5 sequences to be included per lineage.

### Lineage abundance estimation with the *sequence-based* approach

After reconstructing different reference indices for our benchmark datasets, we used specific Kallisto commands implemented in a Nextflow pipeline to prepare Kallisto mapping indices, compute pseudo-alignments of each benchmark dataset against its reference index, and estimate lineage abundances following the original idea and code of Baaijens et al. [29].

First, we built a Kallisto index from the reference database (default *k*-mer = 31). Next, for each sample in a benchmark dataset, we pseudo-aligned all reads against the corresponding Kallisto index and estimated the abundance of each reference sequence in the sample. We quantified our benchmark datasets in single-read mode with an average fragment length of 200 nt with a standard deviation of 20 nt. Finally, a customized script grouped the estimated abundances by the lineage annotation of the respective sequences and summed them up into a final lineage abundance estimation for the analyzed sample. For the *Pan-EU-GER* and *FFM-Airport* datasets, we further summarized the estimated abundances by the country information of the analyzed samples to compare the pseudo-alignment and *mutation-based* approach on the country level.

### Assessing parameter impact and potential bias with the pseudo-alignment approach

We performed parameter escalation experiments with our 3 benchmark datasets using the *sequence-based* method (VLQ-nf) to assess the impact of the AAF threshold and the cutoff for a maximum number of sequences per lineage on lineage abundance estimation. More importantly, we used the resulting observations to inform our choice of parameters used for the final benchmarking against the *mutation-based* method (MAMUSS). In this context, we aimed to determine a setting with a good prediction performance and reasonable computational effort without manipulating the benchmark in favor of the *sequence-based* method. For every benchmark dataset, we constructed reference indices over a range of 12 possible parameter combinations. For the AAF threshold, we iterated over [0.25, 0.5, 0.85] to cover lower, medium, and high threshold values to define the characteristic mutation profiles. For the maximum number of sequences per lineage, we built the reference index using the minimal sequence sets possible, 5, 10, and 20 sequences per lineage. After lineage abundance estimation with each reference index on the *Standards* dataset, we evaluated prediction performance based on the ground-truth lineage abundances. For the *FFM-Airport* and *Pan-EU-GER* data, we assessed prediction performance by comparing estimated lineage abundances with the pandemic background at the respective time and location.

### Reproducibility of the pseudo-alignment approach

Our Nextflow pipeline of the pseudo-alignment approach [36] generates the reference database in the format of a CSV file containing the metadata information of the final Kallisto index and a FASTA file containing the corresponding sequence data. In the current version v1.0.0, the reference CSV and FASTA can be exactly replicated using the same input data resource and index reconstruction parameters, which leads to slightly different results at every analysis run. The reference CSV is not reproducible due to misplaced random sampling seeds and a missing record sorting strategy in the AAF-based sequence filtering step during reference reconstruction. However, lineage detection and quantification are deterministic given VLQ-nf takes fixed reference datasets as input (final CSV and FASTA reference or already built Kallisto index).

## Availability of Source Code and Requirements

Here, we provide the specifications of our Nextflow implementation (VLQ-nf) of the *sequence-based* approach originally presented by Baaijens et al. [29] and the code for the *mutation-based* approach, MAMUSS.

- Project name: VLQ-nf
- Project homepage: https://github.com/rki-mf1/VLQ-nf
- Operating system(s): Linux, Mac, Windows via Linux subshell
- Programming language: Nextflow
- Other requirements: Conda
- License: GPL-3.0

- Project name: MAMUSS
- Project homepage: https://github.com/lifehashopes/MAMUSS
- Operating system(s): Linux, Mac
- Programming language: R
- Other requirements: R packages are listed in the repository
- License: CC0 1.0 Universal

## Supplementary Material

giae051_GIGA-D-23-00161_Original_Submission

giae051_GIGA-D-23-00161_Revision_1

giae051_Response_to_Reviewer_Comments_Original_Submission

giae051_Reviewer_1_Report_Original_Submission Irene Bassano -- 9/20/2023 Reviewed

giae051_Reviewer_2_Report_Original_Submission Liuyang Zhao -- 1/15/2024 Reviewed

giae051_Reviewer_2_Report_Revision_1 Liuyang Zhao -- 5/9/2024 Reviewed

giae051_Supplemental_Files

## Data Availability

The datasets supporting the results of this article are available in the Open Science Framework repository [51]. All supporting data and materials are available in the *GigaScience* database, GigaDB [52].

## Additional Files

**Supplementary Fig. S1**. Top: The pandemic background across Europe between 1 February and 30 April 2021 was built with Nextstrain.org. Bottom: The outbreak.org variant report for Germany displaying the SARS-CoV-2 lineage prevalence from February to March 2021 based on GISAID sequence data. The most dominant lineages in the plot from bottom to top: light blue = B.1.1.7, light green = B.1, purple = B.1.177.86, light gray = other, blue = B.1.258, dark gray = B.1.221, yellow = B.1.177, orange = B.1.160, light brown = B.1.177.81.

**Supplementary Fig. S2**. Top: The pandemic background across Europe between 1 October and 31 December 2021 was built with Nextstrain.org. Bottom: The outbreak.org variant report for South Africa displaying the SARS-CoV-2 lineage prevalence from October to December 2021 based on GISAID sequence data. The most dominant lineages comprise sublineages of Delta and Omicron but also B.1.351 (light green) and C.2 (light orange).

**Supplementary Fig. S3**. Results for the parameter escalation experiments on the *Pan-EU-GER* samples using the *sequence-based* method using pseudo-alignment implementation. We analyzed the dataset with different parameterization for reference construction (x-axis: increasing AAF threshold, y-axis: increasing maximum number of sequences per lineage). Abundance predictions are displayed at a minimum threshold of 1% and labeled at a threshold of 3%. When comparing with the pandemic background at the time of wastewater sampling, we observed the AAF threshold and the maximum number of sequences per lineage to impact the abundance proportions among Alpha and Q.1 the most. With more sequences per lineage in the reference, we found the impact of the AAF filter on the observed ambiguities to decrease. We found more low-abundant sublineages predicted in the real wastewater data compared to the *Standards* dataset and found those low-abundant predictions to mostly not change distinctly across varying parameterization.

**Supplementary Fig. S4**. Results for the parameter escalation experiments on the *FFM-Airport* dataset using the *sequence-based* method. We analyzed the dataset with different parameterization for reference construction (x-axis: increasing AAF threshold, y-axis: increasing maximum number of sequences per lineage). Top: Abundance predictions are displayed at a minimum threshold of 1% abundance and labeled at a threshold of 3% abundance. When comparing with the pandemic background at the time of wastewater sampling, we observed the following: overall, we found more sublineages predicted with abundance below 1% compared with the *Standards* dataset and the *Pan-EU-GER* set. The *sequence-based* method detected more low-abundant sublineages with increasing reference size and slightly less low-abundant sublineages with increasing AAF threshold. Both the AAF threshold and the reference size showed an impact on lineage ambiguities among Delta sublineages. Bottom: All abundance predictions are displayed as grouped by their parent lineage. We did not find the abundance predictions for parent lineages to change distinctly across experiments.

**Supplementary Fig. S5**. SARS-CoV-2 lineage abundance assignments via Freyja [21] (v1.3.12) for the *Standards*. We used the full reference UShER set as provided as a default by the tool. In this case, multiple sublineages were predicted and frequencies were distributed among them, resulting in a reduced frequency estimate for the true (parental) lineage and an increase in low-frequency detections. For example, in Mix_07, the sublineages BA.2.16 and BA.2.4 were predicted with almost 50%, respectively, while the included lineage BA.2 was not assigned (compare Supplementary Fig. S6).

**Supplementary Fig. S6**. SARS-CoV-2 lineage abundance assignments via Freyja [21] (v1.3.12) for the *Standards*. We reduced the reference UShER set to the lineages part of our artificial mixtures, instead of using the full UShER barcode dataset as shown in Supplementary Fig. S5.

**Supplementary Table S1**. The minimum reference sizes across the different AAF thresholds considered in the parameter escalation experiments across our 3 benchmark datasets. Here, we list the minimum number of genome sequences required per lineage to capture every mutation with an AAF above the considered AAF threshold at least once based on the implemented sampling strategy during reference construction. The *Standards* reference database required the largest number of sequences to capture the predefined genomic variation. Overall, we observed that with an increasing AAF threshold, the minimum reference sizes per lineage decreased across all 3 benchmark datasets.

**Supplementary Table S2**. The mapping of each *Standards* sample.

## Abbreviations

AAF: alternative allele frequency; COVID-19: coronavirus disease 2019; *FFM-Airport*: 1 sample from the end of 2021, including first signals of the VOC Omicron obtained from wastewater at the international airport in Frankfurt am Main, Germany [20]; MAMUSS: *mutation-based* approach for SARS-CoV-2 lineage abundance estimation; NCBI: National Center for Biotechnology Information; *Pan-EU-GER*: 7 samples from early 2021 from a large European study and collected in Germany, mainly comprising the VOC Alpha [12]; SARS-CoV-2: severe acute respiratory syndrome coronavirus 2; *Standards*: synthetic scenario of 16 “spike-in” mixture SARS-CoV-2 samples; VLQ-nf: *sequence-based* approach for SARS-CoV-2 lineage abundance estimation, inspired by the original VLQ [29]; VOC: variant of concern; WBE: wastewater-based epidemiology.
