# Supplementary material for: Impact of reference design on estimating SARS-CoV-2 lineage abundances from wastewater sequencing data
Source: Gigascience. 2024 Aug 8;13:giae051. doi: 10.1093/gigascience/giae051 (PMC11308188; doi:10.1093/gigascience/giae051)
Supplement: giae051_GIGA-D-23-00161_Revision_1 [file giae051_giga-d-23-00161_revision_1.pdf]

GigaScience

Impact of reference design on estimating SARS-CoV-2 lineage abundances from wastewater sequencing data

--Manuscript Draft--

Manuscript Number:	GIGA-D-23-00161R1
Full Title:	Impact of reference design on estimating SARS-CoV-2 lineage abundances from wastewater sequencing data
Article Type:	Research
Funding Information:	
Abstract:	Background Sequencing of SARS-CoV-2 RNA from wastewater samples has emerged as a valuable tool for detecting the presence and relative abundances of SARS-CoV-2 variants in a community. By analyzing the viral genetic material present in wastewater, researchers and public health authorities can gain early insights into the spread of virus lineages and emerging mutations. Constructing reference datasets from known SARS-CoV-2 lineages and their mutation profiles has become state-of-the-art for assigning viral lineages and their relative abundances from wastewater sequencing data. However, selecting reference sequences or mutations directly affect the predictive power. Results Here, we show the impact of a mutation- and sequence-based reference reconstruction for SARS-CoV-2 abundance estimation. We benchmark three data sets: 1) synthetic “spike-in” mixtures, 2) German wastewater samples from early 2021, mainly comprising Alpha, and 3) samples obtained from wastewater at an international airport in Germany from the end of 2021, including first signals of Omicron. The two approaches differ in sub-lineage detection, with the marker-mutation-based method, in particular, being challenged by the increasing number of mutations and lineages. However, the estimations of both approaches depend on selecting representative references and optimized parameter settings. By performing parameter escalation experiments, we demonstrate the effects of reference size and alternative allele frequency cutoffs for abundance estimation. We show how different parameter settings can lead to different results for our test data sets, and illustrate the effects of virus lineage composition of wastewater samples and references. Conclusions Our study highlights current computational challenges, focusing on the general reference design, which directly impacts abundance allocations. We illustrate advantages and disadvantages that may be relevant for further developments in the wastewater community and in the context of defining robust quality metrics.
Corresponding Author:	Martin Hölzer<br>RKI: Robert Koch Institut<br>Berlin, GERMANY
Corresponding Author Secondary Information:	
Corresponding Author's Institution:	RKI: Robert Koch Institut
Corresponding Author's Secondary Institution:	
First Author:	Eva Aßmann
First Author Secondary Information:	
Order of Authors:	Eva Aßmann
	Shelesh Agrawal
	Laura Orschler
	Sindy Böttcher
	Susanne Lackner
	Martin Hölzer

Powered by Editorial Manager® and ProduXion Manager® from Aries Systems Corporation

**Order of Authors Secondary Information:**	
**Response to Reviewers:**	Dear Dr. Zhou, Dear reviewers,<br><br>Thank you again for handling our manuscript titled “Impact of reference design on estimating SARS-CoV-2 lineage abundances from wastewater sequencing data" (GIGA-D-23-00161).<br><br>We appreciate the constructive comments of the two reviewers and are pleased to attach the revised version of the manuscript along with our detailed responses to the reviewers' comments. We attached our detailed response letter as an additional PDF. Please let us know if that did not work.<br><br>We look forward to the possibility of our study being published in GigaScience and believe it will make a valuable contribution to the ongoing efforts in understanding and utilizing wastewater sequencing data for public health surveillance.<br><br>Thank you for considering our revised manuscript. We are eager to see it contribute to the scientific community and help advance our understanding of SARS-CoV-2 dynamics in wastewater-based epidemiology.<br><br>Best,<br><br>Martin Hölzer<br>(on behalf of all co-authors)
**Additional Information:**	
**Question**	**Response**
Are you submitting this manuscript to a special series or article collection?	No
**Experimental design and statistics**<br><br>Full details of the experimental design and statistical methods used should be given in the Methods section, as detailed in our Minimum Standards Reporting Checklist. Information essential to interpreting the data presented should be made available in the figure legends.<br><br>Have you included all the information requested in your manuscript?	Yes
**Resources**<br><br>A description of all resources used, including antibodies, cell lines, animals and software tools, with enough information to allow them to be uniquely identified, should be included in the Methods section. Authors are strongly encouraged to cite Research Resource Identifiers (RRIDs) for antibodies, model	Yes

Powered by Editorial Manager® and ProduXion Manager® from Aries Systems Corporation

organisms and tools, where possible.<br><br>Have you included the information requested as detailed in our Minimum Standards Reporting Checklist?	
**Availability of data and materials**<br><br>All datasets and code on which the conclusions of the paper rely must be either included in your submission or deposited in publicly available repositories (where available and ethically appropriate), referencing such data using a unique identifier in the references and in the "Availability of Data and Materials" section of your manuscript.<br><br>Have you have met the above requirement as detailed in our Minimum Standards Reporting Checklist?	Yes

Powered by Editorial Manager® and ProduXion Manager® from Aries Systems Corporation

maintex

Click here to access/download;LaTeX - Main Document (TeX file);main.tex

```
This is pdfTeX, Version 3.141592653-2.6-1.40.25 (TeX Live 2023)
(preloaded format=pdflatex 2024.3.8)  5 JUL 2024 05:16
entering extended mode
 restricted \write18 enabled.
 %&-line parsing enabled.
**main.tex
(./main.tex
LaTeX2e <2023-11-01> patch level 1
L3 programming layer <2024-02-20>
(./oup-contemporary.cls
Document Class: oup-contemporary 2017/06/28, v1.1
(c:/texlive/2023/texmf-dist/tex/latex/base/article.cls
Document Class: article 2023/05/17 v1.4n Standard LaTeX document class
(c:/texlive/2023/texmf-dist/tex/latex/base/size10.clo
File: size10.clo 2023/05/17 v1.4n Standard LaTeX file (size option)
)
\c@part=\count188
\c@section=\count189
\c@subsection=\count190
\c@subsubsection=\count191
\c@paragraph=\count192
\c@subparagraph=\count193
\c@figure=\count194
\c@table=\count195
\abovecaptionskip=\skip48
\belowcaptionskip=\skip49
\bibindent=\dimen140
) (c:/texlive/2023/texmf-dist/tex/latex/base/inputenc.sty
Package: inputenc 2021/02/14 v1.3d Input encoding file
\inpenc@prehook=\toks17
\inpenc@posthook=\toks18
) (c:/texlive/2023/texmf-dist/tex/latex/base/fontenc.sty
Package: fontenc 2021/04/29 v2.0v Standard LaTeX package
) (c:/texlive/2023/texmf-dist/tex/generic/iftex/ifpdf.sty
Package: ifpdf 2019/10/25 v3.4 ifpdf legacy package. Use iftex instead.
(c:/texlive/2023/texmf-dist/tex/generic/iftex/iftex.sty
Package: iftex 2022/02/03 v1.0f TeX engine tests
)) (c:/texlive/2023/texmf-dist/tex/latex/microtype/microtype.sty
Package: microtype 2023/03/13 v3.1a Micro-typographical refinements (RS)
(c:/texlive/2023/texmf-dist/tex/latex/graphics/keyval.sty
Package: keyval 2022/05/29 v1.15 key=value parser (DPC)
\KV@toks@=\toks19
) (c:/texlive/2023/texmf-dist/tex/latex/etoolbox/etoolbox.sty
Package: etoolbox 2020/10/05 v2.5k e-TeX tools for LaTeX (JAW)
\etb@tempcnta=\count196
)
\MT@toks=\toks20
\MT@tempbox=\box51
\MT@count=\count197
LaTeX Info: Redefining \noprotrusionifhmode on input line 1059.
LaTeX Info: Redefining \leftprotrusion on input line 1060.
\MT@prot@toks=\toks21
LaTeX Info: Redefining \rightprotrusion on input line 1078.
LaTeX Info: Redefining \textls on input line 1368.
```

```
\MT@outer@kern=\dimen141
LaTeX Info: Redefining \textmicrotypecontext on input line 1988.
\MT@listname@count=\count198
(c:/texlive/2023/texmf-dist/tex/latex/microtype/microtype-pdftex.def
File: microtype-pdftex.def 2023/03/13 v3.1a Definitions specific to
pdftex (RS)

LaTeX Info: Redefining \lsstyle on input line 902.
LaTeX Info: Redefining \lslig on input line 902.
\MT@outer@space=\skip50
)
Package microtype Info: Loading configuration file microtype.cfg.
(c:/texlive/2023/texmf-dist/tex/latex/microtype/microtype.cfg
File: microtype.cfg 2023/03/13 v3.1a microtype main configuration file
(RS)
)) (c:/texlive/2023/texmf-dist/tex/latex/euler/euler.sty
Package: euler 1995/03/05 v2.5
Package: `euler' v2.5 <1995/03/05> (FJ and FMi)
LaTeX Font Info:    Redeclaring symbol font `letters' on input line 35.
LaTeX Font Info:    Encoding `OML' has changed to `U' for symbol font
(Font)              `letters' in the math version `normal' on input line
35.
LaTeX Font Info:    Overwriting symbol font `letters' in version `normal'
(Font)                  OML/cmm/m/it --> U/eur/m/n on input line 35.
LaTeX Font Info:    Encoding `OML' has changed to `U' for symbol font
(Font)              `letters' in the math version `bold' on input line
35.
LaTeX Font Info:    Overwriting symbol font `letters' in version `bold'
(Font)                  OML/cmm/b/it --> U/eur/m/n on input line 35.
LaTeX Font Info:    Overwriting symbol font `letters' in version `bold'
(Font)                  U/eur/m/n --> U/eur/b/n on input line 36.
LaTeX Font Info:    Redeclaring math symbol \Gamma on input line 47.
LaTeX Font Info:    Redeclaring math symbol \Delta on input line 48.
LaTeX Font Info:    Redeclaring math symbol \Theta on input line 49.
LaTeX Font Info:    Redeclaring math symbol \Lambda on input line 50.
LaTeX Font Info:    Redeclaring math symbol \Xi on input line 51.
LaTeX Font Info:    Redeclaring math symbol \Pi on input line 52.
LaTeX Font Info:    Redeclaring math symbol \Sigma on input line 53.
LaTeX Font Info:    Redeclaring math symbol \Upsilon on input line 54.
LaTeX Font Info:    Redeclaring math symbol \Phi on input line 55.
LaTeX Font Info:    Redeclaring math symbol \Psi on input line 56.
LaTeX Font Info:    Redeclaring math symbol \Omega on input line 57.
\symEulerFraktur=\mathgroup4
LaTeX Font Info:    Overwriting symbol font `EulerFraktur' in version
`bold'
(Font)                  U/euf/m/n --> U/euf/b/n on input line 63.
LaTeX Info: Redefining \oldstylenums on input line 85.
\symEulerScript=\mathgroup5
LaTeX Font Info:    Overwriting symbol font `EulerScript' in version
`bold'
(Font)                  U/eus/m/n --> U/eus/b/n on input line 93.
LaTeX Font Info:    Redeclaring math symbol \aleph on input line 97.
LaTeX Font Info:    Redeclaring math symbol \Re on input line 98.
LaTeX Font Info:    Redeclaring math symbol \Im on input line 99.
```

LaTeX Font Info: Redeclaring math delimiter \vert on input line 101.
LaTeX Font Info: Redeclaring math delimiter \backslash on input line
103.
LaTeX Font Info: Redeclaring math symbol \neg on input line 106.
LaTeX Font Info: Redeclaring math symbol \wedge on input line 108.
LaTeX Font Info: Redeclaring math symbol \vee on input line 110.
LaTeX Font Info: Redeclaring math symbol \setminus on input line 112.
LaTeX Font Info: Redeclaring math symbol \sim on input line 113.
LaTeX Font Info: Redeclaring math symbol \mid on input line 114.
LaTeX Font Info: Redeclaring math delimiter \arrowvert on input line
116.
LaTeX Font Info: Redeclaring math symbol \mathsection on input line
117.
\symEulerExtension=\mathgroup6
LaTeX Font Info: Redeclaring math symbol \coprod on input line 125.
LaTeX Font Info: Redeclaring math symbol \prod on input line 125.
LaTeX Font Info: Redeclaring math symbol \sum on input line 125.
LaTeX Font Info: Redeclaring math symbol \intop on input line 130.
LaTeX Font Info: Redeclaring math symbol \ointop on input line 131.
LaTeX Font Info: Redeclaring math symbol \braceld on input line 132.
LaTeX Font Info: Redeclaring math symbol \bracerd on input line 133.
LaTeX Font Info: Redeclaring math symbol \bracelu on input line 134.
LaTeX Font Info: Redeclaring math symbol \braceru on input line 135.
LaTeX Font Info: Redeclaring math symbol \infty on input line 136.
LaTeX Font Info: Redeclaring math symbol \nearrow on input line 153.
LaTeX Font Info: Redeclaring math symbol \searrow on input line 154.
LaTeX Font Info: Redeclaring math symbol \nwarrow on input line 155.
LaTeX Font Info: Redeclaring math symbol \swarrow on input line 156.
LaTeX Font Info: Redeclaring math symbol \Leftrightarrow on input line
157.
LaTeX Font Info: Redeclaring math symbol \Leftarrow on input line 158.
LaTeX Font Info: Redeclaring math symbol \Rightarrow on input line
159.
LaTeX Font Info: Redeclaring math symbol \leftrightarrow on input line
160.
LaTeX Font Info: Redeclaring math symbol \leftarrow on input line 161.
LaTeX Font Info: Redeclaring math symbol \rightarrow on input line
163.
LaTeX Font Info: Redeclaring math delimiter \uparrow on input line
166.
LaTeX Font Info: Redeclaring math delimiter \downarrow on input line
168.
LaTeX Font Info: Redeclaring math delimiter \updownarrow on input line
170.
LaTeX Font Info: Redeclaring math delimiter \Uparrow on input line
172.
LaTeX Font Info: Redeclaring math delimiter \Downarrow on input line
174.
LaTeX Font Info: Redeclaring math delimiter \Updownarrow on input line
176.
LaTeX Font Info: Redeclaring math symbol \leftharpoonup on input line
177.
LaTeX Font Info: Redeclaring math symbol \leftharpoondown on input
line 178.

```
LaTeX Font Info:    Redeclaring math symbol \rightharpoonup on input line
179.
LaTeX Font Info:    Redeclaring math symbol \rightharpoondown on input
line 180
.
LaTeX Font Info:    Redeclaring math delimiter \lbrace on input line 182.
LaTeX Font Info:    Redeclaring math delimiter \rbrace on input line 184.
\symcmmigroup=\mathgroup7
LaTeX Font Info:    Overwriting symbol font `cmmigroup' in version `bold'
(Font)                  OML/cmm/m/it --> OML/cmm/b/it on input line 200.
LaTeX Font Info:    Redeclaring math accent \vec on input line 201.
LaTeX Font Info:    Redeclaring math symbol \triangleleft on input line
202.
LaTeX Font Info:    Redeclaring math symbol \triangleright on input line
203.
LaTeX Font Info:    Redeclaring math symbol \star on input line 204.
LaTeX Font Info:    Redeclaring math symbol \lhook on input line 205.
LaTeX Font Info:    Redeclaring math symbol \rhook on input line 206.
LaTeX Font Info:    Redeclaring math symbol \flat on input line 207.
LaTeX Font Info:    Redeclaring math symbol \natural on input line 208.
LaTeX Font Info:    Redeclaring math symbol \sharp on input line 209.
LaTeX Font Info:    Redeclaring math symbol \smile on input line 210.
LaTeX Font Info:    Redeclaring math symbol \frown on input line 211.
LaTeX Font Info:    Redeclaring math accent \grave on input line 245.
LaTeX Font Info:    Redeclaring math accent \acute on input line 246.
LaTeX Font Info:    Redeclaring math accent \tilde on input line 247.
LaTeX Font Info:    Redeclaring math accent \ddot on input line 248.
LaTeX Font Info:    Redeclaring math accent \check on input line 249.
LaTeX Font Info:    Redeclaring math accent \breve on input line 250.
LaTeX Font Info:    Redeclaring math accent \bar on input line 251.
LaTeX Font Info:    Redeclaring math accent \dot on input line 252.
LaTeX Font Info:    Redeclaring math accent \hat on input line 254.
) (c:/texlive/2023/texmf-dist/tex/latex/merriweather/merriweather.sty
Package: merriweather 2022/09/20 (Bob Tennent) Supports
Merriweather(Sans) font
s for all LaTeX engines.
(c:/texlive/2023/texmf-dist/tex/generic/iftex/ifxetex.sty
Package: ifxetex 2019/10/25 v0.7 ifxetex legacy package. Use iftex
instead.
) (c:/texlive/2023/texmf-dist/tex/generic/iftex/ifluatex.sty
Package: ifluatex 2019/10/25 v1.5 ifluatex legacy package. Use iftex
instead.
) (c:/texlive/2023/texmf-dist/tex/latex/base/textcomp.sty
Package: textcomp 2020/02/02 v2.0n Standard LaTeX package
) (c:/texlive/2023/texmf-dist/tex/latex/xkeyval/xkeyval.sty
Package: xkeyval 2022/06/16 v2.9 package option processing (HA)
(c:/texlive/2023/texmf-dist/tex/generic/xkeyval/xkeyval.tex
(c:/texlive/2023/te
xmf-dist/tex/generic/xkeyval/xkvutils.tex
\XKV@toks=\toks22
\XKV@tempa@toks=\toks23
)
\XKV@depth=\count199
```

```
File: xkeyval.tex 2014/12/03 v2.7a key=value parser (HA)
)) (c:/texlive/2023/texmf-dist/tex/latex/base/fontenc.sty
Package: fontenc 2021/04/29 v2.0v Standard LaTeX package
) (c:/texlive/2023/texmf-dist/tex/latex/fontaxes/fontaxes.sty
Package: fontaxes 2020/07/21 v1.0e Font selection axes
LaTeX Info: Redefining \upshape on input line 29.
LaTeX Info: Redefining \itshape on input line 31.
LaTeX Info: Redefining \slshape on input line 33.
LaTeX Info: Redefining \swshape on input line 35.
LaTeX Info: Redefining \scshape on input line 37.
LaTeX Info: Redefining \sscshape on input line 39.
LaTeX Info: Redefining \ulcshape on input line 41.
LaTeX Info: Redefining \textsw on input line 47.
LaTeX Info: Redefining \textssc on input line 48.
LaTeX Info: Redefining \textulc on input line 49.
)) (c:/texlive/2023/texmf-dist/tex/latex/mathastext/mathastext.sty
Package: mathastext 2023/12/29 v1.3zb Use the text font in math mode
(JFB)

Package mathastext Info: Starting the math mode configuration.
\mst@exists@muskip=\muskip16
\mst@forall@muskip=\muskip17
\mst@prime@muskip=\muskip18
\mst@do@nonletters=\toks24
\mst@do@easynonletters=\toks25
\mst@do@az=\toks26
\mst@do@AZ=\toks27
\symmtoperatorfont=\mathgroup8
\symmtletterfont=\mathgroup9
(   mathastext:   ) ! and ?
(   mathastext:   ) punctuation: , . : ; and \colon
LaTeX Info: Redefining \relbar on input line 894.
LaTeX Info: Redefining \rightarrowfill on input line 897.
LaTeX Info: Redefining \leftarrowfill on input line 902.
(   mathastext:   ) + and =
LaTeX Info: Redefining \Relbar on input line 993.
(   mathastext:   ) adding = ; and + to \nfss@catcodes
(   mathastext:   ) parentheses ( ) [ ] and slash /
(   mathastext:   ) alldelims: < > \backslash \setminus | \vert \mid \{
\}
LaTeX Font Info:    Redeclaring math delimiter \backslash on input line
1039.
LaTeX Font Info:    Redeclaring math symbol \setminus on input line 1051.
LaTeX Info: Redefining \models on input line 1060.
(   mathastext:   ) \# \mathdollar \% \&
(   mathastext:   ) \imath and \jmath
LaTeX Font Info:    Overwriting math alphabet `\Mathnormalbold' in
version `nor
mal'
(Font)                  T1/Merriwthr-OsF/b/it --> T1/Merriwthr-OsF/b/it
on inpu
t line 2516.
LaTeX Font Info:    Overwriting math alphabet `\Mathnormalbold' in
version `bol
```

```
d'
(Font)              T1/Merriwthr-OsF/b/it --> T1/Merriwthr-OsF/b/it
on inpu
t line 2516.
LaTeX Font Info:    Overwriting symbol font `mtletterfont' in version
`normal'
(Font)              T1/Merriwthr-OsF/m/it --> T1/Merriwthr-OsF/m/it
on inpu
t line 2516.
LaTeX Font Info:    Overwriting symbol font `mtletterfont' in version
`bold'
(Font)              T1/Merriwthr-OsF/m/it --> T1/Merriwthr-OsF/b/it
on inpu
t line 2516.
LaTeX Font Info:    Overwriting symbol font `mtoperatorfont' in version
`normal
'
(Font)              T1/Merriwthr-OsF/m/n --> T1/Merriwthr-OsF/m/n on
input
line 2516.
LaTeX Font Info:    Overwriting symbol font `mtoperatorfont' in version
`bold'
(Font)              T1/Merriwthr-OsF/m/n --> T1/Merriwthr-OsF/b/n on
input
line 2516.
LaTeX Font Info:    Overwriting math alphabet `\Mathbf' in version
`normal'
(Font)              T1/Merriwthr-OsF/b/n --> T1/Merriwthr-OsF/b/n on
input
line 2516.
LaTeX Font Info:    Overwriting math alphabet `\Mathbf' in version `bold'
(Font)              T1/Merriwthr-OsF/b/n --> T1/Merriwthr-OsF/b/n on
input
line 2516.
LaTeX Font Info:    Overwriting math alphabet `\Mathit' in version
`normal'
(Font)              T1/Merriwthr-OsF/m/it --> T1/Merriwthr-OsF/m/it
on inpu
t line 2516.
LaTeX Font Info:    Overwriting math alphabet `\Mathit' in version `bold'
(Font)              T1/Merriwthr-OsF/m/it --> T1/Merriwthr-OsF/b/it
on inpu
t line 2516.
LaTeX Font Info:    Overwriting math alphabet `\Mathsf' in version
`normal'
(Font)              T1/MerriwthrSans-OsF/m/n --> T1/MerriwthrSans-
OsF/m/n o
n input line 2516.
LaTeX Font Info:    Overwriting math alphabet `\Mathsf' in version `bold'
(Font)              T1/MerriwthrSans-OsF/m/n --> T1/MerriwthrSans-
OsF/b/n o
n input line 2516.
LaTeX Font Info:    Overwriting math alphabet `\Mathtt' in version
`normal'
```

```
(Font)                  T1/lmtt/m/n --> T1/lmtt/m/n on input line 2516.
LaTeX Font Info:    Overwriting math alphabet `\Mathtt' in version `bold'
(Font)                  T1/lmtt/m/n --> T1/lmtt/b/n on input line 2516.
(   mathastext:   ) Latin letters in the `normal', resp. `bold',
(   mathastext:   ) math versions are now set up to use the fonts
(   mathastext:   ) T1/Merriwthr-OsF/m/it, resp. T1/Merriwthr-OsF/b/it.
(   mathastext:   ) Other characters (digits, ...) and \log-like names
will be
(   mathastext:   ) typeset with the n shape.
(   mathastext:   ) \hbar
(   mathastext:   ) minus as endash
(   mathastext:   ) The italic option is in effect.
(   mathastext:   ) \HUGE has been (re)-defined.
(   mathastext:   ) mathastext has declared larger sizes for subscripts.
(   mathastext:   ) To keep LaTeX defaults, use option
`defaultmathsizes'.

Package mathastext Info: Loading is complete.  You can now use
\Mathastext to
(mathastext)             modify the normal and bold math versions.  Use
it
(mathastext)             with optional argument or use \MTDeclareVersion
to
(mathastext)             declare additional math versions.
) (c:/texlive/2023/texmf-dist/tex/latex/relsize/relsize.sty
Package: relsize 2013/03/29 ver 4.1
) (c:/texlive/2023/texmf-dist/tex/latex/ragged2e/ragged2e.sty
Package: ragged2e 2023/06/22 v3.6 ragged2e Package
\CenteringLeftskip=\skip51
\RaggedLeftLeftskip=\skip52
\RaggedRightLeftskip=\skip53
\CenteringRightskip=\skip54
\RaggedLeftRightskip=\skip55
\RaggedRightRightskip=\skip56
\CenteringParfillskip=\skip57
\RaggedLeftParfillskip=\skip58
\RaggedRightParfillskip=\skip59
\JustifyingParfillskip=\skip60
\CenteringParindent=\skip61
\RaggedLeftParindent=\skip62
\RaggedRightParindent=\skip63
\JustifyingParindent=\skip64
) (c:/texlive/2023/texmf-dist/tex/latex/xcolor/xcolor.sty
Package: xcolor 2023/11/15 v3.01 LaTeX color extensions (UK)
(c:/texlive/2023/texmf-dist/tex/latex/graphics-cfg/color.cfg
File: color.cfg 2016/01/02 v1.6 sample color configuration
)
Package xcolor Info: Driver file: pdftex.def on input line 274.
(c:/texlive/2023/texmf-dist/tex/latex/graphics-def/pdftex.def
File: pdftex.def 2022/09/22 v1.2b Graphics/color driver for pdftex
) (c:/texlive/2023/texmf-dist/tex/latex/graphics/mathcolor.ltx)
Package xcolor Info: Model `cmy' substituted by `cmy0' on input line
1350.
Package xcolor Info: Model `hsb' substituted by `rgb' on input line 1354.
```

```
Package xcolor Info: Model `RGB' extended on input line 1366.
Package xcolor Info: Model `HTML' substituted by `rgb' on input line
1368.
Package xcolor Info: Model `Hsb' substituted by `hsb' on input line 1369.
Package xcolor Info: Model `tHsb' substituted by `hsb' on input line
1370.
Package xcolor Info: Model `HSB' substituted by `hsb' on input line 1371.
Package xcolor Info: Model `Gray' substituted by `gray' on input line
1372.
Package xcolor Info: Model `wave' substituted by `hsb' on input line
1373.
) (c:/texlive/2023/texmf-dist/tex/latex/colortbl/colortbl.sty
Package: colortbl 2024/02/20 v1.0g Color table columns (DPC)
(c:/texlive/2023/texmf-dist/tex/latex/tools/array.sty
Package: array 2023/10/16 v2.5g Tabular extension package (FMi)
\col@sep=\dimen142
\ar@mcellbox=\box52
\extrarowheight=\dimen143
\NC@list=\toks28
\extratabsurround=\skip65
\backup@length=\skip66
\ar@cellbox=\box53
)
\everycr=\toks29
\minrowclearance=\skip67
\rownum=\count266
) (c:/texlive/2023/texmf-dist/tex/latex/graphics/graphicx.sty
Package: graphicx 2021/09/16 v1.2d Enhanced LaTeX Graphics (DPC,SPQR)
(c:/texlive/2023/texmf-dist/tex/latex/graphics/graphics.sty
Package: graphics 2022/03/10 v1.4e Standard LaTeX Graphics (DPC,SPQR)
(c:/texlive/2023/texmf-dist/tex/latex/graphics/trig.sty
Package: trig 2021/08/11 v1.11 sin cos tan (DPC)
) (c:/texlive/2023/texmf-dist/tex/latex/graphics-cfg/graphics.cfg
File: graphics.cfg 2016/06/04 v1.11 sample graphics configuration
)
Package graphics Info: Driver file: pdftex.def on input line 107.
)
\Gin@req@height=\dimen144
\Gin@req@width=\dimen145
) (c:/texlive/2023/texmf-dist/tex/latex/xpatch/xpatch.sty
(c:/texlive/2023/texm
f-dist/tex/latex/l3kernel/expl3.sty
Package: expl3 2024-02-20 L3 programming layer (loader)
(c:/texlive/2023/texmf-dist/tex/latex/l3backend/l3backend-pdftex.def
File: l3backend-pdftex.def 2024-02-20 L3 backend support: PDF output
(pdfTeX)
\l__color_backend_stack_int=\count267
\l__pdf_internal_box=\box54
))
Package: xpatch 2020/03/25 v0.3a Extending etoolbox patching commands
(c:/texlive/2023/texmf-dist/tex/latex/l3packages/xparse/xparse.sty
Package: xparse 2024-02-18 L3 Experimental document command parser
)) (c:/texlive/2023/texmf-dist/tex/latex/environ/environ.sty
Package: environ 2014/05/04 v0.3 A new way to define environments
```

```
(c:/texlive/2023/texmf-dist/tex/latex/trimspaces/trimspaces.sty
Package: trimspaces 2009/09/17 v1.1 Trim spaces around a token list
)
\@envbody=\toks30
) (c:/texlive/2023/texmf-dist/tex/latex/lastpage/lastpage.sty
Package: lastpage 2023/10/14 v2.0e lastpage: 2.09 or 2e? (HMM)
(c:/texlive/2023/texmf-dist/tex/latex/lastpage/lastpage2e.sty
Package: lastpage2e 2023/10/14 v2.0e Decide which 2e lastpage version to
use (H
MM)
(c:/texlive/2023/texmf-dist/tex/latex/lastpage/lastpagemodern.sty
Package: lastpagemodern 2023-10-14 v2.0e Refers to last page's name (HMM;
JPG)
\c@lastpagecount=\count268
)
)) (c:/texlive/2023/texmf-dist/tex/latex/graphics/rotating.sty
Package: rotating 2016/08/11 v2.16d rotated objects in LaTeX
(c:/texlive/2023/texmf-dist/tex/latex/base/ifthen.sty
Package: ifthen 2022/04/13 v1.1d Standard LaTeX ifthen package (DPC)
)
\c@r@tfl@t=\count269
\rotFPtop=\skip68
\rotFPbot=\skip69
\rot@float@box=\box55
\rot@mess@toks=\toks31
) (c:/texlive/2023/texmf-dist/tex/latex/graphics/lscape.sty
Package: lscape 2020/05/28 v3.02 Landscape Pages (DPC)
) (c:/texlive/2023/texmf-dist/tex/latex/tools/afterpage.sty
Package: afterpage 2023/07/04 v1.08 After-Page Package (DPC)
\AP@output=\toks32
\AP@partial=\box56
\AP@footins=\box57
) (c:/texlive/2023/texmf-dist/tex/latex/textpos/textpos.sty
Package: textpos 2022/07/23 v1.10.1
Package textpos Info: choosing support for LaTeX3 on input line 60.
\TP@textbox=\box58
\TP@holdbox=\box59
\TPHorizModule=\dimen146
\TPVertModule=\dimen147
\TP@margin=\dimen148
\TP@absmargin=\dimen149
Grid set 16 x 16 = 37.34424pt x 52.81541pt
\TPboxrulesize=\dimen150
\TP@ox=\dimen151
\TP@oy=\dimen152
\TP@tbargs=\toks33
TextBlockOrigin set to 0pt x 0pt
) (c:/texlive/2023/texmf-dist/tex/latex/url/url.sty
\Urlmuskip=\muskip19
Package: url 2013/09/16  ver 3.4  Verb mode for urls, etc.
) (c:/texlive/2023/texmf-dist/tex/latex/newfloat/newfloat.sty
Package: newfloat 2023/10/01 v1.2 Defining new floating environments (AR)
Package newfloat Info: `rotating' package detected.
) (c:/texlive/2023/texmf-dist/tex/latex/mdframed/mdframed.sty
```

```
Package: mdframed 2013/07/01 1.9b: mdframed
(c:/texlive/2023/texmf-dist/tex/latex/kvoptions/kvoptions.sty
Package: kvoptions 2022-06-15 v3.15 Key value format for package options
(HO)
(c:/texlive/2023/texmf-dist/tex/generic/ltxcmds/ltxcmds.sty
Package: ltxcmds 2023-12-04 v1.26 LaTeX kernel commands for general use
(HO)
) (c:/texlive/2023/texmf-dist/tex/latex/kvsetkeys/kvsetkeys.sty
Package: kvsetkeys 2022-10-05 v1.19 Key value parser (HO)
)) (c:/texlive/2023/texmf-dist/tex/latex/zref/zref-abspage.sty
Package: zref-abspage 2023-09-14 v2.35 Module abspage for zref (HO)
(c:/texlive/2023/texmf-dist/tex/latex/zref/zref-base.sty
Package: zref-base 2023-09-14 v2.35 Module base for zref (HO)
(c:/texlive/2023/texmf-dist/tex/generic/infwarerr/infwarerr.sty
Package: infwarerr 2019/12/03 v1.5 Providing info/warning/error messages
(HO)
) (c:/texlive/2023/texmf-dist/tex/generic/kvdefinekeys/kvdefinekeys.sty
Package: kvdefinekeys 2019-12-19 v1.6 Define keys (HO)
) (c:/texlive/2023/texmf-dist/tex/generic/pdftexcmds/pdftexcmds.sty
Package: pdftexcmds 2020-06-27 v0.33 Utility functions of pdfTeX for
LuaTeX (HO
)
Package pdftexcmds Info: \pdf@primitive is available.
Package pdftexcmds Info: \pdf@ifprimitive is available.
Package pdftexcmds Info: \pdfdraftmode found.
) (c:/texlive/2023/texmf-dist/tex/generic/etexcmds/etexcmds.sty
Package: etexcmds 2019/12/15 v1.7 Avoid name clashes with e-TeX commands
(HO)
) (c:/texlive/2023/texmf-dist/tex/latex/auxhook/auxhook.sty
Package: auxhook 2019-12-17 v1.6 Hooks for auxiliary files (HO)
)
Package zref Info: New property list: main on input line 767.
Package zref Info: New property: default on input line 768.
Package zref Info: New property: page on input line 769.
)
\c@abspage=\count270
Package zref Info: New property: abspage on input line 67.
) (c:/texlive/2023/texmf-dist/tex/latex/needspace/needspace.sty
Package: needspace 2010/09/12 v1.3d reserve vertical space
)
\mdf@templength=\skip70
\c@mdf@globalstyle@cnt=\count271
\mdf@skipabove@length=\skip71
\mdf@skipbelow@length=\skip72
\mdf@leftmargin@length=\skip73
\mdf@rightmargin@length=\skip74
\mdf@innerleftmargin@length=\skip75
\mdf@innerrightmargin@length=\skip76
\mdf@innertopmargin@length=\skip77
\mdf@innerbottommargin@length=\skip78
\mdf@splittopskip@length=\skip79
\mdf@splitbottomskip@length=\skip80
\mdf@outermargin@length=\skip81
\mdf@innermargin@length=\skip82
```

```
\mdf@linewidth@length=\skip83
\mdf@innerlinewidth@length=\skip84
\mdf@middlelinewidth@length=\skip85
\mdf@outerlinewidth@length=\skip86
\mdf@roundcorner@length=\skip87
\mdf@footenotedistance@length=\skip88
\mdf@userdefinedwidth@length=\skip89
\mdf@needspace@length=\skip90
\mdf@frametitleaboveskip@length=\skip91
\mdf@frametitlebelowskip@length=\skip92
\mdf@frametitlerulewidth@length=\skip93
\mdf@frametitleleftmargin@length=\skip94
\mdf@frametitlerightmargin@length=\skip95
\mdf@shadowsize@length=\skip96
\mdf@extratopheight@length=\skip97
\mdf@subtitleabovelinewidth@length=\skip98
\mdf@subtitlebelowlinewidth@length=\skip99
\mdf@subtitleaboveskip@length=\skip100
\mdf@subtitlebelowskip@length=\skip101
\mdf@subtitleinneraboveskip@length=\skip102
\mdf@subtitleinnerbelowskip@length=\skip103
\mdf@subsubtitleabovelinewidth@length=\skip104
\mdf@subsubtitlebelowlinewidth@length=\skip105
\mdf@subsubtitleaboveskip@length=\skip106
\mdf@subsubtitlebelowskip@length=\skip107
\mdf@subsubtitleinneraboveskip@length=\skip108
\mdf@subsubtitleinnerbelowskip@length=\skip109
(c:/texlive/2023/texmf-dist/tex/latex/mdframed/md-frame-0.mdf
File: md-frame-0.mdf 2013/07/01\ 1.9b: md-frame-0
)
\mdf@frametitlebox=\box60
\mdf@footnotebox=\box61
\mdf@splitbox@one=\box62
\mdf@splitbox@two=\box63
\mdf@splitbox@save=\box64
\mdfsplitboxwidth=\skip110
\mdfsplitboxtotalwidth=\skip111
\mdfsplitboxheight=\skip112
\mdfsplitboxdepth=\skip113
\mdfsplitboxtotalheight=\skip114
\mdfframetitleboxwidth=\skip115
\mdfframetitleboxtotalwidth=\skip116
\mdfframetitleboxheight=\skip117
\mdfframetitleboxdepth=\skip118
\mdfframetitleboxtotalheight=\skip119
\mdffootnoteboxwidth=\skip120
\mdffootnoteboxtotalwidth=\skip121
\mdffootnoteboxheight=\skip122
\mdffootnoteboxdepth=\skip123
\mdffootnoteboxtotalheight=\skip124
\mdftotallinewidth=\skip125
\mdfboundingboxwidth=\skip126
\mdfboundingboxtotalwidth=\skip127
\mdfboundingboxheight=\skip128
```

```
\mdfboundingboxdepth=\skip129
\mdfboundingboxtotalheight=\skip130
\mdf@freevspace@length=\skip131
\mdf@horizontalwidthofbox@length=\skip132
\mdf@verticalmarginwhole@length=\skip133
\mdf@horizontalspaceofbox=\skip134
\mdfsubtitleheight=\skip135
\mdfsubsubtitleheight=\skip136
\c@mdfcountframes=\count272

****** mdframed patching \endmdf@trivlist

****** -- success******

\mdf@envdepth=\count273
\c@mdf@env@i=\count274
\c@mdf@env@ii=\count275
\c@mdf@zref@counter=\count276
Package zref Info: New property: mdf@pagevalue on input line 895.
) (c:/texlive/2023/texmf-dist/tex/latex/titlesec/titlesec.sty
Package: titlesec 2023/10/27 v2.16 Sectioning titles
\ttl@box=\box65
\beforetitleunit=\skip137
\aftertitleunit=\skip138
\ttl@plus=\dimen153
\ttl@minus=\dimen154
\ttl@toksa=\toks34
\titlewidth=\dimen155
\titlewidthlast=\dimen156
\titlewidthfirst=\dimen157
) (c:/texlive/2023/texmf-dist/tex/latex/koma-script/scrextend.sty
Package: scrextend 2023/07/07 v3.41 KOMA-Script package (extend other
classes w
ith features of KOMA-Script classes)
(c:/texlive/2023/texmf-dist/tex/latex/koma-script/scrkbase.sty
Package: scrkbase 2023/07/07 v3.41 KOMA-Script package (KOMA-Script-
dependent b
asics and keyval usage)
(c:/texlive/2023/texmf-dist/tex/latex/koma-script/scrbase.sty
Package: scrbase 2023/07/07 v3.41 KOMA-Script package (KOMA-Script-
independent
basics and keyval usage)
(c:/texlive/2023/texmf-dist/tex/latex/koma-script/scrlfile.sty
Package: scrlfile 2023/07/07 v3.41 KOMA-Script package (file load hooks)
(c:/texlive/2023/texmf-dist/tex/latex/koma-script/scrlfile-hook.sty
Package: scrlfile-hook 2023/07/07 v3.41 KOMA-Script package (using LaTeX
hooks)

(c:/texlive/2023/texmf-dist/tex/latex/koma-script/scrlogo.sty
Package: scrlogo 2023/07/07 v3.41 KOMA-Script package (logo)
)))
Applying: [2021/05/01] Usage of raw or classic option list on input line
252.
```

```
Already applied: [0000/00/00] Usage of raw or classic option list on
input line
 368.
))
Package scrextend Info: unexpected definition of `\@makefnmark'.
(scrextend)             Trying to patch it on input line 1762.
Package scrextend Info: patch seems to be successfull on input line 1762.
)

LaTeX Font Warning: Font shape `T1/cmr/m/n' in size <7.5> not available
(Font)              size <7> substituted on input line 65.

(c:/texlive/2023/texmf-dist/tex/latex/tools/calc.sty
Package: calc 2023/07/08 v4.3 Infix arithmetic (KKT,FJ)
\calc@Acount=\count277
\calc@Bcount=\count278
\calc@Adimen=\dimen158
\calc@Bdimen=\dimen159
\calc@Askip=\skip139
\calc@Bskip=\skip140
LaTeX Info: Redefining \setlength on input line 80.
LaTeX Info: Redefining \addtolength on input line 81.
\calc@Ccount=\count279
\calc@Cskip=\skip141
) (c:/texlive/2023/texmf-dist/tex/latex/geometry/geometry.sty
Package: geometry 2020/01/02 v5.9 Page Geometry
(c:/texlive/2023/texmf-dist/tex/generic/iftex/ifvtex.sty
Package: ifvtex 2019/10/25 v1.7 ifvtex legacy package. Use iftex instead.
)
\Gm@cnth=\count280
\Gm@cntv=\count281
\c@Gm@tempcnt=\count282
\Gm@bindingoffset=\dimen160
\Gm@wd@mp=\dimen161
\Gm@odd@mp=\dimen162
\Gm@even@mp=\dimen163
\Gm@layoutwidth=\dimen164
\Gm@layoutheight=\dimen165
\Gm@layouthoffset=\dimen166
\Gm@layoutvoffset=\dimen167
\Gm@dimlist=\toks35
) (c:/texlive/2023/texmf-dist/tex/latex/hyperref/hyperref.sty
Package: hyperref 2024-01-20 v7.01h Hypertext links for LaTeX
(c:/texlive/2023/texmf-dist/tex/generic/pdfescape/pdfescape.sty
Package: pdfescape 2019/12/09 v1.15 Implements pdfTeX's escape features
(HO)
) (c:/texlive/2023/texmf-dist/tex/latex/hycolor/hycolor.sty
Package: hycolor 2020-01-27 v1.10 Color options for hyperref/bookmark
(HO)
) (c:/texlive/2023/texmf-dist/tex/latex/hyperref/nameref.sty
Package: nameref 2023-11-26 v2.56 Cross-referencing by name of section
(c:/texlive/2023/texmf-dist/tex/latex/refcount/refcount.sty
Package: refcount 2019/12/15 v3.6 Data extraction from label references
(HO)
```

```
) (c:/texlive/2023/texmf-
dist/tex/generic/gettitlestring/gettitlestring.sty
Package: gettitlestring 2019/12/15 v1.6 Cleanup title references (HO)
)
\c@section@level=\count283
)
\@linkdim=\dimen168
\Hy@linkcounter=\count284
\Hy@pagecounter=\count285
(c:/texlive/2023/texmf-dist/tex/latex/hyperref/pd1enc.def
File: pd1enc.def 2024-01-20 v7.01h Hyperref: PDFDocEncoding definition
(HO)
Now handling font encoding PD1 ...
... no UTF-8 mapping file for font encoding PD1
) (c:/texlive/2023/texmf-dist/tex/generic/intcalc/intcalc.sty
Package: intcalc 2019/12/15 v1.3 Expandable calculations with integers
(HO)
)
\Hy@SavedSpaceFactor=\count286
(c:/texlive/2023/texmf-dist/tex/latex/hyperref/puenc.def
File: puenc.def 2024-01-20 v7.01h Hyperref: PDF Unicode definition (HO)
Now handling font encoding PU ...
... no UTF-8 mapping file for font encoding PU
)
Package hyperref Info: Option `colorlinks' set `true' on input line 4062.
Package hyperref Info: Hyper figures OFF on input line 4179.
Package hyperref Info: Link nesting OFF on input line 4184.
Package hyperref Info: Hyper index ON on input line 4187.
Package hyperref Info: Plain pages OFF on input line 4194.
Package hyperref Info: Backreferencing OFF on input line 4199.
Package hyperref Info: Implicit mode ON; LaTeX internals redefined.
Package hyperref Info: Bookmarks ON on input line 4446.
\c@Hy@tempcnt=\count287
LaTeX Info: Redefining \url on input line 4784.
\XeTeXLinkMargin=\dimen169
(c:/texlive/2023/texmf-dist/tex/generic/bitset/bitset.sty
Package: bitset 2019/12/09 v1.3 Handle bit-vector datatype (HO)
(c:/texlive/2023/texmf-dist/tex/generic/bigintcalc/bigintcalc.sty
Package: bigintcalc 2019/12/15 v1.5 Expandable calculations on big
integers (HO
)
))
\Fld@menulength=\count288
\Field@Width=\dimen170
\Fld@charsize=\dimen171
Package hyperref Info: Hyper figures OFF on input line 6063.
Package hyperref Info: Link nesting OFF on input line 6068.
Package hyperref Info: Hyper index ON on input line 6071.
Package hyperref Info: backreferencing OFF on input line 6078.
Package hyperref Info: Link coloring ON on input line 6081.
Package hyperref Info: Link coloring with OCG OFF on input line 6088.
Package hyperref Info: PDF/A mode OFF on input line 6093.
(c:/texlive/2023/texmf-dist/tex/latex/base/atbegshi-ltx.sty
Package: atbegshi-ltx 2021/01/10 v1.0c Emulation of the original atbegshi
```

```
package with kernel methods
)
\Hy@abspage=\count289
\c@Item=\count290
\c@Hfootnote=\count291
)
Package hyperref Info: Driver (autodetected): hpdftex.
(c:/texlive/2023/texmf-dist/tex/latex/hyperref/hpdftex.def
File: hpdftex.def 2024-01-20 v7.01h Hyperref driver for pdfTeX
(c:/texlive/2023/texmf-dist/tex/latex/base/atveryend-ltx.sty
Package: atveryend-ltx 2020/08/19 v1.0a Emulation of the original
atveryend pac
kage
with kernel methods
)
\HyAnn@Count=\count292
\Fld@listcount=\count293
\c@bookmark@seq@number=\count294
(c:/texlive/2023/texmf-dist/tex/latex/rerunfilecheck/rerunfilecheck.sty
Package: rerunfilecheck 2022-07-10 v1.10 Rerun checks for auxiliary files
(HO)
(c:/texlive/2023/texmf-dist/tex/generic/uniquecounter/uniquecounter.sty
Package: uniquecounter 2019/12/15 v1.4 Provide unlimited unique counter
(HO)
)
Package uniquecounter Info: New unique counter `rerunfilecheck' on input
line 2
85.
)
\Hy@SectionHShift=\skip142
) (c:/texlive/2023/texmf-dist/tex/latex/preprint/authblk.sty
Package: authblk 2001/02/27 1.3 (PWD)
\affilsep=\skip143
\@affilsep=\skip144
\c@Maxaffil=\count295
\c@authors=\count296
\c@affil=\count297
) (c:/texlive/2023/texmf-dist/tex/latex/footmisc/footmisc.sty
Package: footmisc 2023/07/05 v6.0f a miscellany of footnote facilities
\FN@temptoken=\toks36
\footnotemargin=\dimen172
\@outputbox@depth=\dimen173
Package footmisc Info: Declaring symbol style bringhurst on input line
696.
Package footmisc Info: Declaring symbol style chicago on input line 704.
Package footmisc Info: Declaring symbol style wiley on input line 713.
Package footmisc Info: Declaring symbol style lamport-robust on input
line 724.

Package footmisc Info: Declaring symbol style lamport* on input line 744.
Package footmisc Info: Declaring symbol style lamport*-robust on input
line 765
.
) (c:/texlive/2023/texmf-dist/tex/latex/fancyhdr/fancyhdr.sty
```

```
Package: fancyhdr 2022/11/09 v4.1 Extensive control of page headers and
footers

\f@nch@headwidth=\skip145
\f@nch@O@elh=\skip146
\f@nch@O@erh=\skip147
\f@nch@O@olh=\skip148
\f@nch@O@orh=\skip149
\f@nch@O@elf=\skip150
\f@nch@O@erf=\skip151
\f@nch@O@olf=\skip152
\f@nch@O@orf=\skip153
) (c:/texlive/2023/texmf-dist/tex/generic/alphalph/alphalph.sty
Package: alphalph 2019/12/09 v2.6 Convert numbers to letters (HO)
)
\c@authorfn=\count298
(c:/texlive/2023/texmf-dist/tex/latex/abstract/abstract.sty
Package: abstract 2009/06/08 v1.2a configurable abstracts
\abstitleskip=\skip154
\absleftindent=\skip155
\absrightindent=\skip156
\absparindent=\skip157
\absparsep=\skip158
)
Package newfloat Info: New float `keypoints' with options
`placement=t!,name=kp
t' on input line 286.
\c@keypoints=\count299
\newfloat@ftype=\count300
Package newfloat Info: float type `keypoints'=8 on input line 286.
(c:/texlive/2023/texmf-dist/tex/latex/enumitem/enumitem.sty
Package: enumitem 2019/06/20 v3.9 Customized lists
\labelindent=\skip159
\enit@outerparindent=\dimen174
\enit@toks=\toks37
\enit@inbox=\box66
\enit@count@id=\count301
\enitdp@description=\count302
) (c:/texlive/2023/texmf-dist/tex/latex/quoting/quoting.sty
Package: quoting 2014/01/28 v0.1c Consolidated environment for displayed
text
\quo@toppartop=\skip160
) (c:/texlive/2023/texmf-dist/tex/latex/sttools/stfloats.sty
Package: stfloats 2017/03/27 v3.3 Improve float mechanism and
baselineskip sett
ings
\@dblbotnum=\count303
\c@dblbotnumber=\count304
) (c:/texlive/2023/texmf-dist/tex/latex/booktabs/booktabs.sty
Package: booktabs 2020/01/12 v1.61803398 Publication quality tables
\heavyrulewidth=\dimen175
\lightrulewidth=\dimen176
\cmidrulewidth=\dimen177
\belowrulesep=\dimen178
```

```
\belowbottomsep=\dimen179
\aboverulesep=\dimen180
\abovetopsep=\dimen181
\cmidrulesep=\dimen182
\cmidrulekern=\dimen183
\defaultaddspace=\dimen184
\@cmidla=\count305
\@cmidlb=\count306
\@aboverulesep=\dimen185
\@belowrulesep=\dimen186
\@thisruleclass=\count307
\@lastruleclass=\count308
\@thisrulewidth=\dimen187
) (c:/texlive/2023/texmf-dist/tex/latex/tools/tabularx.sty
Package: tabularx 2023/07/08 v2.11c `tabularx' package (DPC)
\TX@col@width=\dimen188
\TX@old@table=\dimen189
\TX@old@col=\dimen190
\TX@target=\dimen191
\TX@delta=\dimen192
\TX@cols=\count309
\TX@ftn=\toks38
)
\enitdp@tablenotes=\count310
(c:/texlive/2023/texmf-dist/tex/latex/caption/caption.sty
Package: caption 2023/08/05 v3.6o Customizing captions (AR)
(c:/texlive/2023/texmf-dist/tex/latex/caption/caption3.sty
Package: caption3 2023/07/31 v2.4d caption3 kernel (AR)
\caption@tempdima=\dimen193
\captionmargin=\dimen194
\caption@leftmargin=\dimen195
\caption@rightmargin=\dimen196
\caption@width=\dimen197
\caption@indent=\dimen198
\caption@parindent=\dimen199
\caption@hangindent=\dimen256
Package caption Info: Standard document class detected.
)
\c@caption@flags=\count311
\c@continuedfloat=\count312
Package caption Info: hyperref package is loaded.
Package caption Info: rotating package is loaded.
Package caption Info: scrextend package is loaded.
\caption@addmargin@hsize=\dimen257
\caption@addmargin@linewidth=\dimen258
) (c:/texlive/2023/texmf-dist/tex/latex/natbib/natbib.sty
Package: natbib 2010/09/13 8.31b (PWD, AO)
\bibhang=\skip161
\bibsep=\skip162
LaTeX Info: Redefining \cite on input line 694.
\c@NAT@ctr=\count313
)) (c:/texlive/2023/texmf-dist/tex/latex/siunitx/siunitx.sty
Package: siunitx 2024-02-15 v3.3.12 A comprehensive (SI) units package
\l__siunitx_number_uncert_offset_int=\count314
```

```
\l__siunitx_number_exponent_fixed_int=\count315
\l__siunitx_number_min_decimal_int=\count316
\l__siunitx_number_min_integer_int=\count317
\l__siunitx_number_round_precision_int=\count318
\l__siunitx_number_lower_threshold_int=\count319
\l__siunitx_number_upper_threshold_int=\count320
\l__siunitx_number_group_first_int=\count321
\l__siunitx_number_group_size_int=\count322
\l__siunitx_number_group_minimum_int=\count323
\l__siunitx_angle_tmp_dim=\dimen259
\l__siunitx_angle_marker_box=\box67
\l__siunitx_angle_unit_box=\box68
\l__siunitx_compound_count_int=\count324
(c:/texlive/2023/texmf-dist/tex/latex/translations/translations.sty
Package: translations 2022/02/05 v1.12 internationalization of LaTeX2e
packages
 (CN)
) (c:/texlive/2023/texmf-dist/tex/latex/amsmath/amstext.sty
Package: amstext 2021/08/26 v2.01 AMS text
(c:/texlive/2023/texmf-dist/tex/latex/amsmath/amsgen.sty
File: amsgen.sty 1999/11/30 v2.0 generic functions
\@emptytoks=\toks39
\ex@=\dimen260
))
\l__siunitx_table_tmp_box=\box69
\l__siunitx_table_tmp_dim=\dimen261
\l__siunitx_table_column_width_dim=\dimen262
\l__siunitx_table_integer_box=\box70
\l__siunitx_table_decimal_box=\box71
\l__siunitx_table_uncert_box=\box72
\l__siunitx_table_before_box=\box73
\l__siunitx_table_after_box=\box74
\l__siunitx_table_before_dim=\dimen263
\l__siunitx_table_carry_dim=\dimen264
\l__siunitx_unit_tmp_int=\count325
\l__siunitx_unit_position_int=\count326
\l__siunitx_unit_total_int=\count327
) (c:/texlive/2023/texmf-dist/tex/latex/placeins/placeins.sty
Package: placeins 2005/04/18  v 2.2
) (./orcidlink.sty
Package: orcidlink 2024/06/26 v1.1.0 Support ORCID's three different ID
formats
.
(c:/texlive/2023/texmf-dist/tex/latex/pgf/frontendlayer/tikz.sty
(c:/texlive/20
23/texmf-dist/tex/latex/pgf/basiclayer/pgf.sty (c:/texlive/2023/texmf-
dist/tex/
latex/pgf/utilities/pgfrcs.sty (c:/texlive/2023/texmf-
dist/tex/generic/pgf/util
ities/pgfutil-common.tex
\pgfutil@everybye=\toks40
\pgfutil@tempdima=\dimen265
\pgfutil@tempdimb=\dimen266
) (c:/texlive/2023/texmf-dist/tex/generic/pgf/utilities/pgfutil-latex.def
```

```
\pgfutil@abb=\box75
) (c:/texlive/2023/texmf-dist/tex/generic/pgf/utilities/pgfrcs.code.tex
(c:/tex
live/2023/texmf-dist/tex/generic/pgf/pgf.revision.tex)
Package: pgfrcs 2023-01-15 v3.1.10 (3.1.10)
))
Package: pgf 2023-01-15 v3.1.10 (3.1.10)
(c:/texlive/2023/texmf-dist/tex/latex/pgf/basiclayer/pgfcore.sty
(c:/texlive/20
23/texmf-dist/tex/latex/pgf/systemlayer/pgfsys.sty
(c:/texlive/2023/texmf-dist/
tex/generic/pgf/systemlayer/pgfsys.code.tex
Package: pgfsys 2023-01-15 v3.1.10 (3.1.10)
(c:/texlive/2023/texmf-dist/tex/generic/pgf/utilities/pgfkeys.code.tex
\pgfkeys@pathtoks=\toks41
\pgfkeys@temptoks=\toks42

(c:/texlive/2023/texmf-
dist/tex/generic/pgf/utilities/pgfkeyslibraryfiltered.co
de.tex
\pgfkeys@tmptoks=\toks43
))
\pgf@x=\dimen267
\pgf@y=\dimen268
\pgf@xa=\dimen269
\pgf@ya=\dimen270
\pgf@xb=\dimen271
\pgf@yb=\dimen272
\pgf@xc=\dimen273
\pgf@yc=\dimen274
\pgf@xd=\dimen275
\pgf@yd=\dimen276
\w@pgf@writea=\write3
\r@pgf@reada=\read2
\c@pgf@counta=\count328
\c@pgf@countb=\count329
\c@pgf@countc=\count330
\c@pgf@countd=\count331
\t@pgf@toka=\toks44
\t@pgf@tokb=\toks45
\t@pgf@tokc=\toks46
\pgf@sys@id@count=\count332
(c:/texlive/2023/texmf-dist/tex/generic/pgf/systemlayer/pgf.cfg
File: pgf.cfg 2023-01-15 v3.1.10 (3.1.10)
)
Driver file for pgf: pgfsys-pdftex.def
(c:/texlive/2023/texmf-dist/tex/generic/pgf/systemlayer/pgfsys-pdftex.def
File: pgfsys-pdftex.def 2023-01-15 v3.1.10 (3.1.10)
(c:/texlive/2023/texmf-dist/tex/generic/pgf/systemlayer/pgfsys-common-
pdf.def
File: pgfsys-common-pdf.def 2023-01-15 v3.1.10 (3.1.10)
)))
(c:/texlive/2023/texmf-
dist/tex/generic/pgf/systemlayer/pgfsyssoftpath.code.tex
```

```
File: pgfsyssoftpath.code.tex 2023-01-15 v3.1.10 (3.1.10)
\pgfsyssoftpath@smallbuffer@items=\count333
\pgfsyssoftpath@bigbuffer@items=\count334
)
(c:/texlive/2023/texmf-
dist/tex/generic/pgf/systemlayer/pgfsysprotocol.code.tex
File: pgfsysprotocol.code.tex 2023-01-15 v3.1.10 (3.1.10)
)) (c:/texlive/2023/texmf-
dist/tex/generic/pgf/basiclayer/pgfcore.code.tex
Package: pgfcore 2023-01-15 v3.1.10 (3.1.10)
(c:/texlive/2023/texmf-dist/tex/generic/pgf/math/pgfmath.code.tex
(c:/texlive/2
023/texmf-dist/tex/generic/pgf/math/pgfmathutil.code.tex)
(c:/texlive/2023/texm
f-dist/tex/generic/pgf/math/pgfmathparser.code.tex
\pgfmath@dimen=\dimen277
\pgfmath@count=\count335
\pgfmath@box=\box76
\pgfmath@toks=\toks47
\pgfmath@stack@operand=\toks48
\pgfmath@stack@operation=\toks49
) (c:/texlive/2023/texmf-
dist/tex/generic/pgf/math/pgfmathfunctions.code.tex)
(c:/texlive/2023/texmf-
dist/tex/generic/pgf/math/pgfmathfunctions.basic.code.te
x)
(c:/texlive/2023/texmf-
dist/tex/generic/pgf/math/pgfmathfunctions.trigonometric
.code.tex)
(c:/texlive/2023/texmf-
dist/tex/generic/pgf/math/pgfmathfunctions.random.code.t
ex)
(c:/texlive/2023/texmf-
dist/tex/generic/pgf/math/pgfmathfunctions.comparison.co
de.tex)
(c:/texlive/2023/texmf-
dist/tex/generic/pgf/math/pgfmathfunctions.base.code.tex
)
(c:/texlive/2023/texmf-
dist/tex/generic/pgf/math/pgfmathfunctions.round.code.te
x)
(c:/texlive/2023/texmf-
dist/tex/generic/pgf/math/pgfmathfunctions.misc.code.tex
)
(c:/texlive/2023/texmf-
dist/tex/generic/pgf/math/pgfmathfunctions.integerarithm
etics.code.tex) (c:/texlive/2023/texmf-
dist/tex/generic/pgf/math/pgfmathcalc.co
de.tex) (c:/texlive/2023/texmf-
dist/tex/generic/pgf/math/pgfmathfloat.code.tex
\c@pgfmathroundto@lastzeros=\count336
)) (c:/texlive/2023/texmf-dist/tex/generic/pgf/math/pgfint.code.tex)
(c:/texliv
e/2023/texmf-dist/tex/generic/pgf/basiclayer/pgfcorepoints.code.tex
```

```
File: pgfcorepoints.code.tex 2023-01-15 v3.1.10 (3.1.10)
\pgf@picminx=\dimen278
\pgf@picmaxx=\dimen279
\pgf@picminy=\dimen280
\pgf@picmaxy=\dimen281
\pgf@pathminx=\dimen282
\pgf@pathmaxx=\dimen283
\pgf@pathminy=\dimen284
\pgf@pathmaxy=\dimen285
\pgf@xx=\dimen286
\pgf@xy=\dimen287
\pgf@yx=\dimen288
\pgf@yy=\dimen289
\pgf@zx=\dimen290
\pgf@zy=\dimen291
)
(c:/texlive/2023/texmf-
dist/tex/generic/pgf/basiclayer/pgfcorepathconstruct.cod
e.tex
File: pgfcorepathconstruct.code.tex 2023-01-15 v3.1.10 (3.1.10)
\pgf@path@lastx=\dimen292
\pgf@path@lasty=\dimen293
)
(c:/texlive/2023/texmf-
dist/tex/generic/pgf/basiclayer/pgfcorepathusage.code.te
x
File: pgfcorepathusage.code.tex 2023-01-15 v3.1.10 (3.1.10)
\pgf@shorten@end@additional=\dimen294
\pgf@shorten@start@additional=\dimen295
) (c:/texlive/2023/texmf-
dist/tex/generic/pgf/basiclayer/pgfcorescopes.code.tex
File: pgfcorescopes.code.tex 2023-01-15 v3.1.10 (3.1.10)
\pgfpic=\box77
\pgf@hbox=\box78
\pgf@layerbox@main=\box79
\pgf@picture@serial@count=\count337
)
(c:/texlive/2023/texmf-
dist/tex/generic/pgf/basiclayer/pgfcoregraphicstate.code
.tex
File: pgfcoregraphicstate.code.tex 2023-01-15 v3.1.10 (3.1.10)
\pgflinewidth=\dimen296
)
(c:/texlive/2023/texmf-
dist/tex/generic/pgf/basiclayer/pgfcoretransformations.c
ode.tex
File: pgfcoretransformations.code.tex 2023-01-15 v3.1.10 (3.1.10)
\pgf@pt@x=\dimen297
\pgf@pt@y=\dimen298
\pgf@pt@temp=\dimen299
) (c:/texlive/2023/texmf-
dist/tex/generic/pgf/basiclayer/pgfcorequick.code.tex
File: pgfcorequick.code.tex 2023-01-15 v3.1.10 (3.1.10)
```

```
) (c:/texlive/2023/texmf-
dist/tex/generic/pgf/basiclayer/pgfcoreobjects.code.te
x
File: pgfcoreobjects.code.tex 2023-01-15 v3.1.10 (3.1.10)
)
(c:/texlive/2023/texmf-
dist/tex/generic/pgf/basiclayer/pgfcorepathprocessing.co
de.tex
File: pgfcorepathprocessing.code.tex 2023-01-15 v3.1.10 (3.1.10)
) (c:/texlive/2023/texmf-
dist/tex/generic/pgf/basiclayer/pgfcorearrows.code.tex
File: pgfcorearrows.code.tex 2023-01-15 v3.1.10 (3.1.10)
\pgfarrowsep=\dimen300
) (c:/texlive/2023/texmf-
dist/tex/generic/pgf/basiclayer/pgfcoreshade.code.tex
File: pgfcoreshade.code.tex 2023-01-15 v3.1.10 (3.1.10)
\pgf@max=\dimen301
\pgf@sys@shading@range@num=\count338
\pgf@shadingcount=\count339
) (c:/texlive/2023/texmf-
dist/tex/generic/pgf/basiclayer/pgfcoreimage.code.tex
File: pgfcoreimage.code.tex 2023-01-15 v3.1.10 (3.1.10)
)
(c:/texlive/2023/texmf-
dist/tex/generic/pgf/basiclayer/pgfcoreexternal.code.tex
File: pgfcoreexternal.code.tex 2023-01-15 v3.1.10 (3.1.10)
\pgfexternal@startupbox=\box80
) (c:/texlive/2023/texmf-
dist/tex/generic/pgf/basiclayer/pgfcorelayers.code.tex
File: pgfcorelayers.code.tex 2023-01-15 v3.1.10 (3.1.10)
)
(c:/texlive/2023/texmf-
dist/tex/generic/pgf/basiclayer/pgfcoretransparency.code
.tex
File: pgfcoretransparency.code.tex 2023-01-15 v3.1.10 (3.1.10)
)
(c:/texlive/2023/texmf-
dist/tex/generic/pgf/basiclayer/pgfcorepatterns.code.tex
File: pgfcorepatterns.code.tex 2023-01-15 v3.1.10 (3.1.10)
) (c:/texlive/2023/texmf-
dist/tex/generic/pgf/basiclayer/pgfcorerdf.code.tex
File: pgfcorerdf.code.tex 2023-01-15 v3.1.10 (3.1.10)
))) (c:/texlive/2023/texmf-
dist/tex/generic/pgf/modules/pgfmoduleshapes.code.te
x
File: pgfmoduleshapes.code.tex 2023-01-15 v3.1.10 (3.1.10)
\pgfnodeparttextbox=\box81
) (c:/texlive/2023/texmf-
dist/tex/generic/pgf/modules/pgfmoduleplot.code.tex
File: pgfmoduleplot.code.tex 2023-01-15 v3.1.10 (3.1.10)
)
(c:/texlive/2023/texmf-dist/tex/latex/pgf/compatibility/pgfcomp-version-
0-65.st
y
```

```
Package: pgfcomp-version-0-65 2023-01-15 v3.1.10 (3.1.10)
\pgf@nodesepstart=\dimen302
\pgf@nodesepend=\dimen303
)
(c:/texlive/2023/texmf-dist/tex/latex/pgf/compatibility/pgfcomp-version-
1-18.st
y
Package: pgfcomp-version-1-18 2023-01-15 v3.1.10 (3.1.10)
)) (c:/texlive/2023/texmf-dist/tex/latex/pgf/utilities/pgffor.sty
(c:/texlive/2
023/texmf-dist/tex/latex/pgf/utilities/pgfkeys.sty
(c:/texlive/2023/texmf-dist/
tex/generic/pgf/utilities/pgfkeys.code.tex)) (c:/texlive/2023/texmf-
dist/tex/la
tex/pgf/math/pgfmath.sty (c:/texlive/2023/texmf-
dist/tex/generic/pgf/math/pgfma
th.code.tex)) (c:/texlive/2023/texmf-
dist/tex/generic/pgf/utilities/pgffor.code
.tex
Package: pgffor 2023-01-15 v3.1.10 (3.1.10)
\pgffor@iter=\dimen304
\pgffor@skip=\dimen305
\pgffor@stack=\toks50
\pgffor@toks=\toks51
)) (c:/texlive/2023/texmf-
dist/tex/generic/pgf/frontendlayer/tikz/tikz.code.tex
Package: tikz 2023-01-15 v3.1.10 (3.1.10)

(c:/texlive/2023/texmf-
dist/tex/generic/pgf/libraries/pgflibraryplothandlers.co
de.tex
File: pgflibraryplothandlers.code.tex 2023-01-15 v3.1.10 (3.1.10)
\pgf@plot@mark@count=\count340
\pgfplotmarksize=\dimen306
)
\tikz@lastx=\dimen307
\tikz@lasty=\dimen308
\tikz@lastxsaved=\dimen309
\tikz@lastysaved=\dimen310
\tikz@lastmovetox=\dimen311
\tikz@lastmovetoy=\dimen312
\tikzleveldistance=\dimen313
\tikzsiblingdistance=\dimen314
\tikz@figbox=\box82
\tikz@figbox@bg=\box83
\tikz@tempbox=\box84
\tikz@tempbox@bg=\box85
\tikztreelevel=\count341
\tiknumberofchildren=\count342
\tikznumberofcurrentchild=\count343
\tikz@fig@count=\count344
(c:/texlive/2023/texmf-
dist/tex/generic/pgf/modules/pgfmodulematrix.code.tex
File: pgfmodulematrix.code.tex 2023-01-15 v3.1.10 (3.1.10)
```

```
\pgfmatrixcurrentrow=\count345
\pgfmatrixcurrentcolumn=\count346
\pgf@matrix@numberofcolumns=\count347
)
\tikz@expandcount=\count348

(c:/texlive/2023/texmf-
dist/tex/generic/pgf/frontendlayer/tikz/libraries/tikzli
brarytopaths.code.tex
File: tikzlibrarytopaths.code.tex 2023-01-15 v3.1.10 (3.1.10)
)))
(c:/texlive/2023/texmf-
dist/tex/generic/pgf/frontendlayer/tikz/libraries/tikzli
brarysvg.path.code.tex
File: tikzlibrarysvg.path.code.tex 2023-01-15 v3.1.10 (3.1.10)

(c:/texlive/2023/texmf-
dist/tex/generic/pgf/libraries/pgflibrarysvg.path.code.t
ex
File: pgflibrarysvg.path.code.tex 2023-01-15 v3.1.10 (3.1.10)
(c:/texlive/2023/texmf-
dist/tex/generic/pgf/modules/pgfmoduleparser.code.tex
File: pgfmoduleparser.code.tex 2023-01-15 v3.1.10 (3.1.10)
\pgfparserdef@arg@count=\count349
)
\pgf@lib@svg@last@x=\dimen315
\pgf@lib@svg@last@y=\dimen316
\pgf@lib@svg@last@c@x=\dimen317
\pgf@lib@svg@last@c@y=\dimen318
\pgf@lib@svg@count=\count350
\pgf@lib@svg@max@num=\count351
))
\@curXheight=\skip163
) (c:/texlive/2023/texmf-dist/tex/latex/lineno/lineno.sty
Package: lineno 2023/05/20 line numbers on paragraphs v5.3
\linenopenalty=\count352
\output=\toks52
\linenoprevgraf=\count353
\linenumbersep=\dimen319
\linenumberwidth=\dimen320
\c@linenumber=\count354
\c@pagewiselinenumber=\count355
\c@LN@truepage=\count356
\c@internallinenumber=\count357
\c@internallinenumbers=\count358
\quotelinenumbersep=\dimen321
\bframerule=\dimen322
\bframesep=\dimen323
\bframebox=\box86
LaTeX Info: Redefining \\ on input line 3180.
)
Package translations Info: No language package found. I am going to use
`englis
h' as default language. on input line 66.
```

```
LaTeX Font Info:    Trying to load font information for T1+Merriwthr-OsF
on inp
ut line 66.
(c:/texlive/2023/texmf-dist/tex/latex/merriweather/T1Merriwthr-OsF.fd
File: T1Merriwthr-OsF.fd 2020/08/30 (autoinst) Font definitions for
T1/Merriwth
r-OsF.
)
LaTeX Font Info:    Font shape `T1/Merriwthr-OsF/m/n' will be
(Font)              scaled to size 7.5pt on input line 66.
(./main.aux)
\openout1 = `main.aux'.

LaTeX Font Info:    Checking defaults for OML/cmm/m/it on input line 66.
LaTeX Font Info:    ... okay on input line 66.
LaTeX Font Info:    Checking defaults for OMS/cmsy/m/n on input line 66.
LaTeX Font Info:    ... okay on input line 66.
LaTeX Font Info:    Checking defaults for OT1/cmr/m/n on input line 66.
LaTeX Font Info:    ... okay on input line 66.
LaTeX Font Info:    Checking defaults for T1/cmr/m/n on input line 66.
LaTeX Font Info:    ... okay on input line 66.
LaTeX Font Info:    Checking defaults for TS1/cmr/m/n on input line 66.
LaTeX Font Info:    ... okay on input line 66.
LaTeX Font Info:    Checking defaults for OMX/cmex/m/n on input line 66.
LaTeX Font Info:    ... okay on input line 66.
LaTeX Font Info:    Checking defaults for U/cmr/m/n on input line 66.
LaTeX Font Info:    ... okay on input line 66.
LaTeX Font Info:    Checking defaults for PD1/pdf/m/n on input line 66.
LaTeX Font Info:    ... okay on input line 66.
LaTeX Font Info:    Checking defaults for PU/pdf/m/n on input line 66.
LaTeX Font Info:    ... okay on input line 66.
LaTeX Info: Redefining \microtypecontext on input line 66.
Package microtype Info: Applying patch `item' on input line 66.
Package microtype Info: Applying patch `toc' on input line 66.
Package microtype Info: Applying patch `eqnum' on input line 66.

Package microtype Warning: Unable to apply patch `footnote' on input line
66.

Package microtype Info: Applying patch `verbatim' on input line 66.
Package microtype Info: Generating PDF output.
Package microtype Info: Character protrusion enabled (level 2).
Package microtype Info: Using default protrusion set `alltext'.
Package microtype Info: Automatic font expansion enabled (level 2),
(microtype)             stretch: 20, shrink: 20, step: 1, non-selected.
Package microtype Info: Using default expansion set `alltext-nott'.
LaTeX Info: Redefining \showhyphens on input line 66.
Package microtype Info: No adjustment of tracking.
Package microtype Info: No adjustment of interword spacing.
Package microtype Info: No adjustment of character kerning.
Package microtype Info: Loading generic protrusion settings for font
family
(microtype)             `Merriwthr-OsF' (encoding: T1).
```

```
(microtype)              For optimal results, create family-specific
settings.
(microtype)              See the microtype manual for details.
LaTeX Font Info:    Redeclaring symbol font `operators' on input line 66.
LaTeX Font Info:    Encoding `OT1' has changed to `T1' for symbol font
(Font)              `operators' in the math version `normal' on input
line 66.
LaTeX Font Info:    Overwriting symbol font `operators' in version
`normal'
(Font)                  OT1/cmr/m/n --> T1/Merriwthr-OsF/m/up on input
line 66.

LaTeX Font Info:    Encoding `OT1' has changed to `T1' for symbol font
(Font)              `operators' in the math version `bold' on input line
66.
LaTeX Font Info:    Overwriting symbol font `operators' in version `bold'
(Font)                  OT1/cmr/bx/n --> T1/Merriwthr-OsF/m/up on input
line 66
.
LaTeX Font Info:    Overwriting symbol font `operators' in version `bold'
(Font)                  T1/Merriwthr-OsF/m/up --> T1/Merriwthr-OsF/b/up
on inpu
t line 66.
LaTeX Font Info:    Redeclaring math alphabet \mathbf on input line 66.
LaTeX Font Info:    Overwriting math alphabet `\mathbf' in version
`normal'
(Font)                  OT1/cmr/bx/n --> T1/Merriwthr-OsF/b/up on input
line 66
.
LaTeX Font Info:    Overwriting math alphabet `\mathbf' in version `bold'
(Font)                  OT1/cmr/bx/n --> T1/Merriwthr-OsF/b/up on input
line 66
.
LaTeX Font Info:    Redeclaring math alphabet \mathsf on input line 66.
LaTeX Font Info:    Overwriting math alphabet `\mathsf' in version
`normal'
(Font)                  OT1/cmss/m/n --> T1/MerriwthrSans-OsF/m/up on
input lin
e 66.
LaTeX Font Info:    Overwriting math alphabet `\mathsf' in version `bold'
(Font)                  OT1/cmss/bx/n --> T1/MerriwthrSans-OsF/m/up on
input li
ne 66.
LaTeX Font Info:    Redeclaring math alphabet \mathit on input line 66.
LaTeX Font Info:    Overwriting math alphabet `\mathit' in version
`normal'
(Font)                  OT1/cmr/m/it --> T1/Merriwthr-OsF/m/it on input
line 66
.
LaTeX Font Info:    Overwriting math alphabet `\mathit' in version `bold'
(Font)                  OT1/cmr/bx/it --> T1/Merriwthr-OsF/m/it on input
line 6
6.
LaTeX Font Info:    Redeclaring math alphabet \mathtt on input line 66.
```

```
LaTeX Font Info:    Overwriting math alphabet `\mathtt' in version
`normal'
(Font)                  OT1/cmtt/m/n --> T1/lmtt/m/up on input line 66.
LaTeX Font Info:    Overwriting math alphabet `\mathtt' in version `bold'
(Font)                  OT1/cmtt/m/n --> T1/lmtt/m/up on input line 66.
LaTeX Font Info:    Overwriting math alphabet `\mathsf' in version `bold'
(Font)                  T1/MerriwthrSans-OsF/m/up --> T1/MerriwthrSans-
OsF/b/up
 on input line 66.
LaTeX Font Info:    Overwriting math alphabet `\mathit' in version `bold'
(Font)                  T1/Merriwthr-OsF/m/it --> T1/Merriwthr-OsF/b/it
on inpu
t line 66.
\c@mv@tabular=\count359
\c@mv@boldtabular=\count360
(c:/texlive/2023/texmf-dist/tex/context/base/mkii/supp-pdf.mkii
[Loading MPS to PDF converter (version 2006.09.02).]
\scratchcounter=\count361
\scratchdimen=\dimen324
\scratchbox=\box87
\nofMPsegments=\count362
\nofMParguments=\count363
\everyMPshowfont=\toks53
\MPscratchCnt=\count364
\MPscratchDim=\dimen325
\MPnumerator=\count365
\makeMPintoPDFobject=\count366
\everyMPtoPDFconversion=\toks54
) (c:/texlive/2023/texmf-dist/tex/latex/epstopdf-pkg/epstopdf-base.sty
Package: epstopdf-base 2020-01-24 v2.11 Base part for package epstopdf
Package epstopdf-base Info: Redefining graphics rule for `.eps' on input
line 4
85.
(c:/texlive/2023/texmf-dist/tex/latex/latexconfig/epstopdf-sys.cfg
File: epstopdf-sys.cfg 2010/07/13 v1.3 Configuration of (r)epstopdf for
TeX Liv
e
))
*geometry* driver: auto-detecting
*geometry* detected driver: pdftex
*geometry* verbose mode - [ preamble ] result:
* driver: pdftex
* paper: a4paper
* layout: <same size as paper>
* layoutoffset:(h,v)=(0.0pt,0.0pt)
* modes: includefoot twoside
* h-part:(L,W,R)=(54.64pt, 488.22787pt, 54.64pt)
* v-part:(T,H,B)=(66.0pt, 745.04684pt, 34.0pt)
* \paperwidth=597.50787pt
* \paperheight=845.04684pt
* \textwidth=488.22787pt
* \textheight=715.04684pt
* \oddsidemargin=-17.62999pt
* \evensidemargin=-17.62999pt
```

```
* \topmargin=-47.76999pt
* \headheight=17.5pt
* \headsep=24.0pt
* \topskip=10.0pt
* \footskip=30.0pt
* \marginparwidth=48.0pt
* \marginparsep=10.0pt
* \columnsep=18.0pt
* \skip\footins=22.0pt plus 2.0pt
* \hoffset=0.0pt
* \voffset=0.0pt
* \mag=1000
* \@twocolumntrue
* \@twosidetrue
* \@mparswitchtrue
* \@reversemarginfalse
* (1in=72.27pt=25.4mm, 1cm=28.453pt)

Package hyperref Info: Link coloring ON on input line 66.
(./main.out) (./main.out)
\@outlinefile=\write4
\openout4 = `main.out'.

\@gscitedetails=\box88
\@gscitedetailsheight=\skip164
\@gsheadbox=\box89
\@gsheadboxheight=\skip165
LaTeX Font Info:    Font shape `T1/Merriwthr-OsF/b/n' will be
(Font)              scaled to size 6.5pt on input line 66.
LaTeX Font Info:    Calculating math sizes for size <7.5> on input line
66.

LaTeX Font Warning: Font shape `T1/Merriwthr-OsF/m/up' undefined
(Font)              using `T1/Merriwthr-OsF/m/n' instead on input line
66.

LaTeX Font Info:    Font shape `T1/Merriwthr-OsF/m/up' will be
(Font)              scaled to size 6.24973pt on input line 66.
LaTeX Font Info:    Font shape `T1/Merriwthr-OsF/m/up' will be
(Font)              scaled to size 5.24997pt on input line 66.
LaTeX Font Info:    Trying to load font information for U+eur on input
line 66.

(c:/texlive/2023/texmf-dist/tex/latex/amsfonts/ueur.fd
File: ueur.fd 2013/01/14 v3.01 Euler Roman
) (c:/texlive/2023/texmf-dist/tex/latex/microtype/mt-eur.cfg
File: mt-eur.cfg 2006/07/31 v1.1 microtype config. file: AMS Euler Roman
(RS)
)

LaTeX Font Warning: Font shape `OMS/cmsy/m/n' in size <7.5> not available
(Font)              size <7> substituted on input line 66.

LaTeX Font Info:    External font `cmex10' loaded for size
```

```
(Font)              <7.5> on input line 66.
LaTeX Font Info:    External font `cmex10' loaded for size
(Font)              <6.24973> on input line 66.
LaTeX Font Info:    External font `cmex10' loaded for size
(Font)              <5.24997> on input line 66.
LaTeX Font Info:    Trying to load font information for U+euf on input
line 66.

(c:/texlive/2023/texmf-dist/tex/latex/amsfonts/ueuf.fd
File: ueuf.fd 2013/01/14 v3.01 Euler Fraktur
) (c:/texlive/2023/texmf-dist/tex/latex/microtype/mt-euf.cfg
File: mt-euf.cfg 2006/07/03 v1.1 microtype config. file: AMS Euler
Fraktur (RS)

)
LaTeX Font Info:    Trying to load font information for U+eus on input
line 66.

(c:/texlive/2023/texmf-dist/tex/latex/amsfonts/ueus.fd
File: ueus.fd 2013/01/14 v3.01 Euler Script
) (c:/texlive/2023/texmf-dist/tex/latex/microtype/mt-eus.cfg
File: mt-eus.cfg 2006/07/28 v1.2 microtype config. file: AMS Euler Script
(RS)
)
LaTeX Font Info:    Trying to load font information for U+euex on input
line 66
.
(c:/texlive/2023/texmf-dist/tex/latex/amsfonts/ueuex.fd
File: ueuex.fd 2013/01/14 v3.01 Euler extra symbols
)

LaTeX Font Warning: Font shape `OML/cmm/m/it' in size <7.5> not available
(Font)              size <7> substituted on input line 66.

LaTeX Font Info:    Font shape `T1/Merriwthr-OsF/m/n' will be
(Font)              scaled to size 6.24973pt on input line 66.
LaTeX Font Info:    Font shape `T1/Merriwthr-OsF/m/n' will be
(Font)              scaled to size 5.24997pt on input line 66.
LaTeX Font Info:    Font shape `T1/Merriwthr-OsF/m/it' will be
(Font)              scaled to size 7.5pt on input line 66.
LaTeX Font Info:    Font shape `T1/Merriwthr-OsF/m/it' will be
(Font)              scaled to size 6.24973pt on input line 66.
LaTeX Font Info:    Font shape `T1/Merriwthr-OsF/m/it' will be
(Font)              scaled to size 5.24997pt on input line 66.
LaTeX Font Info:    Font shape `T1/Merriwthr-OsF/m/n' will be
(Font)              scaled to size 8.0pt on input line 66.
LaTeX Font Info:    Font shape `T1/Merriwthr-OsF/m/it' will be
(Font)              scaled to size 8.0pt on input line 66.
LaTeX Font Info:    Font shape `T1/Merriwthr-OsF/b/it' will be
(Font)              scaled to size 8.0pt on input line 66.
Package caption Info: Begin \AtBeginDocument code.
Package caption Info: End \AtBeginDocument code.
```

```
(c:/texlive/2023/texmf-dist/tex/latex/translations/translations-basic-dictionar
y-english.trsl
File: translations-basic-dictionary-english.trsl (english translation file `tra
nslations-basic-dictionary')
)
Package translations Info: loading dictionary `translations-basic-dictionary' f
or `english'. on input line 66.
TextBlockOrigin set to 4pc+6.64pt x 4pc+6pt
<oup.pdf, id=140, 49.18375pt x 48.18pt>
File: oup.pdf Graphic file (type pdf)
<use oup.pdf>
Package pdftex.def Info: oup.pdf  used on input line 84.
(pdftex.def)             Requested size: 59.24683pt x 58.038pt.
<gigascience-logo.pdf, id=141, 99.37125pt x 33.12375pt>
File: gigascience-logo.pdf Graphic file (type pdf)
<use gigascience-logo.pdf>
Package pdftex.def Info: gigascience-logo.pdf  used on input line 84.
(pdftex.def)             Requested size: 126.00902pt x 42.0pt.

Overfull \hbox (54.64pt too wide) in paragraph at lines 84--84
[][]
 []

LaTeX Font Info:    Font shape `T1/Merriwthr-OsF/m/n' will be
(Font)              scaled to size 14.0pt on input line 84.
LaTeX Font Info:    Font shape `T1/Merriwthr-OsF/m/n' will be
(Font)              scaled to size 8.99997pt on input line 84.
LaTeX Font Info:    Calculating math sizes for size <14> on input line 84.
LaTeX Font Info:    Font shape `T1/Merriwthr-OsF/m/up' will be
(Font)              scaled to size 14.0pt on input line 84.
LaTeX Font Info:    Font shape `T1/Merriwthr-OsF/m/up' will be
(Font)              scaled to size 11.66617pt on input line 84.
LaTeX Font Info:    Font shape `T1/Merriwthr-OsF/m/up' will be
(Font)              scaled to size 9.79996pt on input line 84.
LaTeX Font Info:    External font `cmex10' loaded for size
(Font)              <14> on input line 84.
LaTeX Font Info:    External font `cmex10' loaded for size
(Font)              <11.66617> on input line 84.
LaTeX Font Info:    External font `cmex10' loaded for size
(Font)              <9.79996> on input line 84.
LaTeX Font Info:    Font shape `T1/Merriwthr-OsF/m/n' will be
(Font)              scaled to size 11.66617pt on input line 84.
LaTeX Font Info:    Font shape `T1/Merriwthr-OsF/m/n' will be
(Font)              scaled to size 9.79996pt on input line 84.
LaTeX Font Info:    Font shape `T1/Merriwthr-OsF/m/it' will be
(Font)              scaled to size 14.0pt on input line 84.
LaTeX Font Info:    Font shape `T1/Merriwthr-OsF/m/it' will be
(Font)              scaled to size 11.66617pt on input line 84.
LaTeX Font Info:    Font shape `T1/Merriwthr-OsF/m/it' will be
(Font)              scaled to size 9.79996pt on input line 84.
```

```
LaTeX Font Info:    Font shape `T1/Merriwthr-OsF/b/n' will be
(Font)              scaled to size 18.0pt on input line 84.
LaTeX Font Info:    Font shape `T1/Merriwthr-OsF/m/n' will be
(Font)              scaled to size 13.0pt on input line 84.
LaTeX Font Info:    Calculating math sizes for size <13> on input line
84.
LaTeX Font Info:    Font shape `T1/Merriwthr-OsF/m/up' will be
(Font)              scaled to size 13.0pt on input line 84.
LaTeX Font Info:    Font shape `T1/Merriwthr-OsF/m/up' will be
(Font)              scaled to size 10.83287pt on input line 84.
LaTeX Font Info:    Font shape `T1/Merriwthr-OsF/m/up' will be
(Font)              scaled to size 9.09996pt on input line 84.

LaTeX Font Warning: Font shape `OMS/cmsy/m/n' in size <13> not available
(Font)              size <12> substituted on input line 84.

LaTeX Font Info:    External font `cmex10' loaded for size
(Font)              <13> on input line 84.
LaTeX Font Info:    External font `cmex10' loaded for size
(Font)              <10.83287> on input line 84.
LaTeX Font Info:    External font `cmex10' loaded for size
(Font)              <9.09996> on input line 84.

LaTeX Font Warning: Font shape `OML/cmm/m/it' in size <13> not available
(Font)              size <12> substituted on input line 84.

LaTeX Font Info:    Font shape `T1/Merriwthr-OsF/m/n' will be
(Font)              scaled to size 10.83287pt on input line 84.
LaTeX Font Info:    Font shape `T1/Merriwthr-OsF/m/n' will be
(Font)              scaled to size 9.09996pt on input line 84.
LaTeX Font Info:    Font shape `T1/Merriwthr-OsF/m/it' will be
(Font)              scaled to size 13.0pt on input line 84.
LaTeX Font Info:    Font shape `T1/Merriwthr-OsF/m/it' will be
(Font)              scaled to size 10.83287pt on input line 84.
LaTeX Font Info:    Font shape `T1/Merriwthr-OsF/m/it' will be
(Font)              scaled to size 9.09996pt on input line 84.
LaTeX Font Info:    Trying to load font information for TS1+Merriwthr-OsF
on in
put line 84.
(c:/texlive/2023/texmf-dist/tex/latex/merriweather/TS1Merriwthr-OsF.fd
File: TS1Merriwthr-OsF.fd 2020/08/30 (autoinst) Font definitions for
TS1/Merriw
thr-OsF.
)
LaTeX Font Info:    Font shape `TS1/Merriwthr-OsF/m/n' will be
(Font)              scaled to size 10.83287pt on input line 84.
Package microtype Info: Loading generic protrusion settings for font
family
(microtype)             `Merriwthr-OsF' (encoding: TS1).
(microtype)             For optimal results, create family-specific
settings.
(microtype)             See the microtype manual for details.
LaTeX Font Info:    Font shape `T1/Merriwthr-OsF/m/n' will be
(Font)              scaled to size 9.0pt on input line 84.
```

```
LaTeX Font Info:    Font shape `T1/Merriwthr-OsF/m/up' will be
(Font)              scaled to size 9.0pt on input line 84.
LaTeX Font Info:    Font shape `T1/Merriwthr-OsF/m/up' will be
(Font)              scaled to size 7.0pt on input line 84.
LaTeX Font Info:    Font shape `T1/Merriwthr-OsF/m/up' will be
(Font)              scaled to size 5.0pt on input line 84.
LaTeX Font Info:    External font `cmex10' loaded for size
(Font)              <9> on input line 84.
LaTeX Font Info:    External font `cmex10' loaded for size
(Font)              <7> on input line 84.
LaTeX Font Info:    External font `cmex10' loaded for size
(Font)              <5> on input line 84.
LaTeX Font Info:    Font shape `T1/Merriwthr-OsF/m/n' will be
(Font)              scaled to size 7.0pt on input line 84.
LaTeX Font Info:    Font shape `T1/Merriwthr-OsF/m/n' will be
(Font)              scaled to size 5.0pt on input line 84.
LaTeX Font Info:    Font shape `T1/Merriwthr-OsF/m/it' will be
(Font)              scaled to size 9.0pt on input line 84.
LaTeX Font Info:    Font shape `T1/Merriwthr-OsF/m/it' will be
(Font)              scaled to size 7.0pt on input line 84.
LaTeX Font Info:    Font shape `T1/Merriwthr-OsF/m/it' will be
(Font)              scaled to size 5.0pt on input line 84.
LaTeX Font Info:    Font shape `T1/Merriwthr-OsF/m/n' will be
(Font)              scaled to size 6.5pt on input line 84.
LaTeX Font Info:    Calculating math sizes for size <6.5> on input line
84.
LaTeX Font Info:    Font shape `T1/Merriwthr-OsF/m/up' will be
(Font)              scaled to size 6.5pt on input line 84.
LaTeX Font Info:    Font shape `T1/Merriwthr-OsF/m/up' will be
(Font)              scaled to size 5.41643pt on input line 84.
LaTeX Font Info:    Font shape `T1/Merriwthr-OsF/m/up' will be
(Font)              scaled to size 4.54997pt on input line 84.

LaTeX Font Warning: Font shape `OMS/cmsy/m/n' in size <6.5> not available
(Font)              size <6> substituted on input line 84.


LaTeX Font Warning: Font shape `OMS/cmsy/m/n' in size <5.41643> not
available
(Font)              size <5> substituted on input line 84.


LaTeX Font Warning: Font shape `OMS/cmsy/m/n' in size <4.54997> not
available
(Font)              size <5> substituted on input line 84.

LaTeX Font Info:    External font `cmex10' loaded for size
(Font)              <6.5> on input line 84.
LaTeX Font Info:    External font `cmex10' loaded for size
(Font)              <5.41643> on input line 84.
LaTeX Font Info:    External font `cmex10' loaded for size
(Font)              <4.54997> on input line 84.

LaTeX Font Warning: Font shape `OML/cmm/m/it' in size <6.5> not available
```

```
(Font)              size <6> substituted on input line 84.


LaTeX Font Warning: Font shape `OML/cmm/m/it' in size <5.41643> not
available
(Font)              size <5> substituted on input line 84.


LaTeX Font Warning: Font shape `OML/cmm/m/it' in size <4.54997> not
available
(Font)              size <5> substituted on input line 84.

LaTeX Font Info:    Font shape `T1/Merriwthr-OsF/m/n' will be
(Font)              scaled to size 5.41643pt on input line 84.
LaTeX Font Info:    Font shape `T1/Merriwthr-OsF/m/n' will be
(Font)              scaled to size 4.54997pt on input line 84.
LaTeX Font Info:    Font shape `T1/Merriwthr-OsF/m/it' will be
(Font)              scaled to size 6.5pt on input line 84.
LaTeX Font Info:    Font shape `T1/Merriwthr-OsF/m/it' will be
(Font)              scaled to size 5.41643pt on input line 84.
LaTeX Font Info:    Font shape `T1/Merriwthr-OsF/m/it' will be
(Font)              scaled to size 4.54997pt on input line 84.
LaTeX Font Info:    Font shape `TS1/Merriwthr-OsF/m/n' will be
(Font)              scaled to size 5.41643pt on input line 84.

Overfull \hbox (54.64pt too wide) in paragraph at lines 84--84
[][][]
 []

LaTeX Font Info:    Font shape `T1/Merriwthr-OsF/b/n' will be
(Font)              scaled to size 10.0pt on input line 84.
LaTeX Font Info:    Font shape `T1/Merriwthr-OsF/b/n' will be
(Font)              scaled to size 8.0pt on input line 84.

Overfull \hbox (54.64pt too wide) in paragraph at lines 84--84
[][][]
 []

LaTeX Font Info:    Font shape `T1/Merriwthr-OsF/m/n' will be
(Font)              scaled to size 7.8pt on input line 95.
LaTeX Font Info:    Font shape `T1/Merriwthr-OsF/b/n' will be
(Font)              scaled to size 7.8pt on input line 95.
[1{c:/texlive/2023/texmf-
var/fonts/map/pdftex/updmap/pdftex.map}{c:/texlive/202
3/texmf-
dist/fonts/enc/dvips/merriweather/merriwthr_posqbl.enc}{c:/texlive/2023
/texmf-dist/fonts/enc/dvips/merriweather/merriwthr_owzwzj.enc}



 <./oup.pdf> <./gigascience-logo.pdf>]
LaTeX Font Info:    Font shape `T1/Merriwthr-OsF/m/n' will be
(Font)              scaled to size 10.0pt on input line 97.
LaTeX Font Info:    Font shape `T1/Merriwthr-OsF/m/n' will be
```

```
(Font)              scaled to size 3.75pt on input line 97.
LaTeX Font Info:    Trying to load font information for T1+MerriwthrSans-
OsF on
 input line 97.
(c:/texlive/2023/texmf-dist/tex/latex/merriweather/T1MerriwthrSans-OsF.fd
File: T1MerriwthrSans-OsF.fd 2020/08/30 (autoinst) Font definitions for
T1/Merr
iwthrSans-OsF.
)
LaTeX Font Info:    Font shape `T1/MerriwthrSans-OsF/m/n' will be
(Font)              scaled to size 3.75pt on input line 97.
Package microtype Info: Loading generic protrusion settings for font
family
(microtype)             `MerriwthrSans-OsF' (encoding: T1).
(microtype)             For optimal results, create family-specific
settings.
(microtype)             See the microtype manual for details.
LaTeX Font Info:    Font shape `T1/Merriwthr-OsF/b/n' will be
(Font)              scaled to size 7.5pt on input line 100.

Package natbib Warning: Citation `o2021assignment' on page 2 undefined on
input
 line 100.


Package natbib Warning: Citation `rambaut2020dynamic' on page 2 undefined
on in
put line 100.


Package natbib Warning: Citation `cov-lineages-lineages-website' on page
2 unde
fined on input line 100.


Package natbib Warning: Citation `smith2020integrated' on page 2
undefined on i
nput line 100.


Package natbib Warning: Citation `robishaw2021genomic' on page 2
undefined on i
nput line 100.


Package natbib Warning: Citation `oh2022advancing' on page 2 undefined on
input
 line 100.


Package natbib Warning: Citation `o2021assignment' on page 2 undefined on
input
 line 100.
```

Package natbib Warning: Citation `rambaut2020dynamic' on page 2 undefined on in
put line 100.

Package natbib Warning: Citation `oh2022advancing' on page 2 undefined on input
 line 102.

Package natbib Warning: Citation `shu2017gisaid' on page 2 undefined on input l
ine 102.

Package natbib Warning: Citation `jahn2022early' on page 2 undefined on input l
ine 104.

Package natbib Warning: Citation `smyth2022tracking' on page 2 undefined on inp
ut line 104.

Package natbib Warning: Citation `agrawal2022prevalence' on page 2 undefined on
 input line 104.

Package natbib Warning: Citation `peccia_measurement_2020' on page 2 undefined
on input line 104.

Package natbib Warning: Citation `NEMUDRYI2020100098' on page 2 undefined on in
put line 104.

Package natbib Warning: Citation `hoar2022looking' on page 2 undefined on input
 line 104.

Package natbib Warning: Citation `amman2022national' on page 2 undefined on inp
ut line 104.

Package natbib Warning: Citation `munteanu2023sars' on page 2 undefined on inpu
t line 104.

Package natbib Warning: Citation `gregory2021samrefiner' on page 2 undefined on
 input line 104.

Package natbib Warning: Citation `barbe2022sars' on page 2 undefined on input l
ine 104.

Package natbib Warning: Citation `smyth2022tracking' on page 2 undefined on inp
ut line 104.

Package natbib Warning: Citation `agrawal2022genome' on page 2 undefined on inp
ut line 104.

Package natbib Warning: Citation `NEMUDRYI2020100098' on page 2 undefined on in
put line 104.

Package natbib Warning: Citation `karthikeyan2021wastewater' on page 2 undefine
d on input line 106.

Package natbib Warning: Citation `pechlivanis2022detecting' on page 2 undefined
 on input line 106.

Package natbib Warning: Citation `valieris2022mixture' on page 2 undefined on i
nput line 106.

Package natbib Warning: Citation `ellmen2021alcov' on page 2 undefined on input
 line 106.

Package natbib Warning: Citation `amman2022national' on page 2 undefined on inp
ut line 106.

Package natbib Warning: Citation `barker2021mmmvi' on page 2 undefined on input

```
 line 106.


Package natbib Warning: Citation `schumannsars' on page 2 undefined on
input li
ne 106.


Package natbib Warning: Citation `gregory2021samrefiner' on page 2
undefined on
 input line 106.


Package natbib Warning: Citation `jahn2022early' on page 2 undefined on
input l
ine 106.


Package natbib Warning: Citation `gafurov2022virpool' on page 2 undefined
on in
put line 106.


Package natbib Warning: Citation `posada2021v' on page 2 undefined on
input lin
e 106.


Package natbib Warning: Citation `baaijens2022lineage' on page 2
undefined on i
nput line 106.


Package natbib Warning: Citation `Korobeynikov2022wastewaterSPAdes' on
page 2 u
ndefined on input line 106.


Package natbib Warning: Citation `kayikcioglu2023performance' on page 2
undefin
ed on input line 106.


Package natbib Warning: Citation `kayikcioglu2023performance' on page 2
undefin
ed on input line 110.


Package natbib Warning: Citation `bray2016near' on page 2 undefined on
input li
ne 110.
```

Package natbib Warning: Citation `baaijens2022lineage' on page 2
undefined on i
nput line 110.

Package natbib Warning: Citation `karthikeyan2021wastewater' on page 2
undefine
d on input line 110.

Package natbib Warning: Citation `sutcliffe2023tracking' on page 2
undefined on
 input line 110.

Package natbib Warning: Citation `agrawal2022genome' on page 2 undefined
on inp
ut line 112.

Package natbib Warning: Citation `karthikeyan2021wastewater' on page 2
undefine
d on input line 113.

Package natbib Warning: Citation `turakhia2021ultrafast' on page 2
undefined on
 input line 113.

Package natbib Warning: Citation `baaijens2022lineage' on page 2
undefined on i
nput line 115.

Package natbib Warning: Citation `gafurov2022virpool' on page 2 undefined
on in
put line 115.

Package natbib Warning: Citation `posada2021v' on page 2 undefined on
input lin
e 115.

Package natbib Warning: Citation `bray2016near' on page 2 undefined on
input li
ne 115.

Package natbib Warning: Citation `baaijens2022lineage' on page 2
undefined on i
nput line 115.

```
Package natbib Warning: Citation `kayikcioglu2023performance' on page 2 
undefin
ed on input line 120.


Package natbib Warning: Citation `sutcliffe2023tracking' on page 2 
undefined on
 input line 120.


Package natbib Warning: Citation `munteanu2023sars' on page 2 undefined 
on inpu
t line 120.


Package natbib Warning: Citation `agrawal2022prevalence' on page 2 
undefined on
 input line 120.


Package natbib Warning: Citation `agrawal2022genome' on page 2 undefined 
on inp
ut line 120.


Package natbib Warning: Citation `agrawal2022genome' on page 2 undefined 
on inp
ut line 120.


Package natbib Warning: Citation `bray2016near' on page 2 undefined on 
input li
ne 120.


Package natbib Warning: Citation `baaijens2022lineage' on page 2 
undefined on i
nput line 120.


Package natbib Warning: Citation `di2017nextflow' on page 2 undefined on 
input
line 120.


Package natbib Warning: Citation `VLQ-nf' on page 2 undefined on input 
line 120
.


Package natbib Warning: Citation `bray2016near' on page 2 undefined on 
input li
ne 120.
```

```
Package natbib Warning: Citation `agrawal2022genome' on page 2 undefined
on inp
ut line 120.


Package natbib Warning: Citation `agrawal2022prevalence' on page 2
undefined on
 input line 120.


Package natbib Warning: Citation `agrawal2023comprehensive' on page 2
undefined
 on input line 120.

LaTeX Font Info:    Font shape `T1/Merriwthr-OsF/m/it' will be
(Font)              scaled to size 7.8pt on input line 121.
[2

{c:/texlive/2023/texmf-
dist/fonts/enc/dvips/merriweather/merriwthr_ags7qn.enc}]
<Figure1_overview.pdf, id=181, 1800.35355pt x 1579.44817pt>
File: Figure1_overview.pdf Graphic file (type pdf)
<use Figure1_overview.pdf>
Package pdftex.def Info: Figure1_overview.pdf  used on input line 126.
(pdftex.def)             Requested size: 439.4021pt x 385.48602pt.
LaTeX Font Info:    Font shape `T1/Merriwthr-OsF/m/n' will be
(Font)              scaled to size 6.0pt on input line 127.
LaTeX Font Info:    Font shape `T1/Merriwthr-OsF/b/n' will be
(Font)              scaled to size 6.0pt on input line 127.
LaTeX Font Info:    Font shape `T1/Merriwthr-OsF/m/it' will be
(Font)              scaled to size 6.0pt on input line 127.

Package natbib Warning: Citation `agrawal2022genome' on page 3 undefined
on inp
ut line 127.


Package natbib Warning: Citation `baaijens2022lineage' on page 3
undefined on i
nput line 127.

LaTeX Font Info:    Font shape `T1/Merriwthr-OsF/b/n' will be
(Font)              scaled to size 7.0pt on input line 133.

Package natbib Warning: Citation `kayikcioglu2023performance' on page 3
undefin
ed on input line 133.

LaTeX Font Info:    Font shape `T1/Merriwthr-OsF/b/it' will be
(Font)              scaled to size 7.0pt on input line 138.
```

```
Package natbib Warning: Citation `agrawal2022genome' on page 3 undefined
on inp
ut line 141.

LaTeX Font Info:    Font shape `T1/Merriwthr-OsF/m/n' will be
(Font)              scaled to size 5.00003pt on input line 141.

Package natbib Warning: Citation `karthikeyan2021wastewater' on page 3
undefine
d on input line 142.


Package natbib Warning: Citation `pechlivanis2022detecting' on page 3
undefined
 on input line 143.


Package natbib Warning: Citation `valieris2022mixture' on page 3
undefined on i
nput line 144.


Package natbib Warning: Citation `ellmen2021alcov' on page 3 undefined on
input
 line 145.


Package natbib Warning: Citation `amman2022national' on page 3 undefined
on inp
ut line 146.


Package natbib Warning: Citation `barker2021mmmvi' on page 3 undefined on
input
 line 147.


Package natbib Warning: Citation `schumannsars' on page 3 undefined on
input li
ne 148.


Package natbib Warning: Citation `gregory2021samrefiner' on page 3
undefined on
 input line 149.


Package natbib Warning: Citation `jahn2022early' on page 3 undefined on
input l
ine 150.


Package natbib Warning: Citation `Korobeynikov2022wastewaterSPAdes' on
page 3 u
```

```
ndefined on input line 151.


Package natbib Warning: Citation `baaijens2022lineage' on page 3 
undefined on i
nput line 160.


Package natbib Warning: Citation `gafurov2022virpool' on page 3 undefined 
on in
put line 161.


Package natbib Warning: Citation `posada2021v' on page 3 undefined on 
input lin
e 162.


Package natbib Warning: Citation `kayikcioglu2023performance' on page 3 
undefin
ed on input line 168.


Overfull \hbox (1.13507pt too wide) in paragraph at lines 136--171
[][]
 []


Package natbib Warning: Citation `agrawal2022prevalence' on page 3 
undefined on
 input line 180.


Package natbib Warning: Citation `agrawal2022genome' on page 3 undefined 
on inp
ut line 180.


Package natbib Warning: Citation `agrawal2022prevalence' on page 3 
undefined on
 input line 180.


Package natbib Warning: Citation `agrawal2022genome' on page 3 undefined 
on inp
ut line 180.


Package natbib Warning: Citation `agrawal2022genome' on page 3 undefined 
on inp
ut line 180.
```

```
Package natbib Warning: Citation `agrawal2022prevalence' on page 3
undefined on
 input line 180.


Package natbib Warning: Citation `agrawal2023comprehensive' on page 3
undefined
 on input line 180.

LaTeX Font Info:    Font shape `T1/Merriwthr-OsF/m/up' will be
(Font)              scaled to size 6.0pt on input line 208.
LaTeX Font Info:    External font `cmex10' loaded for size
(Font)              <6> on input line 208.

LaTeX Warning: Text page 3 contains only floats.


Overfull \vbox (1.80305pt too high) has occurred while \output is active
[]

[3] [4 <./Figure1_overview.pdf>]
LaTeX Font Info:    Font shape `T1/Merriwthr-OsF/b/n' will be
(Font)              scaled to size 8.5pt on input line 231.
LaTeX Font Info:    Font shape `T1/Merriwthr-OsF/b/it' will be
(Font)              scaled to size 8.5pt on input line 231.
LaTeX Font Info:    Font shape `T1/Merriwthr-OsF/m/n' will be
(Font)              scaled to size 8.5pt on input line 231.
<Figure2_standards.png, id=238, 604.21346pt x 456.50172pt>
File: Figure2_standards.png Graphic file (type png)
<use Figure2_standards.png>
Package pdftex.def Info: Figure2_standards.png  used on input line 246.
(pdftex.def)             Requested size: 483.34827pt x 365.1949pt.

Package natbib Warning: Citation `agrawal2022prevalence' on page 5
undefined on
 input line 253.


Package natbib Warning: Citation `gangavarapu2022outbreak' on page 5
undefined
on input line 255.


Package natbib Warning: Citation `Outbreak-website' on page 5 undefined
on inpu
t line 255.


Package natbib Warning: Citation `hadfield2018nextstrain' on page 5
undefined o
n input line 255.
```

```
Package natbib Warning: Citation `Nextstrain-website' on page 5 undefined
on in
put line 255.


Package natbib Warning: Citation `oh2022advancing' on page 5 undefined on
input
 line 255.


Package natbib Warning: Citation `agrawal2022prevalence' on page 5
undefined on
 input line 255.

<Figure3_paneuger.pdf, id=244, 722.7pt x 1084.05pt>
File: Figure3_paneuger.pdf Graphic file (type pdf)
<use Figure3_paneuger.pdf>
Package pdftex.def Info: Figure3_paneuger.pdf  used on input line 265.
(pdftex.def)             Requested size: 390.58379pt x 585.87453pt.

Package natbib Warning: Citation `agrawal2022genome' on page 5 undefined
on inp
ut line 270.


Package natbib Warning: Citation `gangavarapu2022outbreak' on page 5
undefined
on input line 272.


Package natbib Warning: Citation `Outbreak-website' on page 5 undefined
on inpu
t line 272.


Package natbib Warning: Citation `hadfield2018nextstrain' on page 5
undefined o
n input line 272.


Package natbib Warning: Citation `Nextstrain-website' on page 5 undefined
on in
put line 272.

[5] [6 <./Figure2_standards.png>] [7 <./Figure3_paneuger.pdf>]
<Figure4_ffm-airport.pdf, id=272, 1411.52344pt x 699.36281pt>
File: Figure4_ffm-airport.pdf Graphic file (type pdf)
<use Figure4_ffm-airport.pdf>
Package pdftex.def Info: Figure4_ffm-airport.pdf  used on input line 283.
(pdftex.def)             Requested size: 463.81499pt x 229.79799pt.

Package natbib Warning: Citation `agrawal2022genome' on page 8 undefined
on inp
ut line 284.
```

```
Underfull \vbox (badness 1721) has occurred while \output is active []

LaTeX Font Info:    Font shape `T1/Merriwthr-OsF/b/sl' in size <7.5> not
availa
ble
(Font)              Font shape `T1/Merriwthr-OsF/b/it' tried instead on
input l
ine 292.
LaTeX Font Info:    Font shape `T1/Merriwthr-OsF/b/it' will be
(Font)              scaled to size 7.5pt on input line 292.
[8 <./Figure4_ffm-airport.pdf>]
<Figure5_aaf-standards.pdf, id=301, 1084.05pt x 1084.05pt>
File: Figure5_aaf-standards.pdf Graphic file (type pdf)
<use Figure5_aaf-standards.pdf>
Package pdftex.def Info: Figure5_aaf-standards.pdf  used on input line
307.
(pdftex.def)             Requested size: 488.22787pt x 488.23152pt.

Package natbib Warning: Citation `rambaut2020dynamic' on page 9 undefined
on in
put line 319.


Package natbib Warning: Citation `bray2016near' on page 9 undefined on
input li
ne 321.


Package natbib Warning: Citation `baaijens2022lineage' on page 9
undefined on i
nput line 321.


Package natbib Warning: Citation `baaijens2022lineage' on page 9
undefined on i
nput line 326.


Package natbib Warning: Citation `karthikeyan2021wastewater' on page 9
undefine
d on input line 326.


Package natbib Warning: Citation `turakhia2021ultrafast' on page 9
undefined on
 input line 326.

[9] [10 <./Figure5_aaf-standards.pdf>]

Package natbib Warning: Citation `agrawal2022genome' on page 11 undefined
on in
put line 354.
```

Package natbib Warning: Citation `agrawal2022prevalence' on page 11
undefined o
n input line 354.

Package natbib Warning: Citation `agrawal2023comprehensive' on page 11
undefine
d on input line 354.

Package natbib Warning: Citation `karthikeyan2021wastewater' on page 11
undefin
ed on input line 356.

Package natbib Warning: Citation `pechlivanis2022detecting' on page 11
undefine
d on input line 356.

Package natbib Warning: Citation `valieris2022mixture' on page 11
undefined on
input line 356.

Package natbib Warning: Citation `ellmen2021alcov' on page 11 undefined
on inpu
t line 356.

Package natbib Warning: Citation `amman2022national' on page 11 undefined
on in
put line 356.

Package natbib Warning: Citation `barker2021mmmvi' on page 11 undefined
on inpu
t line 356.

Package natbib Warning: Citation `schumannsars' on page 11 undefined on
input l
ine 356.

Package natbib Warning: Citation `gregory2021samrefiner' on page 11
undefined o
n input line 356.

Package natbib Warning: Citation `jahn2022early' on page 11 undefined on
input

line 356.

Package natbib Warning: Citation `gafurov2022virpool' on page 11
undefined on i
nput line 356.

Package natbib Warning: Citation `posada2021v' on page 11 undefined on
input li
ne 356.

Package natbib Warning: Citation `baaijens2022lineage' on page 11
undefined on
input line 356.

Package natbib Warning: Citation `Korobeynikov2022wastewaterSPAdes' on
page 11
undefined on input line 356.

Package natbib Warning: Citation `kayikcioglu2023performance' on page 11
undefi
ned on input line 356.

Package natbib Warning: Citation `karthikeyan2021wastewater' on page 11
undefin
ed on input line 356.

Package natbib Warning: Citation `baaijens2022lineage' on page 11
undefined on
input line 356.

Package natbib Warning: Citation `valieris2022mixture' on page 11
undefined on
input line 356.

Package natbib Warning: Citation `amman2022national' on page 11 undefined
on in
put line 356.

Package natbib Warning: Citation `karthikeyan2021wastewater' on page 11
undefin
ed on input line 356.

Package natbib Warning: Citation `gafurov2022virpool' on page 11 undefined on i
nput line 356.

Package natbib Warning: Citation `schumannsars' on page 11 undefined on input l
ine 356.

Package natbib Warning: Citation `sutcliffe2023tracking' on page 11 undefined o
n input line 356.

Package natbib Warning: Citation `munteanu2024rigorous' on page 11 undefined on
 input line 356.

Package natbib Warning: Citation `hoar2022looking' on page 11 undefined on inpu
t line 365.

Package natbib Warning: Citation `mcbroome2024framework' on page 11 undefined o
n input line 368.

Package natbib Warning: Citation `karthikeyan2021wastewater' on page 11 undefin
ed on input line 370.

Package natbib Warning: Citation `smyth2022tracking' on page 11 undefined on in
put line 370.

Package natbib Warning: Citation `abdeldayem2022viral' on page 11 undefined on
input line 370.

Package natbib Warning: Citation `zhuang2024early' on page 11 undefined on inpu
t line 370.

Package natbib Warning: Citation `ellmen2024learning' on page 11 undefined on i
nput line 370.

```
LaTeX Font Info:    Font shape `TS1/Merriwthr-OsF/m/n' will be
(Font)              scaled to size 7.5pt on input line 381.
[11]

Package natbib Warning: Citation `agrawal2023comprehensive' on page 12
undefine
d on input line 394.


Package natbib Warning: Citation `cingolani2012program' on page 12
undefined on
 input line 395.


Package natbib Warning: Citation `shu2017gisaid' on page 12 undefined on
input
line 395.


Package natbib Warning: Citation `MAMUSS' on page 12 undefined on input
line 39
5.


Package natbib Warning: Citation `baaijens2022lineage' on page 12
undefined on
input line 398.


Package natbib Warning: Citation `bray2016near' on page 12 undefined on
input l
ine 398.


Package natbib Warning: Citation `shu2017gisaid' on page 12 undefined on
input
line 398.


Package natbib Warning: Citation `rambaut2020dynamic' on page 12
undefined on i
nput line 398.


Package natbib Warning: Citation `o2021assignment' on page 12 undefined
on inpu
t line 398.


Package natbib Warning: Citation `baaijens2022lineage' on page 12
undefined on
input line 400.
```

```
Package natbib Warning: Citation `VLQ' on page 12 undefined on input line
400.


Package natbib Warning: Citation `di2017nextflow' on page 12 undefined on
input
 line 400.


Package natbib Warning: Citation `VLQ-nf' on page 12 undefined on input
line 40
0.


Package natbib Warning: Citation `gangavarapu2022outbreak' on page 12
undefined
 on input line 408.

[12]

Package natbib Warning: Citation `gangavarapu2022outbreak' on page 13
undefined
 on input line 415.


Package natbib Warning: Citation `baaijens2022lineage' on page 13
undefined on
input line 435.


Package natbib Warning: Citation `baaijens2022lineage' on page 13
undefined on
input line 453.

LaTeX Font Info:    Trying to load font information for T1+lmtt on input
line 4
57.
(c:/texlive/2023/texmf-dist/tex/latex/lm/t1lmtt.fd
File: t1lmtt.fd 2015/05/01 v1.6.1 Font defs for Latin Modern
)
Package microtype Info: Loading generic protrusion settings for font
family
(microtype)             `lmtt' (encoding: T1).
(microtype)             For optimal results, create family-specific
settings.
(microtype)             See the microtype manual for details.
LaTeX Font Info:    Font shape `T1/Merriwthr-OsF/m/up' will be
(Font)              scaled to size 7.5pt on input line 457.

Package natbib Warning: Citation `https://doi.org/10.5524/102547' on page
13 un
defined on input line 480.

[13{c:/texlive/2023/texmf-dist/fonts/enc/dvips/lm/lm-ec.enc}]
```

```
Package natbib Warning: Citation `agrawal2022genome' on page 14 undefined
on in
put line 498.


Package natbib Warning: Citation `agrawal2022prevalence' on page 14
undefined o
n input line 500.


Package natbib Warning: Citation `baaijens2022lineage' on page 14
undefined on
input line 502.

No file main.bbl.
[14

] (./supplement.tex

LaTeX Warning: File `FigureS1_Supplement.png' not found on input line 16.


! Package pdftex.def Error: File `FigureS1_Supplement.png' not found:
using dra
ft setting.

See the pdftex.def package documentation for explanation.
Type  H <return>  for immediate help.
 ...

l.16 ...th=.95\textwidth]{FigureS1_Supplement.png}

Try typing  <return>  to proceed.
If that doesn't work, type  X <return>  to quit.


LaTeX Warning: File `FigureS2_Supplement.png' not found on input line 24.


! Package pdftex.def Error: File `FigureS2_Supplement.png' not found:
using dra
ft setting.

See the pdftex.def package documentation for explanation.
Type  H <return>  for immediate help.
 ...

l.24 ...th=.95\textwidth]{FigureS2_Supplement.png}

Try typing  <return>  to proceed.
If that doesn't work, type  X <return>  to quit.
```

```
Underfull \hbox (badness 1515) in paragraph at lines 39--40
[]\T1/Merriwthr-OsF/m/up/7.5 (+20) Overall, we found the per-for-mance of
the \
T1/Merriwthr-OsF/m/it/7.5 (+20) sequence-based
 []


LaTeX Warning: File `FigureS3_Supplement.pdf' not found on input line 67.


! Package pdftex.def Error: File `FigureS3_Supplement.pdf' not found:
using dra
ft setting.

See the pdftex.def package documentation for explanation.
Type  H <return>  for immediate help.
 ...

l.67 ...th=.95\textwidth]{FigureS3_Supplement.pdf}

Try typing  <return>  to proceed.
If that doesn't work, type  X <return>  to quit.


LaTeX Warning: File `FigureS4_Supplement.png' not found on input line 74.


! Package pdftex.def Error: File `FigureS4_Supplement.png' not found:
using dra
ft setting.

See the pdftex.def package documentation for explanation.
Type  H <return>  for immediate help.
 ...

l.74 ...th=.85\textwidth]{FigureS4_Supplement.png}

Try typing  <return>  to proceed.
If that doesn't work, type  X <return>  to quit.


LaTeX Warning: File `FigureS5_Supplement.png' not found on input line 81.


! Package pdftex.def Error: File `FigureS5_Supplement.png' not found:
using dra
ft setting.

See the pdftex.def package documentation for explanation.
Type  H <return>  for immediate help.
 ...

l.81 ...th=.95\textwidth]{FigureS5_Supplement.png}
```

```
Try typing  <return>  to proceed.
If that doesn't work, type  X <return>  to quit.


Package natbib Warning: Citation `karthikeyan2021wastewater' on page 15
undefin
ed on input line 82.


LaTeX Warning: File `FigureS6_Supplement.png' not found on input line 88.


! Package pdftex.def Error: File `FigureS6_Supplement.png' not found:
using dra
ft setting.

See the pdftex.def package documentation for explanation.
Type  H <return>  for immediate help.
 ...

l.88 ...th=.95\textwidth]{FigureS6_Supplement.png}

Try typing  <return>  to proceed.
If that doesn't work, type  X <return>  to quit.


Package natbib Warning: Citation `karthikeyan2021wastewater' on page 15
undefin
ed on input line 89.

LaTeX Font Info:    Font shape `T1/Merriwthr-OsF/b/n' will be
(Font)              scaled to size 5.0pt on input line 98.
LaTeX Font Info:    Font shape `T1/Merriwthr-OsF/b/it' will be
(Font)              scaled to size 5.0pt on input line 98.

Overfull \hbox (15.11462pt too wide) in paragraph at lines 96--116
[][]
 []

)

Package natbib Warning: There were undefined citations.

[15


] [16] [17] [18] [19] [20] [21] [22


]
enddocument/afterlastpage: lastpage setting LastPage.
(./main.aux)
 ***********
LaTeX2e <2023-11-01> patch level 1
```

```
L3 programming layer <2020/03/25>
 ***********

LaTeX Font Warning: Size substitutions with differences
(Font)              up to 1.0pt have occurred.


LaTeX Font Warning: Some font shapes were not available, defaults
substituted.

Package rerunfilecheck Info: File `main.out' has not changed.
(rerunfilecheck)             Checksum:
7F7C0CFE194ADD1058EA131B78EE3B9B;10187.
 )
Here is how much of TeX's memory you used:
 35354 strings out of 474121
 723441 string characters out of 5747949
 1995190 words of memory out of 5000000
 56071 multiletter control sequences out of 15000+600000
 2053340 words of font info for 791 fonts, out of 8000000 for 9000
 1141 hyphenation exceptions out of 8191
 123i,20n,131p,2660b,1169s stack positions out of
10000i,1000n,20000p,200000b,200000s
<c:/texlive/2023/texmf-dist/fonts/type1/sorkin/merriweather/Merriwthr-
Bold.pf
b><c:/texlive/2023/texmf-dist/fonts/type1/sorkin/merriweather/Merriwthr-
BoldIta
lic.pfb><c:/texlive/2023/texmf-
dist/fonts/type1/sorkin/merriweather/Merriwthr-I
talic.pfb><c:/texlive/2023/texmf-
dist/fonts/type1/sorkin/merriweather/Merriwthr
-Regular.pfb><c:/texlive/2023/texmf-
dist/fonts/type1/sorkin/merriweather/Merriw
thrSans-Regular.pfb><c:/texlive/2023/texmf-
dist/fonts/type1/public/lm/lmtt8.pfb
>
Output written on main.pdf (22 pages, 2343829 bytes).
PDF statistics:
 7057 PDF objects out of 7423 (max. 8388607)
 4767 compressed objects within 48 object streams
 73 named destinations out of 1000 (max. 500000)
 234296 words of extra memory for PDF output out of 266212 (max.
10000000)
```

Manuscript Click here to access/download;Manuscript;manuscript (4).pdf

GigaScience, 2017, 1–24

doi: xx.xxxx/xxxx
Manuscript in Preparation
Research

RESEARCH

Impact of reference design on estimating SARS-CoV-2 lineage abundances from wastewater sequencing data

Eva Aßmann[1,2,†], Shelesh Agrawal[3,†], Laura Orschler[3], Sindy Böttcher[4], Susanne Lackner[3] and Martin Hölzer[1,*]

[1]Genome Competence Center (MF1), Robert Koch Institute, Berlin, Germany and [2]Center for Artificial Intelligence in Public Health Research (ZKI-PH), Robert Koch Institute, Berlin, Germany and [3]Chair of Water and Environmental Biotechnology, Institute IWAR, Department of Civil and Environmental Engineering Sciences, Technical University of Darmstadt, Darmstadt, Germany and [4]Gastroenteritis and Hepatitis Pathogens and Enteroviruses, Robert Koch Institute, Berlin, Germany

*HoelzerM@rki.de
†Contributed equally.

Abstract

Background Sequencing of SARS-CoV-2 RNA from wastewater samples has emerged as a valuable tool for detecting the presence and relative abundances of SARS-CoV-2 variants in a community. By analyzing the viral genetic material present in wastewater, researchers and public health authorities can gain early insights into the spread of virus lineages and emerging mutations. Constructing reference datasets from known SARS-CoV-2 lineages and their mutation profiles has become state-of-the-art for assigning viral lineages and their relative abundances from wastewater sequencing data. However, selecting reference sequences or mutations directly affect the predictive power. **Results** Here, we show the impact of a *mutation*- and *sequence-based* reference reconstruction for SARS-CoV-2 abundance estimation. We benchmark three data sets: 1) synthetic "spike-in" mixtures, 2) German wastewater samples from early 2021, mainly comprising Alpha, and 3) samples obtained from wastewater at an international airport in Germany from the end of 2021, including first signals of Omicron. The two approaches differ in sub-lineage detection, with the marker-*mutation-based* method, in particular, being challenged by the increasing number of mutations and lineages. However, the estimations of both approaches depend on selecting representative references and optimized parameter settings. By performing parameter escalation experiments, we demonstrate the effects of reference size and alternative allele frequency cutoffs for abundance estimation. We show how different parameter settings can lead to different results for our test data sets, and illustrate the effects of virus lineage composition of wastewater samples and references. **Conclusions** Our study highlights current computational challenges, focusing on the general reference design, which directly impacts abundance allocations. We illustrate advantages and disadvantages that may be relevant for further developments in the wastewater community and in the context of defining robust quality metrics.

Key words: SARS-CoV-2; wastewater; sewage; abundance estimation, next-generation sequencing, benchmark

Compiled on: July 2, 2024.
Draft manuscript prepared by the author.

Background

Coronavirus disease 2019 (COVID-19), the highly contagious viral illness caused by severe acute respiratory syndrome coronavirus 2 (SARS-CoV-2), is the most consequential global health crisis since the era of the influenza pandemic of 1918. Since its discovery, SARS-CoV-2 has caused > 775 million confirmed cases of COVID-19 [1] and currently > 4,200 SARS-CoV-2 lineages are defined by the *Pango* network [2, 3, 4]. Genome sequencing has played a central role during the COVID-19 pandemic and beyond in supporting public health agencies, monitoring emerging mutations in the SARS-CoV-2 genome, and advancing precision vaccinology and optimizing molecular tests [5, 6, 7]. Massive sequencing of clinical samples has made monitoring of emerging virus variants possible while emphasizing temporal and spatial variation. With ongoing transmission, further mutations occur in the genome that are part of the viral evolutionary process and result in unique fingerprints. These fingerprints, along with other metrics such as the number of samples with the same mutation profile and their geographic occurrence, are used to label SARS-CoV-2 variants, such as through the nomenclature system proposed and maintained by the Pangolin network and tool [2, 3]. These definitions of virus variants and lineages and the associated mutation profiles can then be used to search for and estimate the proportion of SARS-CoV-2 lineages in mixed samples, e.g. wastewater.

Sequencing capacity, however, is limited, cannot be sustained over the long term for so many clinical samples, and only allows extrapolation based on a relatively small fraction of all infections occurring during the pandemic. In addition, with decreasing incidence numbers, sampling and sequencing efforts are decreasing, raising the need for representative, medium-scale, and sustainable surveillance systems [7] or other approaches. From January 1, 2020 until April 19, 2023, 931,260 genome sequences of COVID-19-positive clinical samples from Germany have been uploaded to the international GISAID platform [8], representing a proportion of 2.426 % out of a total of 38,388,247 reported SARS-CoV-2 cases in Germany [9]. In Germany and other countries, complete detection and sequencing of all positive cases were impossible due to the high infection numbers. However, wastewater-based epidemiology (WBE) has shown the potential to get a much broader snapshot of the SARS-CoV-2 variant circulation at a community level [10, 11, 12, 13, 14, 15]. Integrating genome sequencing with WBE can provide information on circulating SARS-CoV-2 variants in a region [16, 17]. The sequencing methods commonly used in WBE are similar to the ones used for clinical samples, using a general strategy that employs the sequencing of the whole genome via amplification of small, specific regions of the SARS-CoV-2 genome, i.e., targeted sequencing of amplicons via pre-defined primer sequences [18, 19, 11, 20, 14]. Targeted sequencing can achieve a high degree of coverage of informative regions of the genome and, most importantly, reveal to some extent which polymorphisms are linked, making it possible to track SARS-CoV-2 variants of concern (VOCs) and other virus variants.

A particular challenge in performing sequencing and analysis of SARS-CoV-2 from wastewater samples concerns the viral RNA present in many individual fragments rather than complete viral genomes. In addition, these fragments come from the excretions of many infected individuals, making it challenging, if not impossible, to reconstruct individual genomes using bioinformatic approaches like the ones developed for clinical samples of individual patients. Thus, the degradation and fragmentation of SARS-CoV-2 RNA, combined with the presence of multiple virus variants in wastewater samples, make it challenging to reconstruct reliable, complete consensus genomes, often resulting in sequences that represent either a mixture of lineages or predominantly the most abundant variant. In need of computational approaches to analyze mixed wastewater samples, several groups developed similar tools for quality control, sequencing data analysis, and SARS-CoV-2 lineage abundance estimation instead of reconstructing a single consensus genome [21, 22, 23, 24, 16, 25, 26, 18, 10, 27, 28, 29, 30, 31], see Table 1.

Most approaches focus on detecting pre-defined characteristic marker mutations in the sequenced reads and utilize this information for abundance estimation. Common to all these tools is that they require a reference set of either signature marker mutations (hereafter called *mutation-based*) or complete genome sequences (hereafter called *sequence-based*) from which characteristic mutation profiles or kmers (short subsequences of length k) are derived.

Kayikcioglu *et al.* compared the performance of five selected approaches for SARS-CoV-2 lineage abundance estimation on simulated and publicly available mixed population samples [31]. They found that Kallisto [32], as first suggested by Baaijens, Zulli, and Ott *et al.* [29], followed by Freyja [21], achieved most accurate estimations. Sutcliffe *et al.* compared nine computational tools using simulated genomic data in another recent study. Among other things, they tested how the background noise of a previously unknown lineage affects quantification, finding a weak but significant effect on the estimate of the frequency of known lineages that are part of the reference [33].

In a *mutation-based* approach, to estimate the proportion of specific SARS-CoV-2 variants present in a mixed sample, mutations or combinations of mutations characteristic or unique for these variants based on clinical samples can be compared with the mutations detectable in the sample. In principle, and as implemented in a previously used approach [20] (which we refer to here as MAMUSS, Table 1), the occurrence of mutations can be represented by the value of the relative abundance of a VOC or other viral variant. First, the frequency of occurrence of each mutation is calculated from the multiplication of the reads and the allele frequency. The relative abundance describes the percentage ratio of the sum of the read abundance of the characteristic mutations of a SARS-CoV-2 virus variant and the sum of the read abundance of all mutations found in a sample. Accordingly, only the previously selected virus variants and signature mutations that form the reference set are evaluated and others that may occur in the sample are ignored. Another prominent *mutation-based* approach is implemented in the tool Freyja [21]. Freyja solves the de-mixing problem to recover relative lineage abundances from mixed SARS-CoV-2 samples using lineage-determining mutational "barcodes" derived from the UShER global phylogenetic tree [34]. Using mutation abundances and sequencing depth measurements at each position in the genome, Freyja estimates the abundance of lineages in the sample.

As a different methodological approach to reconstruct a reference, the full genome sequence information can be used to automatically select appropriate features (e.g., signature mutations, kmers) and to use them to evaluate the proportions of SARS-CoV-2 variants in wastewater samples instead of a preselected set of marker mutations (*sequence-based*) [29, 27, 28] (Table 1). Again, information derived from sequencing of clinical samples and their lineage annotation are used to generate a representative reference data set that can be then searched via established (pseudo)-alignment methods such as Kallisto [32] as suggested by Baaijens, Zulli, and Ott *et al.* in their VQL tool [29].

In this study, we specifically investigated the effects of reference design and composition on the assignment of relative abundances of SARS-CoV-2 lineages from wastewater sequencing data. As mentioned, various tools have been developed dur-

ing the pandemic (Table 1), and they all have different facets in calculating relative abundances [31, 33, 17]. Here, we tested MAMUSS as a *mutation-based* reference representative and VLQ-nf as a *sequence-based* reference representative on three data sets: 1) a synthetic scenario of "spike-in" mixture samples, 2) samples from Germany from a European wastewater study from early 2021, mainly comprising the VOC Alpha [12], and 3) a sample obtained from wastewater sequencing at the international airport in Frankfurt am Main, Germany from the end of 2021, including first signals of the VOC Omicron [20]. The two approaches for lineage abundance estimation (*mutation-based*/ *sequence-based*) are mainly distinguished by the input data set used for the reference set design and subsequent lineage assignment (Figure 1). Here, we compare exemplary implementations of both general approaches. MAMUSS, as previously applied in [20], implements a representative basic workflow for the *mutation-based* approach focusing on unique marker mutations. For the *sequence-based* approach, we use pseudo-alignments via Kallisto [32] as proposed initially by [29] and their VLQ tool. Based on their idea and scripts, we implemented a slightly modified version of VLQ in a Nextflow [35] pipeline that we call VLQ-nf [36]. We chose VLQ for our *sequence-based* method because it relies on Kallisto as an established tool for quantifying transcripts [32]. A major benefit of implementing the representative methods was the complete control over code, parameters, and inputs, which allowed us to understand better, compare, and interpret the results of our benchmark study and the effects on the reference design.For all three data sets, we deliberately selected data from one sequencing technology, Ion Torrent, to demonstrate reference design and *mutation/sequence-based* effects in a specific, controlled context with which we have much experience [20, 12, 37]. However, it must be noted as a limitation of our study that we are only investigating one sequencing technology.

We show that both the *mutation-based* and *sequence-based* approach can reflect the proportions of SARS-CoV-2 lineages in the different samples but also comprise differences in resolution and the detection of similar sub-lineages depending on the reference set. Both approaches also show advantages and disadvantages in selecting signature marker mutations and genome sequences, respectively. For the *mutation-based* approach as implemented in MAMUSS, it became more and more challenging to select (sub-)lineage-defining marker mutations that provide robust assignments in the context of the increasing diversity and convergent evolution of SARS-CoV-2 lineages.

Table 1. Collection of tools available for sequencing data analysis in WBE and SARS-CoV-2 lineage proportion estimation. We distinguish the tools roughly based on their approach to define a reference set into those using predefined marker mutations and those relying on full genome sequences or both. The two implementations we selected for reference construction and our comparison are indicated in bold. Please note that C-WAP [31] wraps multiple approaches while also including a new *mutation-based* tool, LINDEC.

Tool	Citation	Code
mutation-based		
**MAMUSS**	[20]	github.com/lifehashopes/MAMUSS
Freyja	[21]	github.com/andersen-lab/Freyja
Lineagespot	[22]	github.com/nikopech/lineagespot
LCS	[23]	github.com/rvalieris/LCS
Alcov	[24]	github.com/Ellmen/alcov
VaQuERo	[16]	github.com/fabou-uobaf/VaQuERo
MMMVI	[25]	github.com/dorbarker/voc-identify
PiGx	[26]	github.com/BIMSBbioinfo/pigx_sars-cov-2
SAMRefiner	[18]	github.com/degregory/SAM_Refiner
COJAC	[10]	github.com/cbg-ethz/cojac
wastewaterSPAdes	[30]	–
gromstole	–	github.com/PoonLab/gromstole
CovMix	–	github.com/chrisquince/covmix
sequence-based		
**VLQ-nf**	this study	github.com/rki-mf1/VLQ-nf
VLQ	[29]	github.com/baymlab/wastewater_analysis
VirPool	[27]	github.com/fmfi-compbio/virpool
V-pipe SC2	[28]	cbg-ethz.github.io/V-pipe/sars-cov-2
mutation-based & sequence-based		
C-WAP	[31]	github.com/CFSAN-Biostatistics/C-WAP

Data Description

We selected three wastewater data sets for our comparison to cover 1) a synthetic scenario of "spike-in" mixture samples (*Standards*; n=16 samples), 2) actual wastewater samples from early 2021 from a large European study and collected in Germany [12], mainly comprising the VOC Alpha (*Pan-EU-GER*; n=7 samples), and 3) one sample from the end of 2021 including first signals of the VOC Omicron obtained from wastewater at the international airport in Frankfurt am Main, Germany (*FFM-Airport*; n=1 sample) [20]. The *Standards* comprise RNA from 10 SARS-CoV-2 variants (including the original Wuhan-Hu-1 A.1 lineage), which were mixed in different proportions to generate 16 samples for library preparation and sequencing via Ion Torrent (Table 2). The sequencing data for the *Standards* benchmark are available under the NCBI BioProject number PRJNA912560. Please note that no real wastewater was used to construct the *Standards* (see Methods). The Pan-EU WBE study produced high-quality sequencing data for SARS-CoV-2 wastewater samples across 20 European countries, including 54 municipalities and is available under the NCBI BioProject number PRJNA736964[12]. We selected the seven German samples from this study (SRX11122519 and SRX11122521–SRX11122526; *Pan-EU-GER*) for our benchmark, which were sampled in March 2021 and mainly cover the rise of the VOC Alpha during that time. Lastly, we obtained one sample (SRR17258654; NCBI BioProject number PRJNA789814) from wastewater sampling in November 2021 at the international airport in Frankfurt am Main (*FFM-Airport*), where we found first signals and low proportions of the VOC Omicron arriving during that time in Germany [20]. Note that we deliberately selected Ion Torrent as sequencing technology to harmonize between the selected data sets and to focus on the reference design and *mutation/sequence-based* effects in a specific, controlled context with which we have much experience [20, 12, 37] (see also "Potential implications" section).

Analyses

Both the *mutation-based* and *sequence-based* approaches yield similar SARS-CoV-2 lineage proportions for mixed *Standard* samples but differ on sub-lineage level

We analyzed our *Standards* data set (Table 2) using the *sequence-based* approach implemented in VLQ-nf and an implementation of a *mutation-based* approach, MAMUSS (Table 1). Given ground truth knowledge, we assessed the qualitative and quantitative performance of both methods yielding controlled insights into the strengths and limitations of each approach. When comparing the results with the actual sample composi-

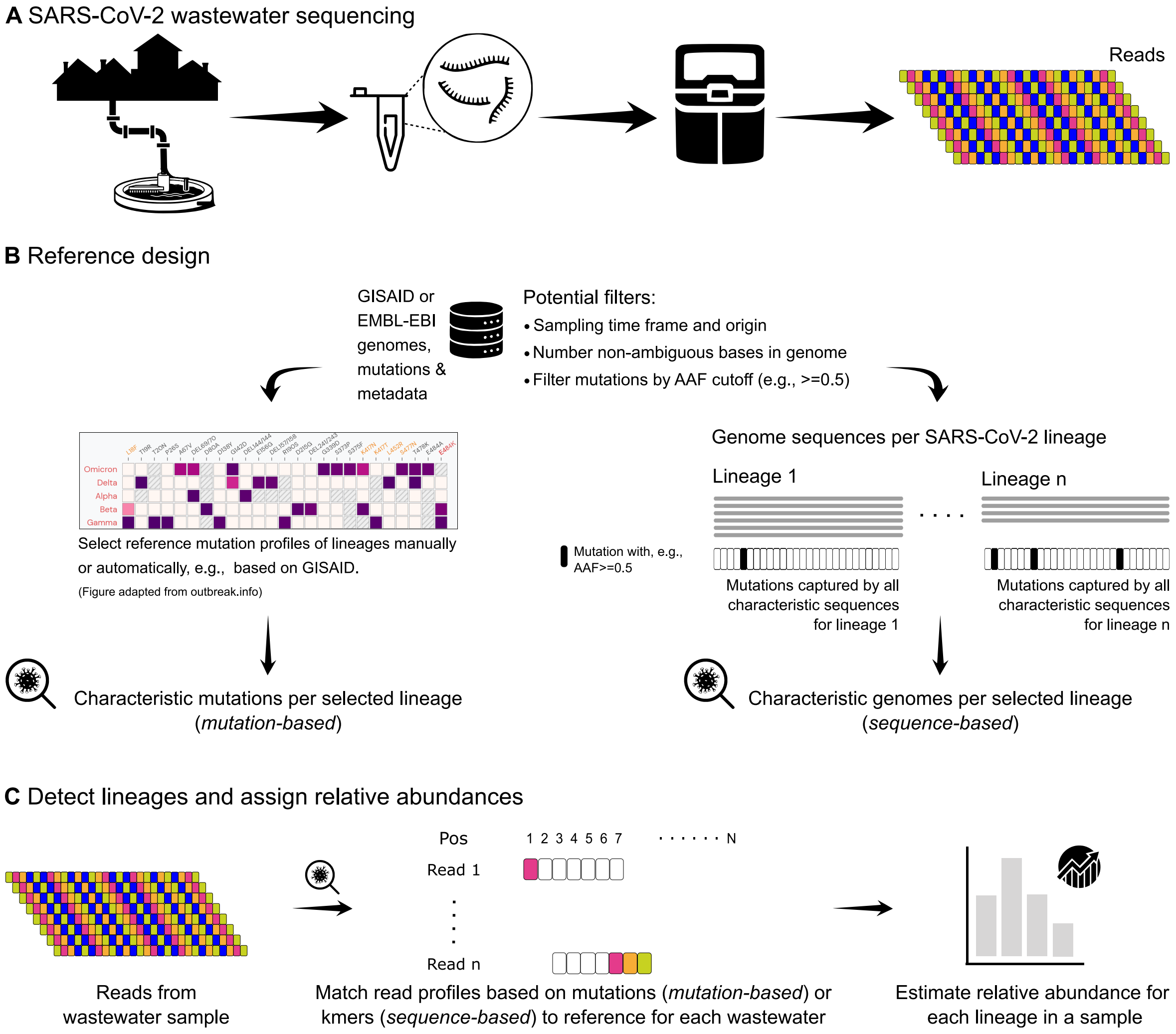

Figure 1. Schematic overview of reference design and lineage abundance estimation from SARS-CoV-2 wastewater sequencing data. (**A**) Wastewater samples are collected from sewer influent, for example. RNA is extracted and, in the context of SARS-CoV-2, usually amplified as cDNA using established primer schemes and then sequenced to obtain short snippets of viral RNA (*reads*). (**B**) Current methods (Table 1 for lineage assignment and abundance estimation need a reference data set, usually constructed from genomes and mutations derived from clinical sequencing and patient samples. Here, we distinguish two general approaches to design the reference, where either marker mutations are pre-selected (*mutation-based*) or full-genome sequences are selected (*sequence-based*). (**C**) The data analysis part may differ considerably depending on the implementation. However, all tools attempt to assign known lineages and estimate their frequency in the mixed sample based on mutations that can be detected in the reads. Our study uses MAMUSS as an exemplary *mutation-based* approach based on a two-indicator classification and pre-selected marker mutations characteristic for certain lineages [20]. For the *sequence-based* approach, we use a Nextflow implementation (VLQ-nf) of the slightly adjusted VLQ pipeline as proposed by Baaijens, Zulli, and Ott *et al.* and which is based on the tool Kallisto [29]. AAF – Alternative Allele Frequency, used as a cutoff to define a mutation as a feature.

Table 2. Composition of synthetic mixture "spike-in" *Standards*. Here we show the proportions of which different SARS-CoV-2 lineages were mixed to generate a collection of artificial samples for our benchmark. For example, the sample Mix_01 comprises 25 % original Wuhan-Hu-1 A.1 and 75 % Alpha B.1.1.7 ($0.25_{org} - 0.75_{alpha}$). All samples were sequenced with Ion Torrent and raw data is available under BioProject number PRJNA912560 in the National Center for Biotechnology Information (NCBI) Sequence Read Archive (SRA). Please note that no real wastewater was used to construct these synthetic mixtures because we wanted to reduce any side effects for our *gold standard* in the context of this study.

Sample ID	Composition
Mix_01	$0.25_{org} - 0.75_{alpha}$
Mix_02	$0.25_{org} - 0.25_{beta} - 0.5_{alpha}$
Mix_03	$0.25_{alpha} - 0.25_{beta} - 0.25_{gamma} - 0.25_{org}$
Mix_04	$0.5_{org} - 0.5_{iota}$
Mix_05	$0.25_{org} - 0.25_{iota} - 0.5_{omiBA2.5}$
Mix_06	$0.25_{alpha} - 0.25_{iota} - 0.25_{omiBA2.5} - 0.25_{omiBA2}$
Mix_07	$0.5_{omiBA2.5} - 0.5_{omiBA2}$
Mix_08	$0.25_{org} - 0.25_{alpha} - 0.25_{omiBA2.5} - 0.25_{omiBA2}$
Mix_09	$0.5_{deltaAY1} - 0.5_{deltaAY2}$
Mix_10	$0.25_{deltaAY1} - 0.25_{deltaAY2} - 0.5_{delta}$
Mix_11	$0.25_{deltaAY1} - 0.25_{deltaAY2} - 0.5_{omiBA1}$
Mix_12	$0.25_{deltaAY1} - 0.25_{deltaAY2} - 0.25_{omiBA1} - 0.25_{omiBA2.5}$
Mix_13	$0.25_{deltaAY1} - 0.25_{deltaAY2} - 0.25_{omiBA1} - 0.25_{omiBA2}$
Mix_14	$0.5_{delta} - 0.25_{omiBA1} - 0.25_{omiBA2}$
Mix_15	$0.25_{deltaAY1} - 0.25_{deltaAY2} - 0.25_{omiBA1} - 0.25_{omiBA2.5}$
Mix_16	$0.25_{alpha} - 0.25_{delta} - 0.25_{omiBA1} - 0.25_{omiBA2}$

$_{org}$ – Wuhan-Hu-1 A.1; $_{alpha}$ – Alpha B.1.1.7; $_{beta}$ – Beta B.1.351; $_{gamma}$ – Gamma P.1; $_{iota}$ – Iota B.1.526; $_{delta}$ – Delta B.1.617.2; $_{deltaAY1}$ – Delta AY.1; $_{deltaAY2}$ – Delta AY.2; $_{omiBA1}$ – Omicron BA.1; $_{omiBA2}$ – Omicron BA.2 ; $_{omiBA2.5}$ – Omicron BA.2.5

tion in the following sections, we define a "false positive" hit as a lineage that was estimated with a frequency above zero without being included in the sample mixture. Analogously, we define a "false negative" hit as a lineage that was not detected by a tool even though it is included in the sample mixture by design.

VLQ-nf detected all correct spike-in lineages across all samples. The output for every sample showed, however, a certain amount of false positive predictions comprising lineages that are part of our reference set but not used as spike-ins (Figure 2). We observed the most consistent false positive estimations for Gamma (P.1) with up to 1.61 % abundance across all samples. In contrast, MAMUSS did not detect all spike-in lineages, but showed more robust results in quantifying fewer false positives in the samples (Figure 2).

When comparing false detection and over- or underestimation for both approaches, we partly observed similar patterns among specific groups of lineages: The *mutation-based* approach showed a bias in samples comprising A.1 towards not being able to detect A.1. In sample Mix_06, the *mutation-based* approach could not detect Iota (B.1.526) and falsely detected BA.1. The *sequence-based* approach considerably underestimated B.1.526 in Mix_06, whereas it falsely detected B.1.526 in Mix_01 and Mix_02.

Furthermore, both approaches showed distinct patterns of false estimation among B.1.617.2 (Delta) and its sub-lineages AY.1 and AY.2. In samples containing no Delta and only Delta sub-lineages, both approaches falsely detected Delta while underestimating AY.1 or AY.2. In samples containing only Delta and no Delta sub-lineages, MAMUSS falsely detected AY.1 and AY.2, while underestimating Delta. In samples containing Delta and Delta sub-lineages, VLQ-nf overestimated Delta and underestimated AY.1, while MAMUSS overestimated Delta sub-lineages and underestimated Delta.

Both approaches estimated BA.1 and BA.2 without distinct conflicts among each other. We observed slight over- or underestimation in the abundance of Omicron lineages to cooccur with underestimation of Delta sub-lineages in samples Mix_10–16.

Finally, we found both approaches to match the ground truth proportions of the *Standards* samples well on the parent lineage level. On the sub-lineage level, we found the false negative detection of B.1.526 in sample Mix_06 and the quantification conflicts among Delta (sub-)lineages to be the most prominent differences between both approaches. For the *mutation-based* approach, we found the false negative detection of A.1 to be the second most prominent shortcoming observed in this experiment.

VLQ-nf detects Alpha sub-lineages while MAMUSS finds distinctly larger abundances for rising lineages Beta, Gamma, and Delta in the *Pan-EU-GER* data

We analyzed German samples from the Pan-EU study [12] using both approaches to assess their performance on wastewater sequencing data. In the lack of ground truth knowledge, we evaluated both approaches by relating the lineage predictions and quantification to the pandemic background in Germany based on data from clinical sampling strategies. Moreover, we performed experiments on wastewater sequencing data to evaluate the potential benefits of wastewater-based surveillance compared to clinically-based data.

According to global surveillance projects based on clinical genomic sequence data [38, 39, 40, 41], the pandemic situation in Europe from February until April 2021 was mainly dominated by the SARS-CoV-2 lineages Alpha, Beta, cases of B.1.177 and sub-lineages, B.1.258 and sub-lineages, and B.1.160 (Supplementary Figure S1). The pandemic situation in Germany at that time was mainly dominated by VOCs Alpha and Beta as well as lineages B.1.177.86, B.1.177.81, B.1.258, B.1.177, and B.1.160. According to GISAID submissions during that time [7], approximately the same lineages and multiple other low-abundant global and European sub-lineages were reported from clinical sampling strategies. Here we focused the comparison on the lineages Alpha (B.1.1.7), Beta (B.1.351), Gamma (P.1), Delta (B.1.617.2), and the respective sub-lineages, as those were or became the dominant lineages around the time of wastewater sampling in Germany in the context of the Pan-EU project [12].

With VLQ-nf, we quantified the lineage and sub-lineage level. In comparison, MAMUSS predicted lineage abundances only at the parent level (Figure 3). Both approaches predicted Alpha (sub-)lineages to be the most abundant lineages in the data set. Specifically, the *sequence-based* approach found Alpha sub-lineages Q.1 and Q.7 to be the most abundant. Yet, those Alpha sub-lineages were not reported amongst the most frequent cases based on clinical sampling strategies (see Supplementary Figure S1). However, this is not necessarily the case, as the SARS-CoV-2 lineages can circulate in different proportions in wastewater and clinical environments. We also need to take into account the dynamic nomenclature system. The discrepancy could also be due to the retrospective definition and late classification of Alpha sublineages and again emphasizes the potential influence of reference bias. We also detected Beta, Gamma, and Delta (sub-)lineages at abundances below 1 %, which are not visible at the scale of Figure 3. In contrast, we found distinctly larger abundances of Beta, Gamma, and Delta in the samples using MAMUSS.

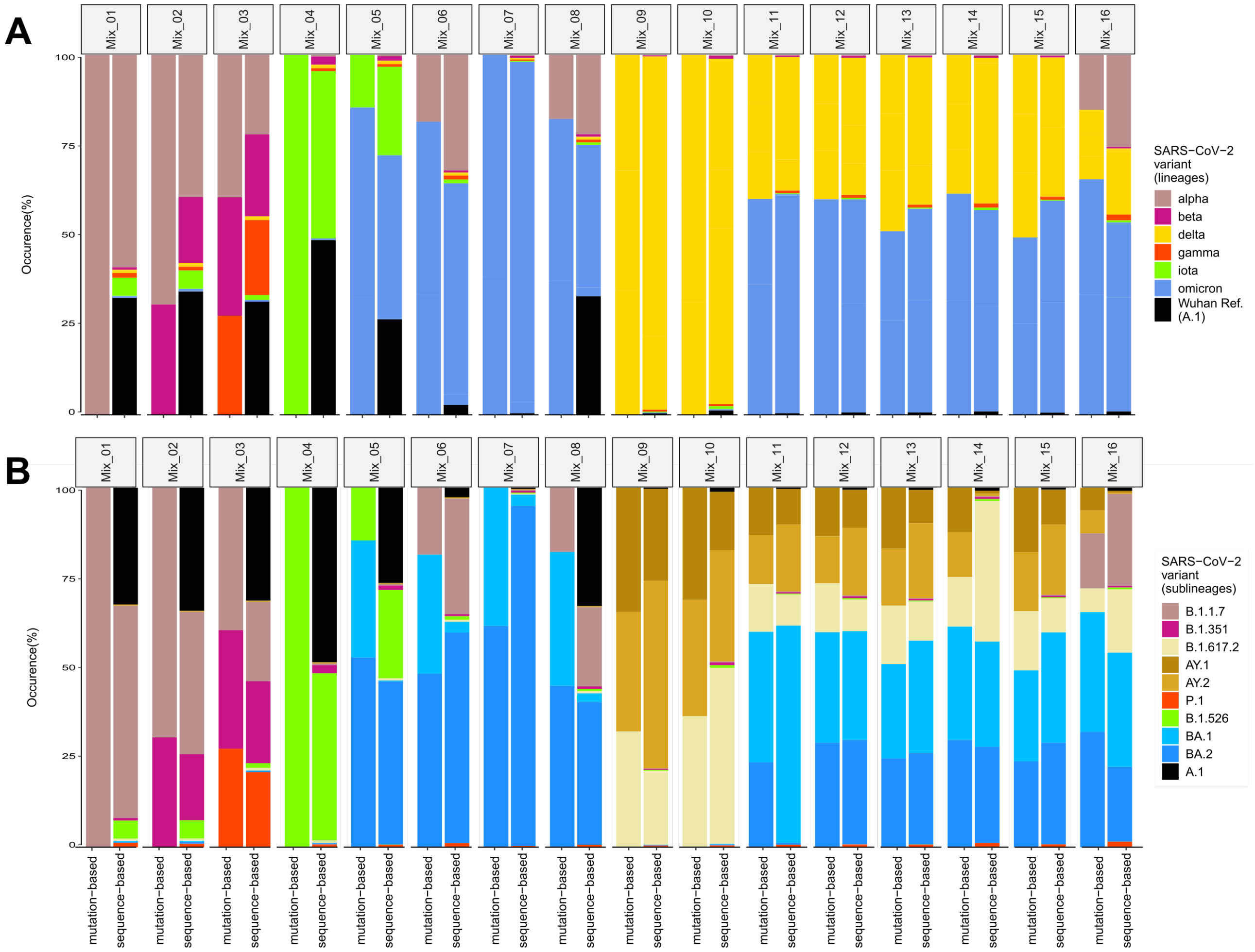

Figure 2. Comparison of the occurrence of pre-defined mixtures of SARS-CoV-2 variants (*Standards*) **(A)** at Pangolin parent lineage level and **(B)** at Pangolin sub-lineage resolution based on the *sequence-based* (VLQ-nf) and *mutation-based* (MAMUSS) approach.

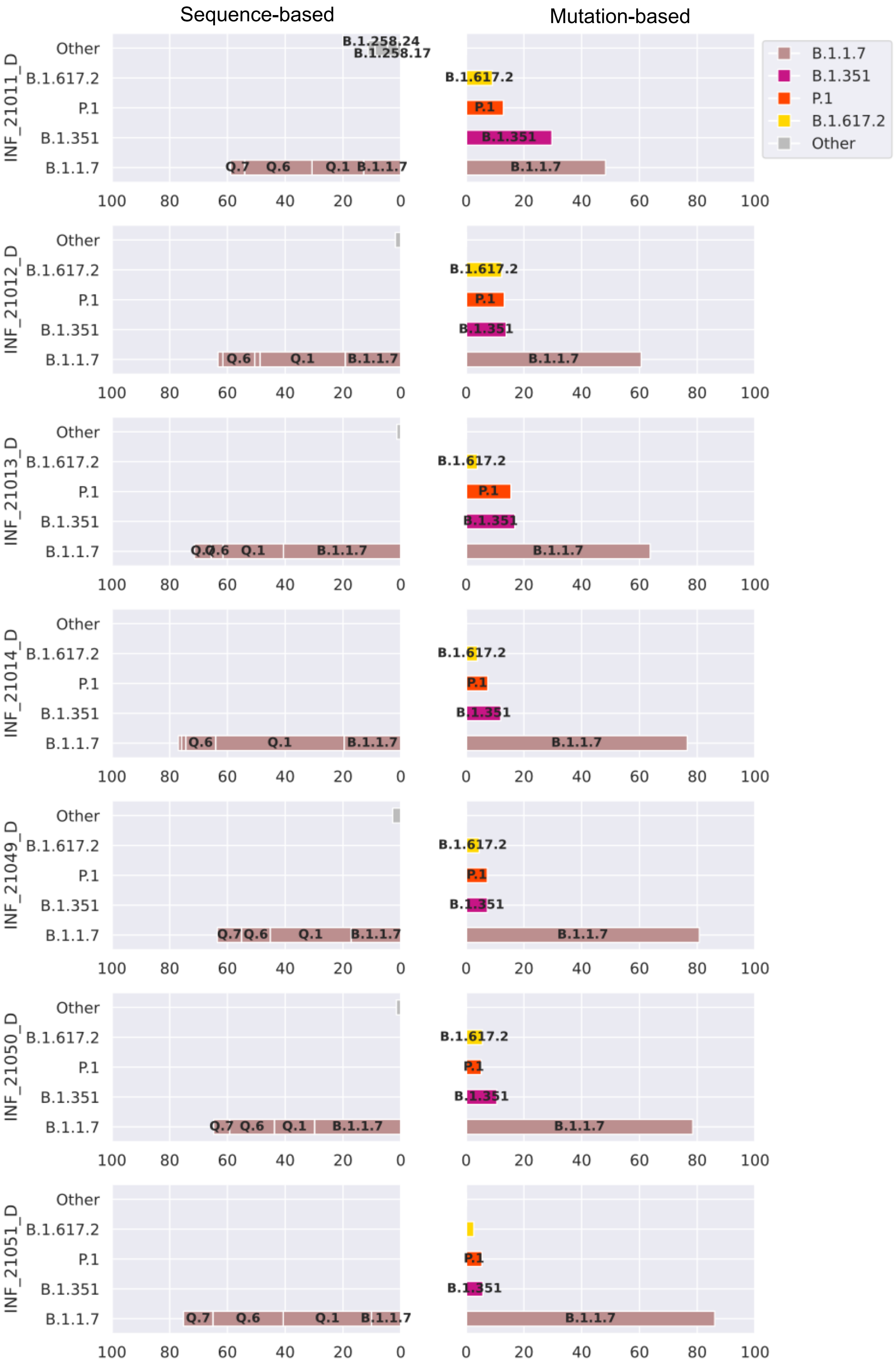

Figure 3. Comparison of the results for the *Pan-EU-GER* analysis using VLQ-nf (*sequence-based*, left) versus MAMUSS (*mutation-based*, right). Abundance predictions are plotted above a cutoff of 1 % abundance and labeled at a threshold of 3 % abundance. VLQ-nf detected abundances for B.1.617.2, P.1, and B.1.351 sub-lineages below 1 %, which is not visible at the scale of this figure. The x-axis shows the percentage of predicted lineage abundances for the *Pan-EU-GER* analysis.

Mutation- and *sequence-based* approaches recover a similar Omicron proportion from an early airport wastewater sample

We used both approaches to analyze a real wastewater sequencing sample (SRR17258654, *FFM-Airport*) [20]. We compared lineage predictions and quantification against the pandemic background in Europe and South Africa at the time of wastewater sampling. We evaluated both approaches in terms of their ability to detect (sub-)lineages at low abundances, specifically to detect low abundant signals of Omicron BA.1, which was the dominant Omicron sub-lineage circulating during that time (BA.2 was not yet detected in clinical or wastewater sequencing data).

The pandemic situation in Europe and South Africa from October to December 2021 was dominated by Delta sub-lineages and increasing incidences of Omicron and its sub-lineages [38, 39, 40, 41] (Supplementary Figure S2). According to GISAID submissions, mostly Delta sub-lineages and a few cases of Omicron and other minor global sub-lineages were reported based on clinical sampling strategies.

With VLQ-nf, we detected many Delta sub-lineages at abundances ranging from less than 1 % to around 8 % that in sum contribute over 93 % abundance in the wastewater sample (Figure 4). Roughly half of the detected Delta sub-lineages were estimated with abundances of less than 1 %. In terms of Omicron, VLQ-nf detected BA.1 with 1.44 %. Finally, we observed lineages and sub-lineages from other families with abundances of less than 1 % ("Other").

We observed a similar lineage abundance profile with MAMUSS. We found that most abundance consists of two approximately equally abundant Delta sub-lineages. We detected a small proportion close to 1 % of Omicron. Compared to VLQ-nf, we did not find any low abundant quantification for other (sub-)lineages, explained by the smaller reference data set only composed of a particular collection of marker mutations.

We found that the estimated abundance profiles of lineages from both approaches matched well with the pandemic background in Europe and South Africa at the time of wastewater sampling. However, when considering abundance estimations of the *sequence-based* approach at the sub-lineage level, we discovered differences regarding the most abundantly predicted Delta sub-lineages compared to the more prominent Delta sub-lineages derived from clinical sampling strategies in European and South African GISAID submissions (compare Figure 4 and Figure S2). The *sequence-based* approach predicted AY.25.1, AY.125.1, AY.122.4, AY.121, and AY.43.1 to be most abundant in the analyzed sample. In contrast, GISAID submissions showed AY.4, AY.43, AY.122, AY.4.2, AY.126, AY.4.2.2, and AY.98 as the most frequent Delta sub-lineages in Europe during that time. Additionally, we found AY.45, AY.32, AY.91, AY.116, AY.122, AY.6, and AY.46 to be the highest reported Delta sub-lineages in South Africa. While our predictions do not match the clinically reported frequencies, some of our predictions belong to the same lineage family as the most frequently reported lineages from clinical sampling, e.g., AY.43.1 is a sub-lineage of AY.43, AY.122.4 is a sub-lineage of AY.122, and AY.125.1 is a sub-lineage of AY.125 which we found among the twenty most frequently reported lineages in Europe using VLQ-nf.

Alternative allele frequency and size of reference database impact the *sequence-based* method, but the effects also depend on lineage composition in the sample

To better understand the impact of specific parameters on the performance of the *sequence-based* method, we performed parameter escalation experiments (see Methods) on the *Standards* benchmark set as well as the *PanEU-Ger* and *FFM-Airport* data sets. Due to the similar findings for all three data sets, here we only present the results based on the *Standards* and refer to the results of the *PanEU-Ger* and *FFM-Airport* data sets in the Supplement (subsection). We investigated the impact of reference construction parameters on lineage proportion estimation and aimed at uncovering the potential bias of the pseudoalignment implemented in the *sequence-based* method. Specifically, we focused on the AAF threshold and the maximum number of sequences per lineage. The AAF threshold defines the minimum alternative allele frequency for a mutation to be considered characteristic of a lineage. First, genome sequences are added as lineage references so that each mutation that exceeds the AAF threshold is detected at least once by as few sequences as possible. Next, additional genomes are added until the maximum number of sequences per lineage is reached. Thus, the AAF threshold controls the level of genomic variation captured for each lineage and the maximum number of sequences per lineage controls the reference size.

Standards

Across most *Standards* samples and experiments, VLQ-nf detected all spike-in lineages and predicted reasonable estimates (Figure 5). However, we consistently observed low abundant false positive hits in all of our mixed samples, comprising lineages that are part of the reference index but not used as spike-ins. We found the most prominent false positive detection to be Gamma. We observed similar patterns of false positive detection and false estimation among specific groups of lineages across all parameter settings: For the first eight samples Mix_01 to Mix_08, most cases of false estimation of spike-in lineage abundances occurred alongside false positives or negatives of B.1.526 and false positives of BA.1. For the samples Mix_09 to Mix_16, we observed most detection conflicts to involve ambiguities among Delta and its sub-lineages AY.1 and AY.2.

We found that the detection and quantification performance of the *sequence-based* method via VLQ-nf changed with varying thresholds for the alternative allele frequency and maximum number of genomes per reference lineage. Specifically, we found those changes to vary across samples and observed them not to behave identically with consistent parameter changes. For example, at the minimum reference size (Supplementary Table S1), we observed abundance predictions for samples Mix_09 and Mix_11-16 to first improve with an increasing AAF threshold. However, with a further increasing AAF threshold, we observed more false estimations of Delta sub-lineages. Furthermore, although Mix_10 shares most of its spike-in lineages with Mix_09, the performance of abundance estimations for sample Mix_10 first decreased and then improved again when increasing the AAF threshold.

We made a similar observation for the maximum number of sequences per lineage. With an AAF threshold of 0.5, the abundance estimates for Mix_01 improved with increasing number of reference genomes per lineage, while we found them to deteriorate for Mix_09, which includes a distinctly different sample composition. Overall, we found lineage abundance estimations to become slightly more robust across varying AAF thresholds with increasing reference size. This is best reflected in the abundance profiles for samples Mix_09–Mix_15 when looking at the proportional changes across increasing AAF settings for the minimum reference size throughout the reference with 10 sequences per lineage.

Finally, we found that the AAF threshold and the reference size affect the performance of the *sequence-based* method. Although we did not observe a clear and consistent pattern of impact, we found that the effects of varying parameter settings may depend on the sample composition. Specifically, we ob-

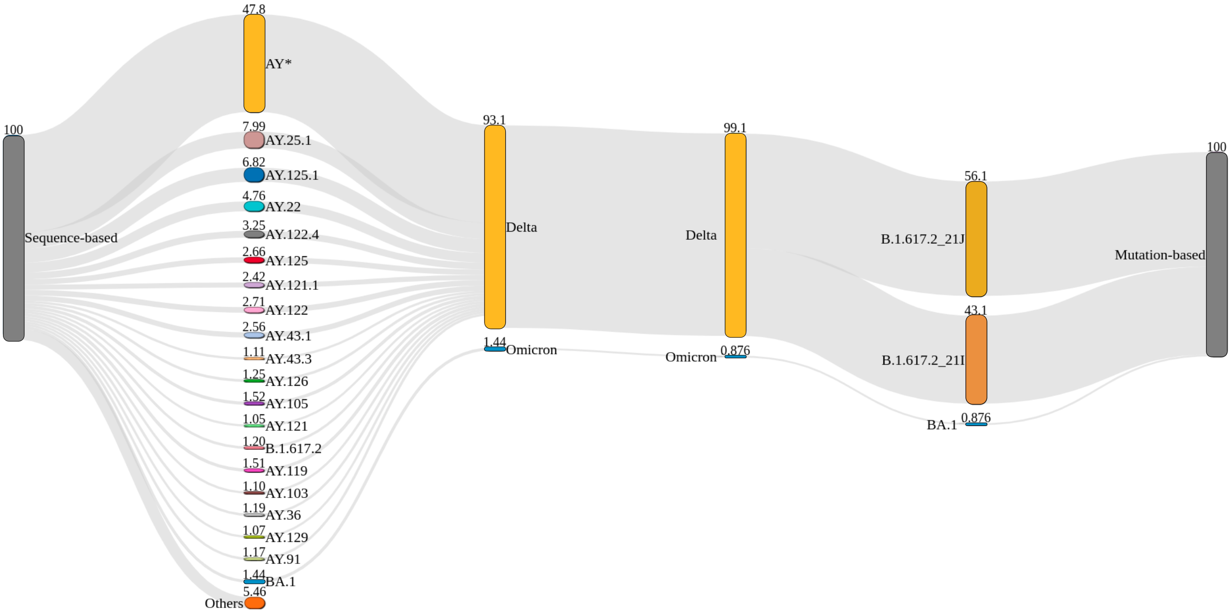

Figure 4. Sankey plot comparing the detected lineage proportions for the *sequence-based* approach (VQL-nf, left) and the *mutation-based* approach (MAMUSS, right) for one airport wastewater sample (SRR17258654) [20]. Both approaches detect a similar amount of Delta and Omicron (BA.1) in the sample. At the same time, VQL-nf can achieve a higher sub-lineage resolution (AY lineages) based on the full genome information in the reconstructed reference index and utilizing pseudo-alignments. MAMUSS can, as configured for this analysis and based on the limited reference set, distinguish between two slightly different B.1.617.2 clades as defined by Nextstrain. For the *sequence-based* approach, only lineages with a proportion of at least 1 % are shown and all other AY-sub-lineages are pooled in AY* and all other lineages in "Others".

served the strongest impact of parameter changes for samples containing lineages with a higher degree of shared genomic similarity. Also, we found the AAF threshold to affect estimates slightly more than the reference size. We detected similar results for the *PanEU-Ger* and *FFM-Airport* data sets. We provide details for these two data sets in the Supplement (see Figure S3 and Figure S4).

Final choice of parameters for benchmark reference construction

Within the scope of the parameter escalation experiments described here, we wanted to determine parameters with a good prediction performance without manipulating the benchmark in favor of the *sequence-based* approach (VQL-nf). Finally, based on our parameter testing and the three different data sets, we chose an AAF threshold of 0.25 and a reference size of at most 5 sequences per lineage. This threshold allowed us to limit the size of the reference data set and still allows reasonable detection and quantification results across all three benchmark data sets, while keeping computational resources moderate.

Discussion

It is apparent that the composition of the reference used must have a large impact on the determination of relative SARS-CoV-2 abundances in wastewater sequence data. Especially given the dynamic and constantly updated SARS-CoV-2 lineage definitions [3], the reference genome sequences and the signature mutations derived from them also change frequently. Of course, the various tools (Table 1) and their parameters developed for estimating the relative abundance of lineages from wastewater sequencing data also have an impact. Here, however, we have specifically focused on the effects of the reference design.

We selected two general approaches to design reference data sets and estimate SARS-CoV-2 lineage proportions from wastewater sequencing samples (Figure 1). On the one hand, selected marker mutations that are characteristic for certain SARS-CoV-2 lineages can be used for annotation and lineage proportion estimation (*mutation-based*, MAMUSS). Here, the read sequences derived from a wastewater sample are mapped against a reference genome from which differences (mutations) are detected and compared against the selected marker mutations. On the other hand, full SARS-CoV-2 genome sequences can be used to create a reference index without prior collection of specific mutations (*sequence-based*, VLQ-nf). Here, the problem of selecting appropriate marker mutations is shifted to selecting representative lineages from which features for the classification task are derived. An exemplary implementation of this approach based on the pseudo-aligner Kallisto [32] was recently proposed by Baaijens, Zulli, and Ott *et al.* [29]. Based on their work, we developed a Nextflow pipeline for higher automation and reproducibility and detecting SARS-CoV-2 lineage proportions from wastewater data using pseudo-alignments (VLQ-nf). In this approach, a selection of whole-genome SARS-CoV-2 sequences (target reference set) and the reads (query) are composed into kmers which are then efficiently compared to quantify lineage abundances, similar to quantifying gene expression in an RNA-Seq study.

To benchmark reference designs from both methods (*mutation-based* via MAMUSS, *sequence-based* via VLQ-nf), we selected three test scenarios: 1) a spike-in experiment with different SARS-CoV-2 lineage mixes, 2) samples obtained for Germany from a Pan-EU wastewater study, and 3) a wastewater sample from a German airport during the time when Omicron emerged.

In general, both approaches detected SARS-CoV-2 lineage abundances from our test cases. The most remarkable difference was in the number of detected sub-lineages which also directly correlates with the reference design. VLQ-nf generally detected a larger diversity of sub-lineages in comparison to MAMUSS, which can be explained by the underlying reference indices. It became increasingly difficult to select a representative set of marker mutations for the *mutation-based* approach and the implementation we used as more and more

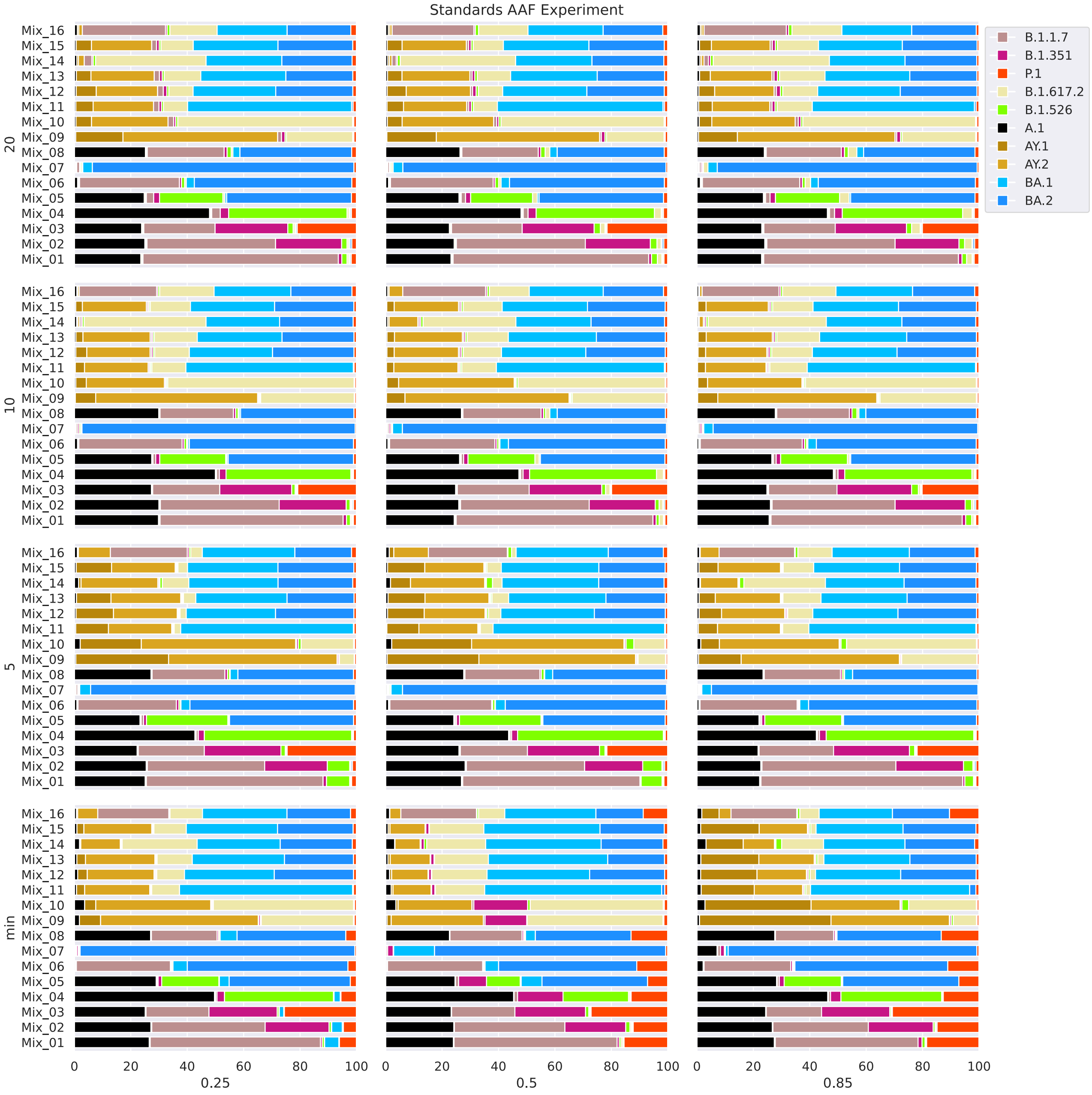

Figure 5. Results for the parameter escalation experiments on the *Standards* samples using the *sequence-based* method (VLQ-nf). We analyzed the *Standards* with different parameterizations for reference construction (x-axis: increasing AAF threshold, y-axis: increasing maximum number of sequences per lineage). VLQ-nf, using pseudo-alignments, detected all lineages and estimated abundance profiles well across most samples and parameter settings. However, we also observed prominent detection ambiguities among Delta and its sub-lineages and found consistently low abundant false positives for specific groups of lineages. Continuously increasing or decreasing parameter settings caused heterogeneous changes in the estimated abundance proportions across samples. The *sequence-based* method showed to perform better when using a reference set larger than the minimum reference size. Still, we found noise levels to increase distinctly when using the maximum reference size among the considered settings.

(sub)lineages were defined and there was overlap in mutations (convergent evolution). In contrast, the *sequence-based* approach as suggested by Baaijens, Zulli, and Ott *et al.* [29] can build a reference index on a large collection of SARS-CoV-2 full genome sequences derived from clinical samples and thus, potentially, better reflect diversity on sub-lineage levels. However, we also observed a certain amount of *noise* in the pseudo-alignment results causing potential false-positive hits in our test data sets. Other approaches, like Freyja [21], partly tackle this problem by deriving signature mutation profiles automatically, for example, using the whole phylogenetic diversity of current SARS-CoV-2 sequences reflected in an UShER tree [34]. However, here we have also observed that the inclusion of a large diversity in the reference can lead to distributed abundance assignments between closely related (sub)-lineages, reducing the true relative abundance of a lineage (Figure S5 and Figure S6). Of course, the impact can be reduced by limiting lineage coverage to a specific time period, but this, in turn, can also affect frequency assignments.

In more detail, both approaches performed similarly in detecting and estimating spike-in lineage abundances for the *Standards* data set Figure 2. The predictions are more similar on the parent-lineage level compared to the sub-lineage level. If their estimations differ, this can be mostly attributed to differences in the mutations/lineages included in the respective reference data: for both approaches, the final predictions heavily depend on the construction of the reference data set. In addition, both approaches had difficulties differentiating closely related sub-lineages correctly.

For the *Pan-EU-GER* data set, both approaches reflect well the pandemic background in Germany during the time of sampling, but we detected some limitations and potential sources for bias: The choice of marker mutations and reference lineages impacts the level of detection, i.e. lineage vs. sub-lineage level estimations, but also the amount of low abundance detection. Potentially, everything that is defined in the reference data set can also be detected, which might lead to an increased number of false positive predictions. The whole-genome sequences or mutations used to create the reference index impact the degree of ambiguity and, thus, (low abundant) false positive detection. This may explain why both approaches predicted distinctly different abundances on the parent-lineage level compared to the other two benchmark experiments. Therefore, we think that especially the *sequence-based* approach requires the definition of a false positive threshold to differentiate between low abundant false positive hits and low abundant true positives.

Both approaches also detected low-frequency lineages for the *FFM-Airport* data set. Again, the *sequence-based* approach detects a distinctly higher amount of low abundant lineages, also reflecting the higher diversity of the reference index.

We performed an additional parameter benchmark to identify important key parameters impacting the *sequence-based* pseudo-alignment approach using VLQ-nf. One parameter that strongly affects the results is the alternative allele frequency (AAF) cutoff. In connection with the reference size (the number of genomes), we observed different effects of changing the AAF. Our experiments also showed that the effect of the same parameter changes (increasing or decreasing AAF) does not yield consistent results among the different data sets. The degree of lineage ambiguity depends on the considered composition of lineages and sub-lineages. The effect of included/excluded mutations due to adjusted AAF parameter settings is variable, as different mutations have different effects in differentiating lineages. The effect of those parameter changes is most notable among more similar lineages. We also observed that with a larger reference size, the effect of the AAF parameter becomes smaller and overall abundance estimations improve. One explanation might be that by adding further reference genomes for a lineage, low-frequency mutations are implicitly introduced and increase the genomic variation that is represented by the reference data set. These additional low-frequency mutations might support the differentiation of certain (sub-)lineages better and thus slightly improve abundance estimations.

Potential implications

In this study, we focus exclusively on Ion Torrent sequencing data to specifically investigate the influence of reference database composition and analysis parameters on lineage abundance estimates in wastewater sequencing. While acknowledging that incorporating data from additional platforms like PacBio, Nanopore, and Illumina could broaden the analysis of variability and robustness, we chose Ion Torrent due to its established efficacy in achieving high horizontal genome coverage in our sequencing runs [20, 12, 37], critical for assessing the impact of reference bias. This focused approach allows us to explore the considerable effects that reference selection and analytical settings have on lineage abundance results, a crucial area for accurate viral surveillance. Future studies might explore a comparative analysis across different platforms to enhance understanding of lineage composition and abundance estimation in wastewater samples. However, our current study is intentionally limited to specific research objectives related to reference bias in a *mutation-based* and *sequence-based* setting and in the context of declining clinical sequencing and the dilution of available reference sequences.

Further, we only selected two exemplary implementations of the *mutation-* and *sequence-based* approaches MAMUSS and VLQ-nf, respectively, out of an increasing number of scripts, tools, and pipelines becoming available for computational SARS-CoV-2 lineage estimation from wastewater sequencing (Table 1) [21, 22, 23, 24, 16, 25, 26, 18, 10, 27, 28, 29, 30, 31]. Thus, our benchmark results also reflect and are limited by the individual characteristics of these two implementations. However, we focused on these two approaches to investigate the impact of reference design using implementations where we could easily control parameters and input – similar to the decision for the Ion Torrent technology. Currently, a comprehensive benchmark comparison for the existing SARS-CoV-2 wastewater analysis tools is lacking. The developers of Freyja compared a selection of tools on a spike-in mixed sample [21] where they found that Freyja outperformed VLQ [29] in accuracy at higher expected proportions and observed noticeably longer computation times for both VLQ and LCS [23]. To counteract the effect on lineage abundance detection, some methods filter the mutations considered for lineage assignment based on sequencing depth [16] or adjust their mathematical model for differences in depth and coverage and expected error rates [21, 27]. Similarly, the PiGx tool addresses the limitations of estimating lineages at low abundances by weighting specific signature mutations for lineages that are expected to occur at low frequencies [26]. Another recent study compared nine computational tools but only used simulated genomic data [33]. As a next step, a broader evaluation of all available tools for analyzing SARS-CoV-2 wastewater sequencing data is urgently needed to guide usage and further development [42].

Conclusion

Academic researchers have pioneered wastewater monitoring of SARS-CoV-2 and overcome several technical and methodological challenges [15]. Thanks to these efforts, wastewater-based pathogen surveillance has rapidly become a valuable pub-

lic health tool for detecting SARS-CoV-2 that can excellently complement syndromic surveillance or other monitoring tools. However, public health authorities are now faced with the task of integrating these achievements into robust and continuous public health surveillance systems that can be operated and expanded over the long term. Performance parameters must be defined and communicated to the public health authorities to include wastewater-based pathogen surveillance data. In this context, continuous updating of reference data sets, in the context of retrospective analyses or time series, is essential to ensure comparability between time points. For example, genomic sequences of newly defined lineages might already be present in wastewater samples from previous weeks. However, bioinformatic analysis of previous samples could not detect the novel lineage because it was not included in the reference data set at that time point. Continuously updated reference data sets can support comparing and interpreting wastewater sequencing time series data. Yet, harmonizing the reference used would require recalculating older abundance estimates, which may conflict with the standard reporting requirements of public health authorities. However, this problem is not specific to wastewater-based SARS-CoV-2 sequencing data, but also applies to genomics sequencing of patient samples. One solution might be to focus not only on lineages, but also to report mutations that are not affected by any nomenclature scheme and are not subject to delayed definitions. On the other hand, it is undeniable that lineages played a crucial role in communication during the COVID-19 pandemic. Recently, McBroome *et al.* proposed a novel framework for a more automated and scalable designation of viral pathogen lineages from (clinical) genomic data [43].

Wastewater sequencing data also offers the potential to uncover *cryptic* (novel, undescribed) lineages, although resolving the full genomic profile of those solely from wastewater data still poses several challenges [21, 11]. In this context, approaches utilizing artificial intelligence might present a promising next step for the improved detection of cryptic SARS-CoV-2 lineages from wastewater sequencing data and increasing trends, although right now, not much in use [44]. However, first studies appear that use machine learning for the early detection of new signals from wastewater data and the description of potential new SARS-CoV-2 lineages [45, 46]. Finally, the lessons learned from the sequencing efforts and implementations for SARS-CoV-2 detection from wastewater sequencing data can and should be adapted to other pathogens to further advance wastewater genomic surveillance efforts.

Methods

Benchmark data set #1: *Standards*

We procured synthetic SARS-CoV-2 RNA samples (Twist Biosciences), which were used to prepare 16 different mixtures (Table 2) containing different SARS-CoV-2 variants. From the pooled RNA, cDNA was synthesized using SuperScript™ VILO™ Master Mix (Thermofisher Scientific), followed by library preparation using the Ion AmpliSeq SARS-CoV-2 Research Panel (Thermofisher Scientific) according to the manufacturer's instructions. This panel consists of 237 primer pairs, resulting in an amplicon length range of 125–275 bp, which cover the near-full genome of SARS-CoV-2. We performed two sequencing runs to achieve at least 1 million mapped reads per sample. For each sequencing run, eight libraries were multiplexed and sequenced using an Ion Torrent 530 chip on an Ion S5 sequencer (Thermofisher Scientific) according to the manufacturer's instructions. The raw sequence data were uploaded to the National Center for Biotechnology Information (NCBI) Sequence Read Archive (SRA) under BioProject number PRJNA912560.

Data processing: *mutation-based* reference design and lineage proportion estimation via MAMUSS

We used the SARS-CoV-2 Research Plug-in Package, which we installed in our Ion Torrent Suite software (v5.12.2) of Ion S5 sequence. We used the SARS_CoV_2_coverageAnalysis (v5.16) plugin [47], which maps the generated reads to a SARS-CoV-2 reference genome (Wuhan-Hu-1-NC_045512/MN908947.3), using TMAP software included in the Torrent Suite. The summary of mapping of each sample mentioned in Table 2 is provided in Table S2. For mutation calls, additional Ion Torrent plugins were used as described previously [37] and detailed below. First, all single nucleotide variants were called using Variant Caller (v5.12.0.4) with "Generic - S5/S5XL (510/520/530) - Somatic - Low Stringency" default parameters. Then, for annotation and determination of the base substitution effect, we used COVID19AnnotateSnpEff (v1.3.0.2), a plugin developed explicitly for SARS-CoV-2 and based on the original SnpEff [48]. To construct reference marker mutation sets for MAMUSS, we used data from GISAID [8]. For each SARS-CoV-2 variant, we downloaded the variant surveillance database and selected complete clinical genome sequences, followed by counting the prevalence of its associated mutations. The fifty most prevalent mutations associated with each variant were used as reference marker mutation set. The lineage abundance estimation is based on the read depth and allele frequency of each mutation detected in a wastewater sample followed by a two-indicator classification and comparison to the pre-selected marker mutations characteristic for certain lineages. For further details see the MAMUSS GitHub repository [49].

Data processing: *sequence-based* reference design and lineage proportion estimation via VLQ-nf

Instead of relying only on manually or algorithmically selected marker mutations, another computational approach utilizes, in a first step, full genome information. For example, Baaijens, Zulli, and Ott *et al.* presented a method to estimate the abundance of variants in wastewater samples based on well-established computational techniques initially used for RNA-Seq quantification [29]. Here, the main idea is that quantifying different transcripts derived from the same gene is computationally similar to the abundance estimation of different SARS-CoV-2 lineages derived from the same parental genome. Via Kallisto [32], they perform pseudo-alignments of the raw reads against an index of pre-selected and down-sampled full genome SARS-CoV-2 sequences with respective lineage information. Therefore, their approach may be less influenced by the pre-selection of mutations based on clinical relevance, frequency, or other parameters that mostly drive *mutation-based* tools, and thus may be better suited for sub-lineage discrimination. The approach comprises two steps: 1) selecting reference genome sequences for index construction and 2) pseudo-alignment of the reads and lineage abundance estimation. First, a reference data set of SARS-CoV-2 genome sequences must be selected. For that, we use data from GISAID [8] and filter for human-host sequences, N-count information, pangolin annotation [3, 2], origin (country, continent), and sampling date. This metadata is used to pre-select sequences based on geographic origin (continent, country), a sampling time frame, and the number of N bases. Next, the pipeline performs a variant calling against a reference sequence (per default index Wuhan-Hu-1, NC_045512.2) and subsequently samples sequences to select characteristic mutation profiles for

Table 3. For each lineage in the *Standards* data set, we selected the time frame where infection numbers peaked globally [38]. Based on the listed time frames, we sampled genome sequences from GISAID for reference reconstruction. We downloaded the GISAID records on 02 March 2022.

Lineage	Time frame
A.1	2020-03-01:2020-03-14
B.1.1.7	2021-05-01:2021-05-14
B.1.351	2021-01-20:2021-02-02
P.1	2021-04-20:2021-05-03
B.1.526	2021-03-20:2021-04-02
BA.2	2022-02-01:2022-02-14
BA.1	2021-12-01:2021-12-14
B.1.617.2	2021-06-25:2021-07-08
AY.1	2021-08-01:2021-08-14
AY.2	2021-06-25:2021-07-08

each input lineage. Within a lineage, sequences are sampled based on an alternative allele frequency cutoff (e.g., AAF>0.5) so that each mutation is represented at least once until an upper limit of sequences per lineage is reached. From this downsampled and representative set of full genome sequences, a Kallisto index is constructed. Now, the raw reads from a FASTQ file are pseudo-aligned against this index and lineage abundances are quantified. This is done by estimating for each read the probability of originating from each genome sequence in the reference using expectation maximization, and finally aggregating the resulting probabilities across the lineage labels associated with every reference genome.

For our comparative study, we used the initial idea and code base from Baaijens, Zulli, and Ott *et al.*[29, 50] and implemented a Nextflow pipeline [35, 36] with the purpose of automating the steps and making our analyses fully reproducible. In this context, we discovered some issues in the pipeline version 61dd29df* of Baaijens, Zulli, and Ott *et al.* and implemented minor adjustments. This includes updating data processing scripts according to the most recent GISAID data format and allowing the sequence selection based on alternate allele frequencies to consider multi-allelic sites. Meanwhile, the authors have addressed those issues with similar code changes in their current pipeline version. In pipeline version 61dd29df*, sequences are selected for the reference index if they carry an AAF filter passing mutation that is not yet covered until the reference set for the respective lineage meets the maximum allowed number of sequences. We wanted to provide the possibility for using a minimum reference setup to reduce data storage requirements and allow exploring the impact of different AAF thresholds on abundance estimation. Subsequently, we adjusted the AAF filter to first sample a minimum set of genome sequences so that all passing mutations are included at least once, before increasing the reference set to the number of maximum sequences per lineage. We ran our pipeline version v1.0.0 for all analyses in this benchmark study.

Reconstruction of indices for the *sequence-based* approach

The *sequence-based* (VLQ-nf) approach highly depends on the selection and reconstruction of the reference data set for the Kallisto index. Thus, we reconstructed different indices for our three benchmark data sets to mimic the pandemic situation during the time of sampling. We used GISAID data for all indices and extracted subsets based on metadata filters.

For the benchmark of the 16 mixed *Standards*, we constructed a reference data set comprising the included SARS-CoV-2 lineages. We selected a time frame of two weeks around the peak of global incidences[39?] for each lineage included in the mix (Table 3). We only kept records with at least 29,500 non-ambiguous bases. Because we also included the original Wuhan-Hu-1 reference sequence in mixed samples Mix_01–Mix_05 and Mix_08, we first excluded all A.1 sequences from the preselected set. Then, we selected reference sequences with characteristic mutation profiles for all lineages except A.1 as described before allowing a maximum number of five sequences per lineage. Then, we added the sampled A.1 sequences again to the final reference set, as otherwise the A.1 sequences would have been excluded by the pipeline because they don't show any AAF in comparison to the Wuhan-Hu-1 reference. On average, we selected five sequences for a lineage to capture every mutation against the wildtype with an AAF>0.25 (within-lineage variation) and a maximum of five allowed sequences per lineage.

For the *Pan-EU-GER* samples (collected between 10th and 30th March 2021), we reconstructed the reference from clinical GISAID records we downloaded on 27 January 2022. We selected only European sequences sampled between February 1st, 2021, and April 30nd, 2021, with at least 29,500 non-ambiguous bases. To reflect the influx of variants from other European countries, we have not only selected sequences from Germany. On average, we then selected three sequences per lineage to capture every mutation against the wildtype with an AAF>0.25 (within-lineage variation) and allowing at most five reference sequences per lineage.

For the *FFM-Airport* data set, we reconstructed the reference from GISAID records we downloaded on 11 February 2022. We selected genome sequences from European and South African clinical records sampled between October 1st, 2021, and December 31st, 2021, again with at least 29,500 non-ambiguous bases. On average, four sequences were selected for a lineage to capture every mutation against the wildtype with an AAF>0.25 (within-lineage variation). Again, we allowed at most five sequences to be included per lineage.

Lineage-abundance estimation with the *sequence-based* approach

After reconstructing different reference indices for our benchmark data sets, we used specific Kallisto commands implemented in a Nextflow pipeline to prepare Kallisto mapping indices, compute pseudo-alignments of each benchmark data set against its reference index, and estimate lineage abundances following the original idea and code of Baaijens, Zulli, and Ott *et al.*[29].

First, we built a Kallisto index from the reference database (default k-mer=31). Next, for each sample in a benchmark data set, we pseudo-aligned all reads against the corresponding Kallisto index and estimated the abundance of each reference sequence in the sample. We quantified our benchmark data sets in single reads mode with an average fragment length of 200 nt with a standard deviation of 20 nt. Finally, a customized script groups the estimated abundances by the lineage annotation of the respective sequences and sums them up into a final lineage abundance estimation for the analyzed sample. For the *Pan-EU-GER* and *FFM-Airport* data sets, we further summarized the estimated abundances by the country information of the analyzed samples to compare the pseudo-alignment and *mutation-based* approach on the country level.

Assessing parameter impact and potential bias with the pseudo-alignment approach

We performed parameter escalation experiments with our three benchmark data sets using the *sequence-based* method (VLQ-

nf) to assess the impact of the AAF threshold and the cut-off for a maximum number of sequences per lineage on lineage abundance estimation. More importantly, we used the resulting observations to inform our choice of parameters used for the final benchmarking against the *mutation-based* method (MAMUSS). In this context, we aimed to determine a setting with a good prediction performance and reasonable computational effort without manipulating the benchmark in favor of the *sequence-based* method. For every benchmark data set, we constructed reference indices over a range of 12 possible parameter combinations. For the AAF threshold, we iterated over [0.25, 0.5, 0.85] to cover lower, medium, and high threshold values to define the characteristic mutation profiles. For the maximum number of sequences per lineage, we built the reference index using the minimal sequence sets possible, 5, 10, and 20 sequences per lineage. After lineage abundance estimation with each reference index on the *Standards* data set, we evaluated prediction performance based on the ground truth lineage abundances. For the *FFM-Airport* and *Pan-EU-GER* data, we assessed prediction performance by comparing estimated lineage abundances with the pandemic background at the respective time and location.

Reproducibility of the pseudo-alignment approach

Our Nextflow pipeline of the pseudo-alignment approach [36] generates the reference database in the format of a CSV file containing the metadata information of the final Kallisto index and a FASTA file containing the corresponding sequence data. In the current version v1.0.0, the reference CSV and FASTA can be exactly replicated using the same input data resource and index reconstruction parameters which leads to slightly different results at every analysis run. The reference CSV is not reproducible due to misplaced random sampling seeds and a missing record sorting strategy in the AAF-based sequence filtering step during reference reconstruction. However, lineage detection and quantification are deterministic given VLQ-nf takes fixed reference data sets as input (final CSV and FASTA reference or already built Kallisto index).

Availability of source code and requirements

Here, we provide the specifications of our Nextflow implementation (VLQ-nf) of the *sequence-based* approach originally presented by Baaijens, Zulli, and Ott *et al.*[29] and the code for the *mutation-based* approach, MAMUSS.

- Project name: VLQ-nf
- Project home page: https://github.com/rki-mf1/VLQ-nf
- Operating system(s): Linux, Mac, Windows via Linux subshell
- Programming language: Nextflow
- Other requirements: Conda
- License: GPL-3.0

- Project name: MAMUSS
- Project home page: https://github.com/lifehashopes/MAMUSS
- Operating system(s): Linux, Mac
- Programming language: R
- Other requirements: R packages are listed in the repository
- License: CC0 1.0 Universal

Data Availability

The data sets supporting the results of this article are available in the Open Science Framework repository [51]. All supporting data and materials are available in the GigaScience GigaDB database [52].

Declarations

List of abbreviations

- AAF – alternative allele frequency
- FFM-Airport – one sample from the end of 2021 including first signals of the VOC Omicron obtained from wastewater at the international airport in Frankfurt am Main, Germany [20]
- MAMUSS – *mutation-based* approach for SARS-CoV-2 lineage abundance estimation
- Pan-EU-GER – seven samples from early 2021 from a large European study and collected in Germany, mainly comprising the VOC Alpha [12]
- Standards – synthetic scenario of 16 “spike-in” mixture SARS-CoV-2 samples
- VLQ-nf – *sequence-based* approach for SARS-CoV-2 lineage abundance estimation, inspired by the original VLQ [29]
- WBE – wastewater-based epidemiology

Ethical Approval (optional)

Not applicable.

Consent for publication

Not applicable.

Competing Interests

The authors declare that they have no competing interests

Funding

Author’s Contributions

SA, SL, and MH provided conceptualization and study design. SA implemented the MAMUSS approach and analyzed corresponding data. EA implemented the VLQ-nf approach and analyzed corresponding data. SA and LO conducted wet lab experiments to generate and sequence synthetic mixtures. EA, SA, and MH performed the computational comparisons and generated the figures. All authors actively participated in the writing and editing of the manuscript. All authors have read and agreed to the published version of the manuscript.

Acknowledgements

We gratefully acknowledge all data contributors, i.e., the Authors and their Originating laboratories responsible for obtaining the specimens, and their Submitting laboratories for generating the genetic sequence and metadata and sharing via the GISAID Initiative, on which this research is based.

References

1. World Health Organization, WHO Coronavirus (COVID-19) dashboard;. https://covid19.who.int/, accessed: April 30, 2024.
2. O'Toole Á, Scher E, Underwood A, Jackson B, Hill V, McCrone JT, et al. Assignment of epidemiological lineages in an emerging pandemic using the pangolin tool. Virus evolution 2021;7(2):veab064.
3. Rambaut A, Holmes EC, O'Toole Á, Hill V, McCrone JT, Ruis C, et al. A dynamic nomenclature proposal for SARS-CoV-2 lineages to assist genomic epidemiology. Nature microbiology 2020;5(11):1403–1407.
4. cov-lineages, Pango Cov-Lineages website data. GitHub; 2021. https://github.com/cov-lineages/lineages-website/blob/master/_data/lineage_data.full.json, accessed: commit hash 0dcb1c4* (April 19, 2023).
5. consortium TCGUCU. An integrated national scale SARS-CoV-2 genomic surveillance network. The Lancet Microbe 2020;3(1):E99–E100.
6. Robishaw JD, Alter SM, Solano JJ, Shih RD, DeMets DL, Maki DG, et al. Genomic surveillance to combat COVID-19: challenges and opportunities. The Lancet Microbe 2021;2(9):e481–e484.
7. Oh DY, Hölzer M, Paraskevopoulou S, Trofimova M, Hartkopf F, Budt M, et al. Advancing Precision Vaccinology by Molecular and Genomic Surveillance of Severe Acute Respiratory Syndrome Coronavirus 2 in Germany, 2021. Clinical infectious diseases 2022;75(Supplement_1):S110–S120.
8. Shu Y, McCauley J. GISAID: Global initiative on sharing all influenza data – from vision to reality. Eurosurveillance 2017;22(13):30494. Https://gisaid.org/.
9. Robert Koch-Institut, SARS-CoV-2 Infektionen in Deutschland; 2024. https://robert-koch-institut.github.io/SARS-CoV-2-Infektionen_in_Deutschland.
10. Jahn K, Dreifuss D, Topolsky I, Kull A, Ganesanandamoorthy P, Fernandez-Cassi X, et al. Early detection and surveillance of SARS-CoV-2 genomic variants in wastewater using COJAC. Nature microbiology 2022;7(8):1151–1160.
11. Smyth DS, Trujillo M, Gregory DA, Cheung K, Gao A, Graham M, et al. Tracking cryptic SARS-CoV-2 lineages detected in NYC wastewater. Nature communications 2022;13(1):1–9.
12. Agrawal S, Orschler L, Schubert S, Zachmann K, Heijnen L, Tavazzi S, et al. Prevalence and circulation patterns of SARS-CoV-2 variants in European sewage mirror clinical data of 54 European cities. Water research 2022;214:118162.
13. Peccia J, Zulli A, Brackney DE, Grubaugh ND, Kaplan EH, Casanovas-Massana A, et al. Measurement of SARS-CoV-2 RNA in wastewater tracks community infection dynamics. Nat Biotechnol 2020 Oct;38(10):1164–1167. https://www.nature.com/articles/s41587-020-0684-z.
14. Nemudryi A, Nemudraia A, Wiegand T, Surya K, Buyukyoruk M, Cicha C, et al. Temporal detection and phylogenetic assessment of SARS-CoV-2 in municipal wastewater. Cell Reports Medicine 2020;1(6):100098.
15. Hoar C, McClary-Gutierrez J, Wolfe MK, Bivins A, Bibby K, Silverman AI, et al. Looking Forward: The Role of Academic Researchers in Building Sustainable Wastewater Surveillance Programs. Environmental Health Perspectives 2022;130(12):125002.
16. Amman F, Markt R, Endler L, Hupfauf S, Agerer B, Schedl A, et al. Viral variant-resolved wastewater surveillance of SARS-CoV-2 at national scale. Nature Biotechnology 2022;40(12):1814–22.
17. Munteanu V, Saldana M, Sharma NK, Ouyang WO, Aßmann E, Gordeev V, et al. SARS-CoV-2 Wastewater Genomic Surveillance: Approaches, Challenges, and Opportunities. arXiv 2023;.
18. Gregory DA, Wieberg J C G adn Wenzel, Lin CH, Johnson MC. Monitoring SARS-CoV-2 Populations in Wastewater by Amplicon Sequencing and Using the Novel Program SAM Refiner. Viruses 2021;13(8).
19. Barbé L, Scaheffer J, Besnard A, Jousse S, Wurtzer S, Moulin L, et al. SARS-CoV-2 whole-genome sequencing using Oxford Nanopore Technology for variant monitoring in wastewaters. Frontiers in Microbiology 2022;p. 1362.
20. Agrawal S, Orschler L, Tavazzi S, Greither R, Gawlik BM, Lackner S. Genome Sequencing of Wastewater Confirms the Arrival of the SARS-CoV-2 Omicron Variant at Frankfurt Airport but Limited Spread in the City of Frankfurt, Germany, in November 2021. Microbiology Resource Announcements 2022;11(2):e01229–21.
21. Karthikeyan S, Levy JI, De Hoff P, Humphrey G, Birmingham A, Jepsen K, et al. Wastewater sequencing reveals early cryptic SARS-CoV-2 variant transmission. Nature 2022;609(7925):101–108.
22. Pechlivanis N, Tsagiopoulou M, Maniou MC, Togkousidis A, Mouchtaropoulou E, Chassalevris T, et al. Detecting SARS-CoV-2 lineages and mutational load in municipal wastewater and a use-case in the metropolitan area of Thessaloniki, Greece. Scientific reports 2022;12(1):1–12.
23. Valieris R, Drummond RD, Defelicibus A, Dias-Neto E, Rosales RA, Tojal da Silva I. A mixture model for determining SARS-Cov-2 variant composition in pooled samples. Bioinformatics 2022;38(7):1809–1815.
24. Ellmen I, Lynch MD, Nash D, Cheng J, Nissimov JI, Charles TC. Alcov: Estimating Variant of Concern Abundance from SARS-CoV-2 Wastewater Sequencing Data. medRxiv 2021;.
25. Barker DO, Buchanan CJ, Landgraff C, Taboada EN. MMMVI: Detecting SARS-CoV-2 Variants of Concern in Metagenomic Wastewater Samples. bioRxiv 2021;.
26. Schumann VF, de Castro Cuadrat RR, Wyler E, Wurmus R, Deter A, Quedenau C, et al. SARS-CoV-2 infection dynamics revealed by wastewater sequencing analysis and deconvolution. Science of The Total Environment 2022;853:158931.
27. Gafurov A, Baláž A, Amman F, Boršová K, Čabanová V, Klempa B, et al. VirPool: model-Based Estimation of SARS-CoV-2 Variant Proportions in Wastewater Samples. BMC Bioinformatics 2022;23(551). https://doi.org/10.1186/s12859-022-05100-3.
28. Posada-Céspedes S, Seifert D, Topolsky I, Jablonski KP, Metzner KJ, Beerenwinkel N. V-pipe: a computational pipeline for assessing viral genetic diversity from high-throughput data. Bioinformatics 2021;37(12):1673–1680.
29. Baaijens JA, Zulli A, Ott IM, Nika I, van der Lugt MJ, Petrone ME, et al. Lineage abundance estimation for SARS-CoV-2 in wastewater using transcriptome quantification techniques. Genome Biology 2022;23(1):1–20.
30. Korobeynikov A. wastewaterSPAdes: SARS-CoV-2 strain deconvolution using SPAdes toolkit. bioRxiv 2022;https://doi.org/10.1101/2022.12.08.519672.
31. Kayikcioglu T, Amirzadegan J, Rand H, Tesfaldet B, Timme RE, Pettengill JB. Performance of methods for SARS-CoV-2 variant detection and abundance estimation within mixed population samples. PeerJ 2023;11:e14596.
32. Bray NL, Pimentel H, Melsted P, Pachter L. Near-optimal probabilistic RNA-seq quantification. Nature biotechnology 2016;34(5):525–527.
33. Sutcliffe SG, Kraemer SA, Ellmen I, Knapp JJ, Overton AK, Nash D, et al. Tracking SARS-CoV-2 variants of concern in wastewater: an assessment of nine computational tools using simulated genomic data. bioRxiv 2023;p. 2023–12.

34. Turakhia Y, Thornlow B, Hinrichs AS, De Maio N, Gozashti L, Lanfear R, et al. Ultrafast Sample placement on Existing tRees (UShER) enables real-time phylogenetics for the SARS-CoV-2 pandemic. Nature Genetics 2021;53(6):809–816.
35. Di Tommaso P, Chatzou M, Floden EW, Barja PP, Palumbo E, Notredame C. Nextflow enables reproducible computational workflows. Nature biotechnology 2017;35(4):316–319.
36. rki-mf1, VLQ-nf. GitHub; 2021. https://github.com/rki-mf1/VLQ-nf.
37. Agrawal S, Orschler L, Zachmann K, Lackner S. Comprehensive mutation profiling from wastewater in southern Germany extends evidence of circulating SARS-CoV-2 diversity beyond mutations characteristic for Omicron. FEMS Microbes 2023 03;4.
38. Gangavarapu K, Latif AA, Mullen JL, Alkuzweny M, Hufbauer E, Tsueng G, et al. Outbreak.info genomic reports: scalable and dynamic surveillance of SARS-CoV-2 variants and mutations. Nature Methods 2023;20(4):512–22.
39. outbreak.info SARS-CoV-2 data explorer;. https://outbreak.info/, accessed: June 03, 2022.
40. Hadfield J, Megill C, Bell SM, Huddleston J, Potter B, Callender C, et al. Nextstrain: real-time tracking of pathogen evolution. Bioinformatics 2018;34(23):4121–4123.
41. Nextstrain;. ttps://nextstrain.org/, accessed: June 03, 2022.
42. Munteanu V, Gordeev V, Saldana M, Aßmann E, Su JM, Drabcinski N, et al., A rigorous benchmarking of methods for SARS-CoV-2 lineage abundance estimation in wastewater. arXiv;. Preprint arXiv:2309.16994. 29 Sep, 2023.
43. McBroome J, de Bernardi Schneider A, Roemer C, Wolfinger MT, Hinrichs AS, O'Toole AN, et al. A framework for automated scalable designation of viral pathogen lineages from genomic data. Nature Microbiology 2024;p. 1–11.
44. Abdeldayem OM, Dabbish AM, Habashy MM, Mostafa MK, Elhefnawy M, Amin L, et al. Viral outbreaks detection and surveillance using wastewater-based epidemiology, viral air sampling, and machine learning techniques: A comprehensive review and outlook. Science of The Total Environment 2022;803:149834.
45. Zhuang X, Vo V, Moshi M, Dhede K, Ghani N, Akbar S, et al. Early Detection of Novel SARS-CoV-2 Variants from Urban and Rural Wastewater through Genome Sequencing and Machine Learning. medRxiv 2024;p. 2024–04.
46. Ellmen I, Overton AK, Knapp JJ, Nash D, Ho H, Hungwe Y, et al. Learning novel SARS-CoV-2 lineages from wastewater sequencing data. ResearchSquare 2024;https://doi.org/10.21203/rs.3.rs-4159693/v1, preprint.
47. ThermoFisher Scientific, SARS-CoV-2 Research Using the GeneStudio S5 System;. https://www.thermofisher.com/de/de/home/life-science/sequencing/dna-sequencing/microbial-sequencing/microbial-identification-ion-torrent-next-generation-sequencing/viral-typing/coronavirus-research/genestudio-s5-system.html, accessed: July 01, 2024.
48. Cingolani P, Platts A, Wang LL, Coon M, Nguyen T, Wang L, et al. A program for annotating and predicting the effects of single nucleotide polymorphisms, SnpEff: SNPs in the genome of *Drosophila melanogaster* strain w1118; iso-2; iso-3. Fly 2012;6(2):80–92.
49. lifehashopes, MAMUSS. GitHub; 2022. https://github.com/lifehashopes/MAMUSS.
50. baymlab, VLQ: Viral Lineage Quantification. GitHub; 2021. https://github.com/baymlab/wastewater_analysis, accessed: commit hash 61dd29df* (September 16, 2021).
51. Aßmann E, Agrawal S, Laura O, Böttcher S, Lackner S, Hölzer M, Impact of reference design on estimating SARS-CoV-2 lineage abundances from wastewater sequencing data. OSF; 2023. doi:10.17605/OSF.IO/UPBQJ.
52. Eva A, Laura O, Martin H, Shelesh A, Sindy B, Susanne L, Supporting data for "Impact of reference design on estimating SARS-CoV-2 lineage abundances from wastewater sequencing data". GigaScience Database; 2024. http://gigadb.org/dataset/102547.

Supplement

Alternative allele frequency and size of reference database impact the *sequence-based* method but the effects are dependent on sample composition

Pan-EU-GER

Across all samples and experiments, we found the predictions of the *sequence-based* method to reflect the pandemic background in Germany well. Alpha and its sub-lineages were among the most prominent predictions within the time frame of wastewater sampling (Supplementary Figure S3). For most samples, we found Alpha and Q.1 to be the most abundant (sub-)lineages. The *sequence-based* method predicted distinctly varying abundances for sub-lineages other than Alpha, Beta, Gamma, or B.1.617 (summarized as "Other") across the *Pan-EU-GER* samples. We chose a cutoff of 1 % abundance to differentiate true positive predicted lineages from false positive noise. On average, we found the pseudo-alignment-based approach to detect around 20–30 % abundance of noise across all samples and parameter settings. At the minimum reference size (Supplementary Table S1), we observed for some samples a slightly decreasing amount of noise and a slightly increasing abundance for Alpha sub-lineages and "Others" when increasing the AAF threshold (e.g., sample INF_21051_D). We found, that the number of “Others” sub-lineages above 3 % abundance decreased with increasing reference size. Across all experiments, we found the sample INF_21011_D to be the only one to be predicted with one or two “Others” sub-lineages of at least 3 % abundance.

With increasing AAF threshold, we found distinct shifts in the estimated abundances for B.1.1.7 and Q.1. We observed those shifts to behave complementary but not consistently across all reference sizes: At the minimum reference size, we observed Alpha abundance predictions to distinctly increase and Q.1 abundances to decrease across all samples with increasing AAF threshold. Conversely, for reference size 5, we found Alpha abundance predictions to first increase and then decrease again with increasing AAF threshold. Vice versa, we observed Q.1 abundances to decrease and then increase again. At the largest reference size of 20 sequences per lineage, we observed a consistent decrease in Alpha abundance estimates and a consistent increase in Q.1 abundance estimates with increasing AAF threshold. Furthermore, we found abundances of other Alpha sub-lineages like Q.4 and Q.6 to also increase and decrease across varying parameter settings without following a clear pattern, but found the predicted abundances to not change as distinctly.

Overall, we found the performance of the *sequence-based* method to be mostly robust with varying settings for the AAF threshold and reference size. We observed the impact of those parameter changes to be stronger for more closely related lineages in a sample and in some cases to become weaker at larger reference sizes.

FFM-Airport

Across all parameter settings, the resulting abundance profiles for the *FFM-Airport* data set reflected the pandemic background in Europe and South Africa well around the time frame of wastewater sampling: the *sequence-based* method estimated Delta and its sub-lineages to represent the most abundant lineages and detected small proportions of Omicron (Supplementary Figure S4). We chose a cutoff of 1 % abundance to differentiate true positive lineages from false positive noise and labelled sub-lineages with a minimum abundance of 3 %. Because the *sequence-based* method detected Omicron sub-lineages at abundances below 3 %, the quantified levels are not labelled and due to the scale of Supplementary Figure S4 not visible. However, when grouped by parent lineage, the predicted Omicron proportions become obvious. On average, we found the *sequence-based* method to detect around 50 % abundance of noise across all parameter settings.

At the minimum reference size (SupplementaryTable S1), we observed a decreasing amount of low abundant noise with increasing AAF threshold. In contrast, with larger reference sizes, we found the amount of low abundant noise to change slightly and not follow a consistent pattern. Overall, we found the amount of noise to increase with increasing reference size. We observed the abundance estimates to increase for individual Delta sub-lineages with increasing AAF threshold. Specifically, we found the set of the most abundant Delta sub-lineages to change at every increase. Some examples for Delta sub-lineages that alternately were estimated among the most abundant lineages within a sample are AY.43.1 and AY.43.2, AY.43.3 and AY.42, and AY.121 and AY.122. When considering the lineage abundance profiles grouped by parent lineages, we found the predicted abundance profiles to not change distinctly across different parameter settings(Supplementary Figure S4.

Finally, we found different settings for the AAF threshold and reference size to not distinctly affect the performance of the *sequence-based* method. We observed variations in the abundance estimates among multiple Delta sub-lineages.

Supplementary Table S1. Table showing the minimum reference sizes across the different alternative allele frequency (AAF) thresholds considered in the parameter escalation experiments across our three benchmark data sets. Here, we list the minimum number of genome sequences required per lineage to capture every mutation with an AAF above the considered AAF threshold at least once based on the implemented sampling strategy during reference construction. The *Standards* reference database required the largest number of sequences to capture the predefined genomic variation. Overall, we observed that with an increasing AAF threshold, the minimum reference sizes per lineage decreased across all three benchmark data sets.

AAF threshold	Minimum number of sequences per lineage		
	Standards	Pan-EU-Ger	FFM-Airport
0.25	20	3	3
0.5	10	2	2
0.85	10	1	1

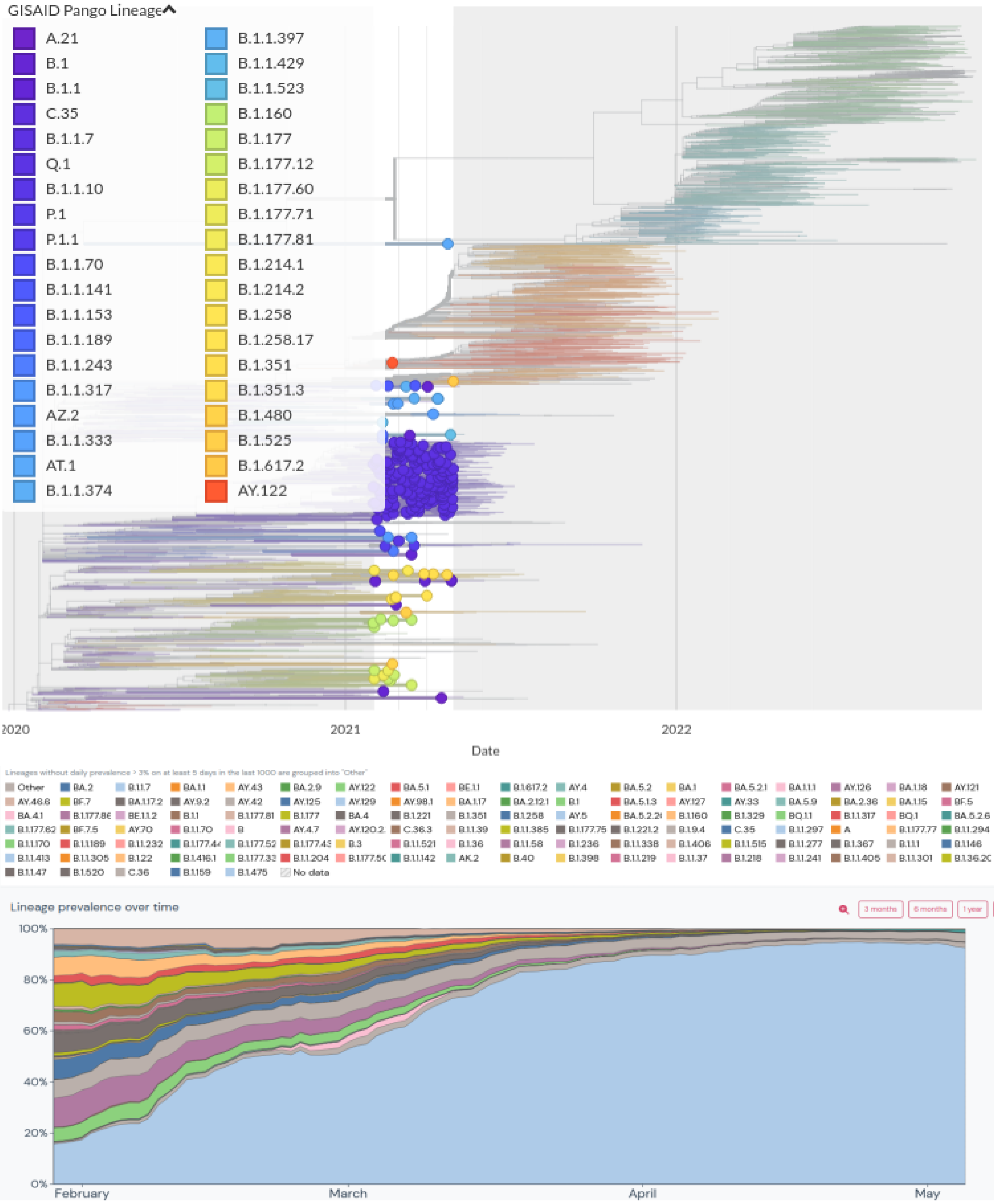

Supplementary Figure S1. Top: The pandemic background across Europe between 01 February and 30 April 2021 was built with Nextstrain.org. Bottom: The outbreak.org variant report for Germany displaying the SARS-CoV-2 lineage prevalence from February to March 2021 based on GISAID sequence data. The most dominant lineages in the plot from bottom to top: light blue = B.1.1.7, light green = B.1, purple = B.1.177.86, light grey = other, blue = B.1.258, dark grey = B.1.221, yellow = B.1.177, orange = B.1.160, light brown = B.1.177.81

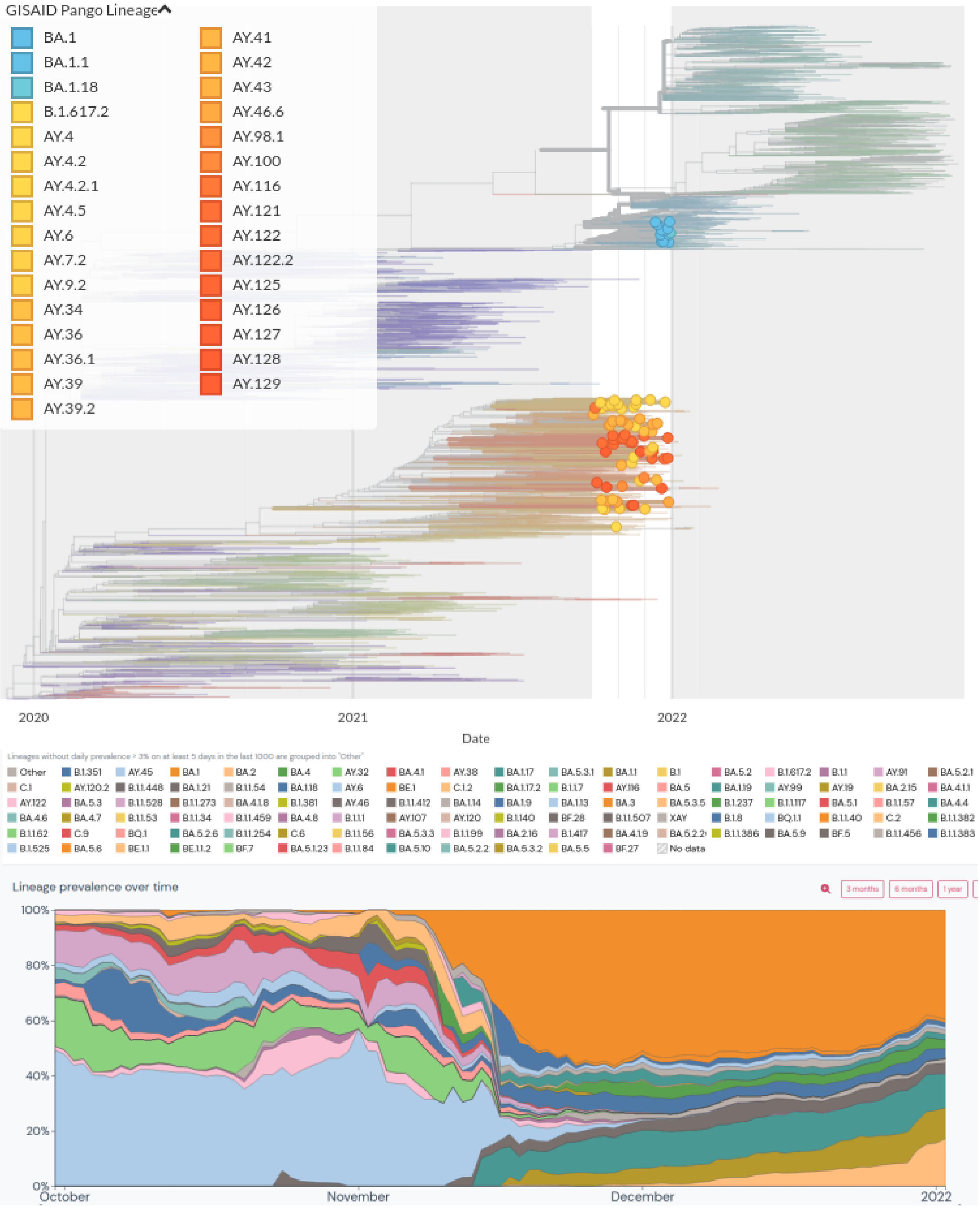

Supplementary Figure S2. Top: The pandemic background across Europe between 01 October and 31 December 2021 was built with Nextstrain.org. Bottom: The outbreak.org variant report for South Africa displaying the SARS-CoV-2 lineage prevalence from October to December 2021 based on GISAID sequence data. The most dominant lineages comprise sub-lineages of Delta and Omicron, but also B.1.351 (light green) and C.2 (light orange).

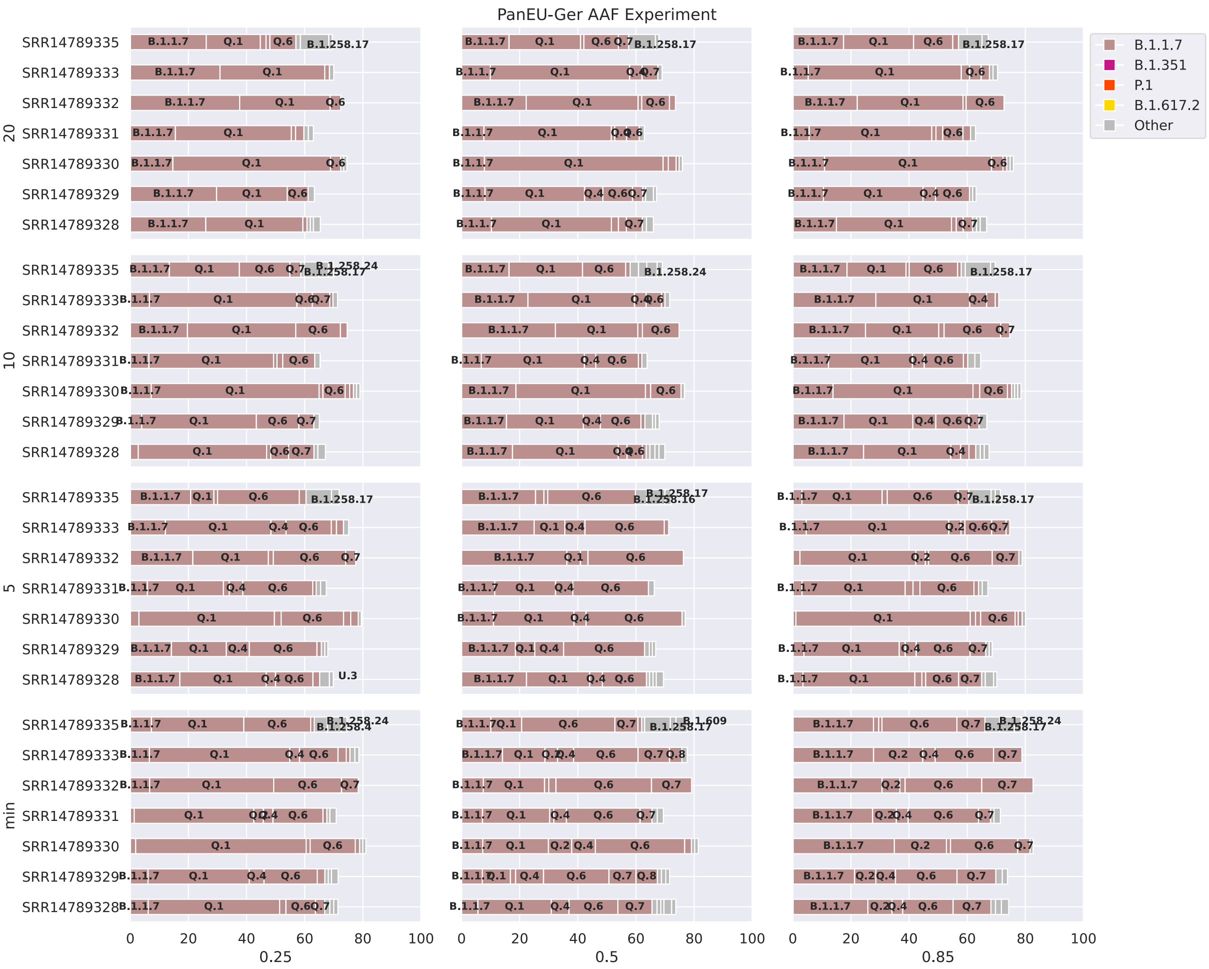

Supplementary Figure S3. Results for the parameter escalation experiments on the *Pan-EU-GER* samples using the *sequence-based* method using pseudo-alignment implementation. We analyzed the data set with different parameterization for reference construction (x-axis: increasing AAF threshold, y-axis: increasing maximum number of sequences per lineage). Abundance predictions are displayed at a minimum threshold of 1 % and labelled at a threshold of 3 %. When comparing with the pandemic background at the time of wastewater sampling, we observed the AAF threshold and the maximum number of sequences per lineage to impact the abundance proportions among Alpha and Q.1 the most. With more sequences per lineage in the reference, we found the impact of the AAF filter on the observed ambiguities to decrease. We found more low abundant sub-lineages predicted in the real wastewater data compared to the *Standards* data set and found those low abundant predictions to mostly not change distinctly across varying parameterization.

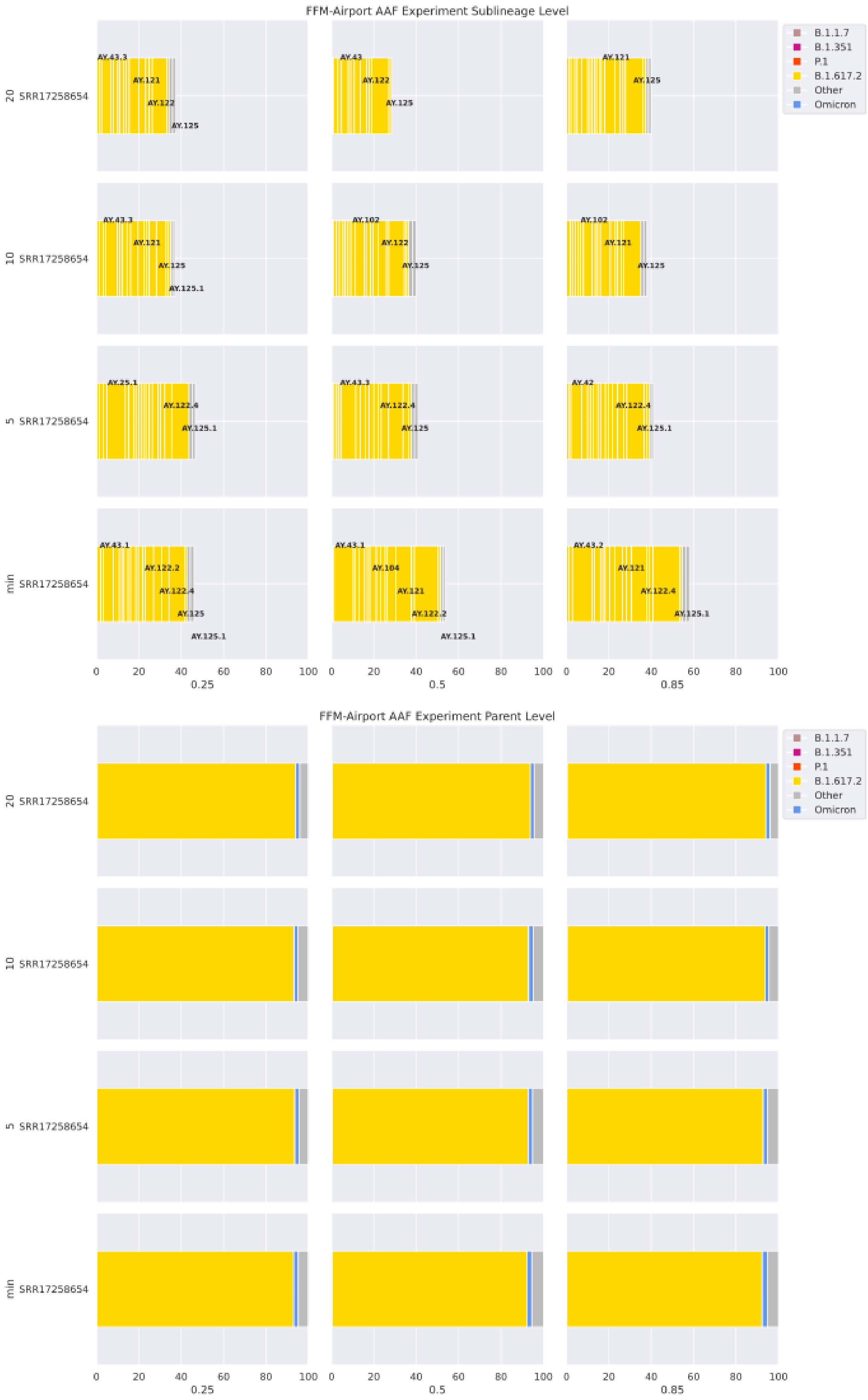

Supplementary Figure S4. Results for the parameter escalation experiments on the *FFM-Airport* data set using the *sequence-based* method. We analyzed the data set with different parameterization for reference construction (x-axis: increasing AAF threshold, y-axis: increasing maximum number of sequences per lineage). **Top**: Abundance predictions are displayed at a minimum threshold of 1 % abundance and labelled at a threshold of 3 % abundance. When comparing with the pandemic background at the time of wastewater sampling, we observed the following: Overall, we found more sub-lineages predicted with abundance below 1 % compared with the *Standards* data set and the *Pan-EU-GER* set. The *sequence-based* method detected more low abundant sub-lineages with increasing reference size and slightly less low abundant sub-lineages with increasing AAF threshold. Both the AAF threshold and the reference size showed to impact lineage ambiguities among Delta sub-lineages. **Bottom**: All abundance predictions are displayed as grouped by their parent lineage. We did not find the abundance predictions for parent lineages to change distinctly across experiments.

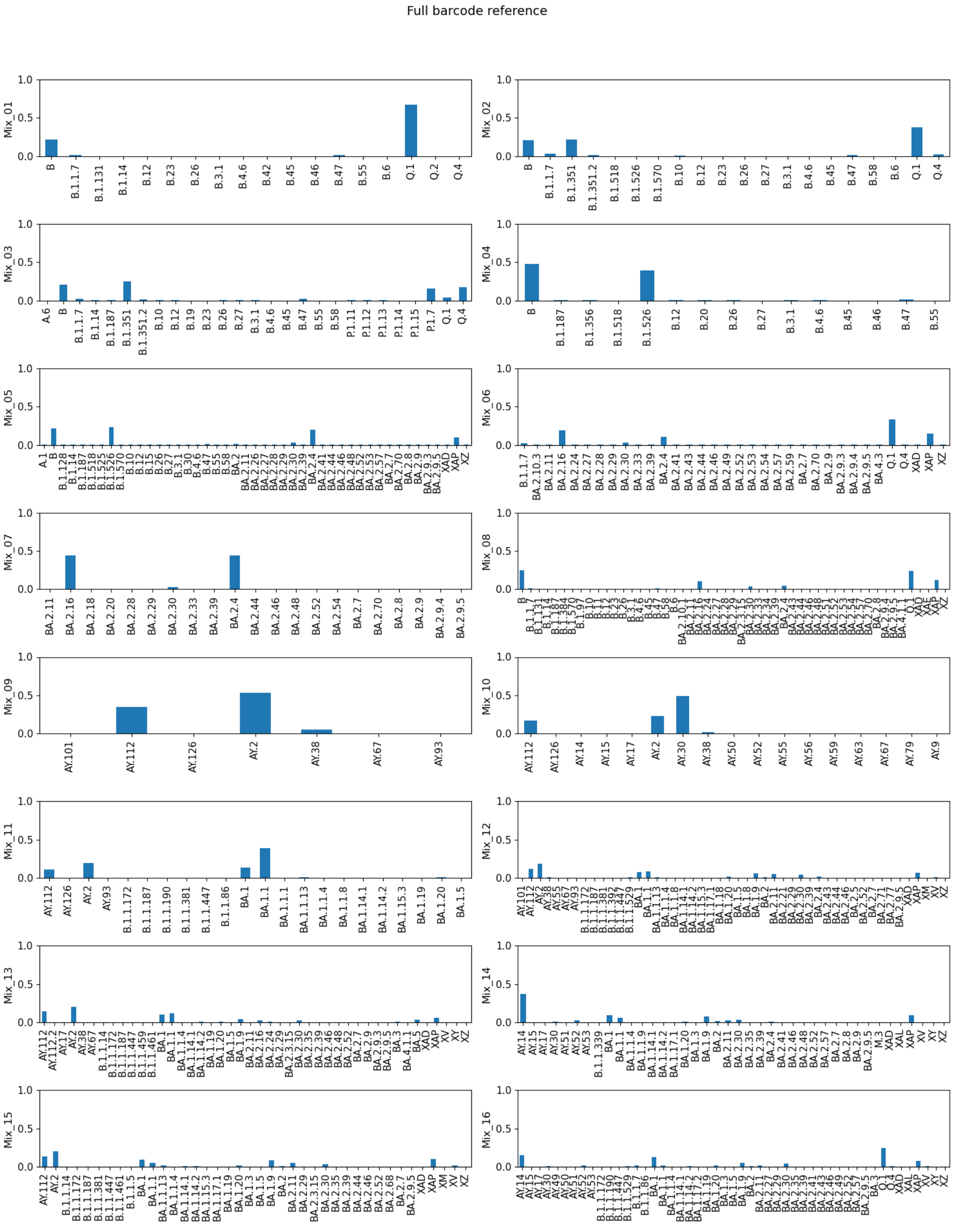

Supplementary Figure S5. SARS-CoV-2 lineage abundance assignments via Freyja [21] (v1.3.12) for the *Standards*. We used the full reference UShER set as provided as a default by the tool. In this case, multiple sub-lineages were predicted and frequencies were distributed among them, resulting in a reduced frequency estimate for the true (parental) lineage and an increase in low-frequency detections. For example, in Mix_07 the sub-lineages BA.2.16 and BA.2.4 were predicted with almost 50 %, respectively, while the included lineage BA.2 was not assigned (compare Figure S6).

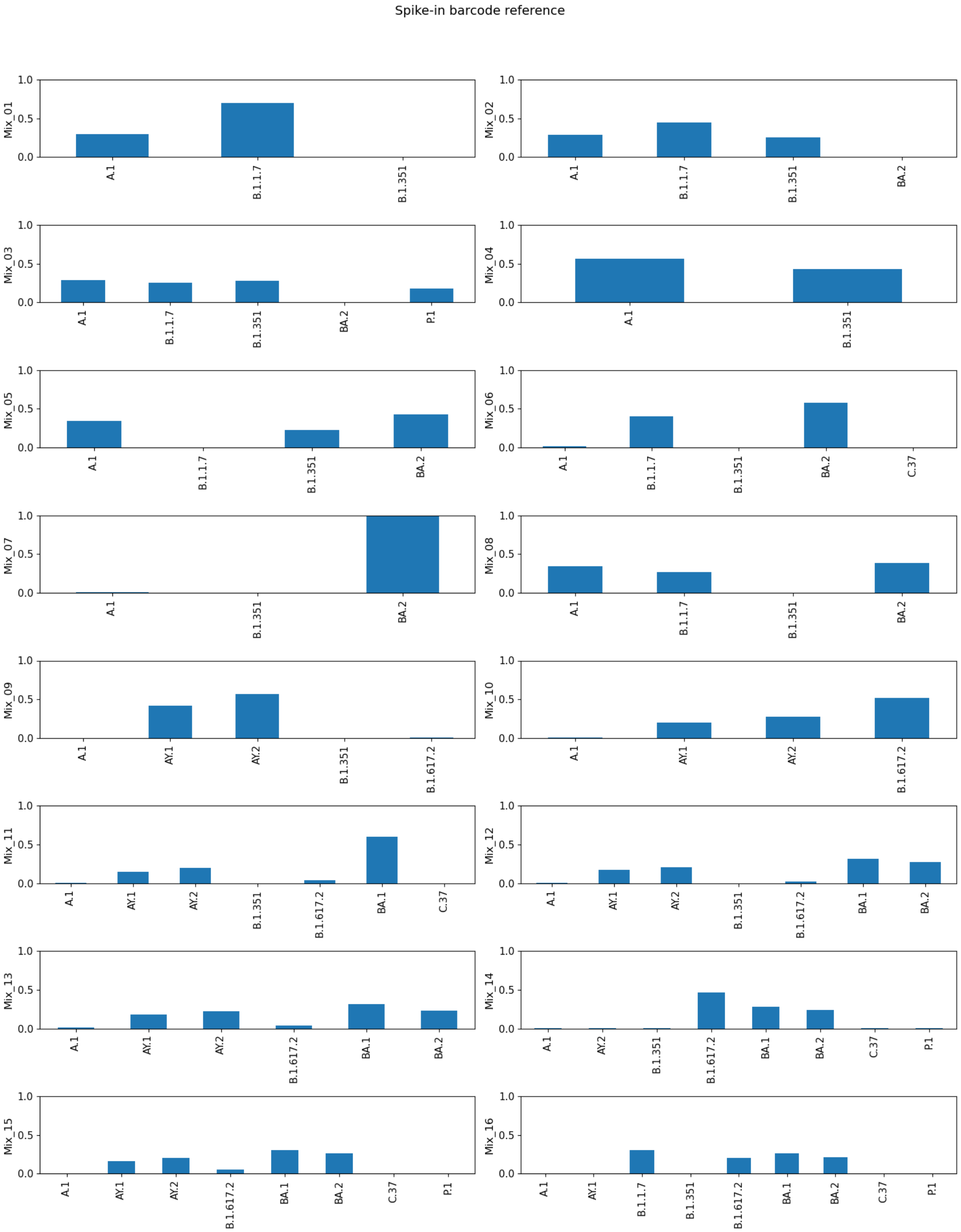

Supplementary Figure S6. SARS-CoV-2 lineage abundance assignments via Freyja [21] (v1.3.12) for the *Standards*. We reduced the reference UShER set to the lineages part of our artificial mixtures, instead of using the full UShER barcode data set as shown in Figure S5.

Supplementary Table S2. Table summarizing the mapping of each *Standards* sample.

SampleID	Total number of reads	Number of mapped reads	Average target base coverage depth*
Mix_01	11585401	11409164	67129
Mix_02	8000781	7872877	45648
Mix_03	7182909	7082168	41674
Mix_04	11156327	11033124	65477
Mix_05	9509819	9358747	54475
Mix_06	12228003	12005442	69829
Mix_07	11227258	10999971	63366
Mix_08	7991289	7886999	45904
Mix_09	22189148	21855903	21855903
Mix_10	8685555	8614170	8614170
Mix_11	2564976	2535182	2535182
Mix_12	2581594	2557744	2557744
Mix_13	8429568	8295217	8295217
Mix_14	6246713	6173867	6173867
Mix_15	11888445	11752739	11752739
Mix_16	6139010	6092648	6092648

*Target sequence was the SARS-CoV-2 reference genome (Wuhan-Hu-1)

FigureS1

Click here to access/download
Supplementary Material
FigureS1_Supplement.png

FigureS2

Click here to access/download
Supplementary Material
FigureS2_Supplement.png

FigureS3

Click here to access/download
Supplementary Material
FigureS3_Supplement.pdf

FigureS4

Click here to access/download
Supplementary Material
FigureS4_Supplement.png

FigureS5

Click here to access/download
Supplementary Material
FigureS5_Supplement.png

FigureS6

Click here to access/download
Supplementary Material
FigureS6_Supplement.png

Personal Cover Click here to access/download;Personal Cover;2024-04-30-response.pdf

Revision #1

Impact of reference design on estimating SARS-CoV-2 lineage abundances from wastewater sequencing data (GIGA-D-23-00161)

Eva Aßmann; Shelesh Agrawal; Laura Orschler; Sindy Böttcher; Susanne Lackner; Martin Hölzer

Dear Dr. Zhou, Dear reviewers,

Thank you again for handling our manuscript titled “Impact of reference design on estimating SARS-CoV-2 lineage abundances from wastewater sequencing data" (GIGA-D-23-00161). We appreciate the constructive comments of the two reviewers and are pleased to attach the revised version of the manuscript along with our detailed responses to the reviewers' comments. Please apologize for the long table in response to Rev #1: we wanted to document all changes and comment on all questions so thoughtfully raised via comments in the PDF version of our manuscript.

Most importantly, as the reviewers highlighted, we have clarified the scope of our study and added more information on potential limitations in the main manuscript. This addition is crucial for the accurate interpretation of our results. We have also refined the use of statistical tests and adjusted the corresponding language throughout the manuscript to ensure clarity and precision.

In response to the reviewers' feedback, we have made several significant changes:

1. We expanded the discussion on the challenges of reconstructing full genome sequences from wastewater data, acknowledging the inherent limitations due to RNA degradation and the presence of mixed viral populations. The revised manuscript details this, providing a clearer understanding of the constraints faced during data analysis. However, please also note that we don't reconstruct any genomes from wastewater data—we also clarified that.
2. We harmonized the terminology used throughout the manuscript to avoid confusion between 'variants' and 'lineages', and addressed all grammatical and repetitive sentence concerns pointed out by Reviewer #1.
3. We revised sections where terms like "significant" were used without statistical tests, ensuring that all claims are now supported by appropriate statistical analysis or are rephrased to reflect the observational nature of the findings.
4. Each point raised by the reviewers has been comprehensively addressed, ensuring no query was left unanswered. Our responses are detailed in the attached document.

We believe these revisions have significantly strengthened our manuscript, making the findings more robust, transparent, and useful for the field. We are grateful for the opportunity to enhance our work based on the insightful feedback from the review process.

We look forward to the possibility of our study being published in GigaScience and believe it will make a valuable contribution to the ongoing efforts in understanding and utilizing wastewater sequencing data for public health surveillance.

Thank you for considering our revised manuscript. We are eager to see it contribute to the scientific community and help advance our understanding of SARS-CoV-2 dynamics in wastewater-based epidemiology.

Best,

Martin Hölzer
(on behalf of all co-authors)

Reviewer #1

Dear all,

Please find attached my comments and suggestions to the manuscript. In the manuscript "Impact of reference design on estimating SARS-CoV-2 lineage abundances from wastewater sequencing data" Aßmann et. al compare two methods, a sequence and mutation-based, respectively, to better understand the circulating lineages and sub-lineages in wastewater samples. Since the advent of wastewater-based epidemiology (WBE) as a tool to complement results from clinical data, there has been search for novel tools that can give robustness to the results and more importantly confidence in the data analysis. In this context, this manuscript is very important as it is contributing towards achieving that goal. This is clear in the fact that they have designed a new tool, namely MAMUSS.

Q: 1. One aspect however that the manuscript fails to mention is the difficulty in reconstructing full genome sequences from wastewater data. This has been one of the biggest problems since it is widely accepted that viral particles in water do degrade, and consequently what is being sequenced is a partial genome. Consensus sequences are therefore very difficult to obtain.

A: Thanks for the comment; we fully agree. Degradation of the RNA genome of SARS-CoV-2 viral particles is a challenge, especially in wastewater samples consisting of viral RNA from many individuals. In the same context, degradation poses a challenge, as does the mixture of different SARS-CoV-2 lineages within one sample. Thus, the presence of fragmented SARS-CoV-2 RNA from different virus variants makes it challenging, if not impossible, to reliably reconstruct a complete genome of each SARS-CoV-2 virus variant present in a wastewater sample. Generating a consensus sequence based on the mapped reads and called mutations - like it is standard when sequencing patient samples - will result in a chimeric consensus representing a mixture of different SARS-CoV-2 lineages or representing only the most dominant lineage within a mixed wastewater sample.

However, it is possible to get a high “horizontal” genome coverage from wastewater samples and, based on that, a nearly complete consensus genome sequence (but which is based on the most abundant nucleotides found at each position while mapping the amplicon reads to the reference genome sequence, as mentioned above). Thus, in our opinion, we need to distinguish between reconstructing a consensus genome sequence representative for a single SARS-CoV-2 (sub)lineage - which is very difficult from wastewater samples - or reconstructing a consensus genome sequence representing the most abundant variant calls (mutations, INDELs) for a wastewater sample (possible, and also done in the community and uploaded to GISAID; although it is debatable how useful and informative such consensus sequences are).

In this context, please note that we never aimed to reconstruct a consensus sequence in our manuscript — for the same reasons you mentioned and we described here. Please compare L68-L74, where we mention the challenges of recovering all SARS-CoV-2 genomes from a wastewater sample. We also extended this paragraph to make the consensus reconstruction challenge clearer.

Q: 2. Another aspect that the authors fail to mention in the introduction or as a point of discussion, is how a variant is defined and how we take this information from clinical samples to adopt it then to define variants in environmental samples, although some relevant tools are mentioned such as COJAC and MMMVI. Yet, how these are used, it is not explained.

A: Thanks; we agree that it is important to define and distinguish mutation and virus variants clearly and better explain which information is utilized from clinical settings for wastewater surveillance. However, please also note that we don't define any virus variants or lineages; we use the established definitions from the community. We have updated the first paragraph (L21-29) in the introduction to reflect better and explain how the information about clinically defined SARS-CoV-2 variants is used for wastewater analysis. The important point is that virus variants are defined based on their mutational profile (and additional epidemiological and geographic factors, such as in the Pangolin system) derived from sequencing patient samples. The corresponding virus variant "label" (Pangolin, Nextclade, WHO, …) together with the mutational profile, can then be used to search for mutation patterns in a wastewater sample to assign a virus variant name. In the same context, we also mention tools specifically used for the wastewater genome sequencing analysis and SARS-CoV-2 lineage decomposition. Please also note, as detailed below again, that our focus was to compare the general approaches of mutation- and sequence-based reference construction and lineage abundance estimation and not compare specific tools. We believe it is important to form a foundation for robust reference set definitions and then investigate specific tools in more detail. We are doing this right now in a larger consortium, and a preprint will be available soon as a follow-up of this study.

Q: 3. The manuscript is well written, there are some repetitive sentences that need to be removed (see comments on PDF) as well as a couple of sentences which are not grammatically correct (see comments on PDF).

A: Thanks for the thoughtful comments in the PDF. We corrected them accordingly. Please see the table below for an overview of the changes we made and our corresponding comments.

Q: 4. It is worth mentioning that the words "variants" and "lineages" are used interchangeably. I do suggest they choose one term only.

A: Thanks; we fully agree and harmonize the usage of the terms. If we see a reason to still use the term "variant", we write more precisely "virus variant" or "SARS-CoV-2 variant" to also distinguish from mutational variation ("variant calling").

Q: 5. The manuscript mentions several times the presence of false and true positive, however does not mention how these were calculated. These need to be supported by a small statistical test.

A: Yes, we fully agree. When we use "significant", we also need a test. We changed the wording accordingly when we did not perform a statistical test. Regarding FP/TP, we are using the terms for (sub)lineages of which we know that they must be in our mixture sample (TP, when detected), or we use FP when a (sub)lineage is detected that can not be part of our mixture by design. We added a definition to the manuscript to clarify this (see lines 233-239).

Q: 6. There are minor corrections throughout the manuscript that need to be addressed. All these are highlighted as comments in the original manuscript.

A: Thanks. We checked all detailed PDF comments. Please see the table below for an overview of the changes and our comments. All changes are also marked in the new manuscript text (blue color).

Addressing comments in the PDF:

**position in the submitted manuscript**	**Marked text**	**Comment**	**Our answer**	**Status**
Abstract	2) German samples from early 2021	Are these clinical or wastewater?	2) German wastewater samples from early 2021	changed
Abstract	3) samples obtained from wastewater at an international airport in Germany from the end of 2021, including first signals of Omicron.	Does this imply that in Germany the variants skipped the Delta surge for example, which was dominant around the world before Omicron?	Indeed, the selected airport ww samples mainly contain Delta as expected (see Results), but were chosen for analysis because they already contained low signals of imported Omicron cases. We wanted to test both approaches (mutation- and sequence-based reference construction for the estimation of the SARS-CoV-2 abundance in wastewater samples) for the ability to detect signals of very low abundant new sub-lineages among circulating known/dominant	answered

			lineages, which is why these samples specifically were well suited for our experiments.	
86	VOC	define	Already defined in line 59	answered
148-155		repetitive	We agree that we repeat ourselves a few times in the last few Background paragraphs [115 ff.]. We generally removed repetitive paragraphs and only kept them in reduced versions when we found it stylistically necessary to tell the story.	Done
171	real samples	rephrase	wastewater samples	changed
177	n=1 sample	not statistically significant	Yes, we agree. However, and as explained in the manuscript, we want to show this sample as a proof-of-concept when the first Omicron variants were arriving in Germany. Thus, this sample also poses a particular challenge to detect Omicron in a huge Delta background. Also based on your other comment, we checked the text again and made sure that	answered

			we don't speak about "significant" in such a context when sample number is low or we don't provide a statistical test.	
181 f.	Please note that no real wastewater was used to construct the Standards (see Methods)	delete sentence	That is true, we made it more clear that no real WW was used for the spike-in mixtures.	Done
189 f.	Lastly, we obtained one sample (SRR172….) from	one seems statistically insignificant. if cannot use more than one, explain	Yes, we agree. However, and as explained in the manuscript, we want to show this sample as a proof-of-concept when the first Omicron variants were arriving in Germany. Thus, this sample also poses a particular challenge to detect Omicron in a huge Delta background. Also based on your other comment, we checked the text again and made sure that we don't speak about "significant" in such a context when sample number is low or we don't provide a statistical test.	answered
209 ff.	SARS-CoV-2 variants in wastewater samples are determined by	what variant caller	We agree and this is more detailed in the Methods. Hence, we decided to	changed

	comparing the mutations profiles, generated using a variant caller,....		delete this subsection and describe the methodological details only in the "Methods" section. For example, we write "using Variant Caller (v5.12.0.4) with "Generic - S5/S5XL (510/520/530) - Somatic - Low Stringency" default parameters."	
214 ff.	For the sequence-based approach, we implemented …..	move to methods	We agree that this is Methods and removed the paragraph from the Data section.	changed
226	The abundance estimation is performed by an Expectation-Maximization algorithm	reference and explanation in methods	Thanks, we agree and like above removed that part from the Data section, because it is detailed in the Methods. Additionally, we decided to remove the following Data availability subsection, because it repeated information that was already provided in the Data availability sections at the end of the manuscript.	changed
245	We analyze our Standards data set …	synthetic samples	It's correct that we refer to the synthetic data set.<br>Because we use	answered

			it as a standard to level the comparison between both approaches, we would like to stick to the name that represents the experimental purpose of the samples.	
255	We observed the most consistent false positive estimations ….	how you calculate these?	In the context of our study, we define “false positive” as a lineage that was detected based on a sample’s sequencing data despite it not being spiked into the synthetic mixture.<br>We added a definition of FPs and FNs accordingly in line 233-239	changed
258		reviewer remove “also”	accepted	changed
258 f.	false positives in the sample	how are false positives calculated	We identify a lineage as false positive by comparing all predicted lineages against the ground truth composition of our spiked standard sample. All lineages that do not occur in the ground truth composition are marked as false positives.<br>We added a definition of FPs and FNs in line 233-239	changed

266	…the mutation-based approach could not detect Iota (B.1.526) but falsely detected BA.1	are mutations in common, how many?	In the scope of this study, we considered the final output of the evaluated deconvolution tools, i.e. lineage abundance profiles. We agree that inspecting each tool's lineage assignment on a sequence/mutation level would be useful to understand the biases of each tool and identify genomic regions with a high ambiguity for lineage assignment tasks. However, the aim of this study was to focus on the more general impact of using different reference data types for lineage abundance estimation.<br><br>We decided to rephrase this and similar observations, such that we describe undetected lineage without speculating about the lineages that have been detected instead as we do not evaluate this by inspecting (mis-)matching mutation/sequence patterns.	changed and answered

273 f.	….both approaches falsely detected Delta while underestimating AY.1 or AY.2..	explain what are the differences at mutational level	We agree that inspecting each tool's lineage assignment on a sequence/mutation level would be useful to understand the biases of each tool and identify genomic regions with a high ambiguity for lineage assignment tasks. However, the aim of this study was to focus on the more general impact of using different reference data types for lineage abundance estimation.	answered
287 ff.		how you calculate false negatives?	Analogously to false positives, we define a lineage as false negative if it was spiked into the synthetic mixture of a sample, but was not detected on the sequencing data. We identify false negative lineages by comparing the predicted lineages against the ground truth sample composition. We added a definition of FPs and FNs in line 233-239	answered
297 ff.		this first paragraph is	For each Results section, we	answered

		mainly methods	aimed at including some short background information to guide the reader into the context of downstream described results. We think that is a question of writing style and how to tell the story in the paper. Thus, we would like to keep such short “connecting” text parts between main sections. However, we also agree that certain parts of our paper were too repetitive (like having methodological descriptions again in the Results) and resolved such parts.	
303	..we performed experiments on real data to evaluate…	none	In absence of a comment, we assume you commented on the use of the term “real data/wastewater” . We removed or replaced the term “real” at multiple locations	changed
309	…, the pandemic situation in Europe from February …		There was no comment so we were not sure what to change.	answered
313	The pandemic situation in Germany at that time was mainly	voc?	We added VOC and sorted the list of lineages	changed

	dominated by Alpha,....			
318	According to GISAID submissions during that time, approximately the same lineages and multiple other low-abundant global and European sub-lineages were reported from clinical sampling strategies.	reference	Thanks for catching this, we added a reference	done
Figure 2		omicron ba.2 was not circulating in late 2021	That is true. With the Standards dataset, we wanted to build a synthetic dataset for baseline comparison of both approaches. For that purpose, we designed different synthetic lineage compositions that did not necessarily aim at capturing realistic co-occurrences, but mainly at stimulating challenging conditions for lineage prediction and abundance estimation.	answered

Figure 2		1. invert as per figure<br>2. somewhere in the text you need to explain how mutations are used to assign variants	1. We would like to keep the plot as it is. Our motivation is to give a visual comparison of both approaches on a sample basis<br>2. We added more details about the general usage of mutations from clinical samples transferred to wastewater data to the Introduction. Further, we explain the two main approaches in the Methods . Also, please note the reference focus of our study. We keep the description of lineage assignment minimal and focus on	answered

			reference design impact.	

323 ff.		results start here - all of the above sounds more methods	We generally agree, but as explained above we would like to keep these connecting parts to remind the reader of our data sets and approaches and to guide our story. We hope that you are fine with this particular element of style.	answered
330	Yet, those Alpha sub-lineages were not reported amongst the most frequent cases based on clinical sampling strategies.	they don't have to be	In general, we agree. Just because sublineages were not reported from clinical cases at location X, this does not mean that they did not circulate in the wastewater of X. However, for Q.1 and Q.7 being so abundantly predicted in the wastewater it does not add up with the comparably very small proportion of clinical cases being reported for Germany (and german reporting was quite representative at that time with huge genomic surveillance efforts going on). Besides, we also need to consider the dynamic nomenclature system: Alpha sublineages were defined	done

			retrospectively and relatively late so it might be also possible that re-analyzing clinical data will now provide more Alpha sublineage (Q*) assignments. While this is out of scope of our study (and again highlights the reference bias), we extended the corresponding sentence to clarify this.	
345	…detect low abundant signals of Omicron	were both omicron sub lineages circulating back then? i think it's a bit early	Thanks, we are now more specific on the Omicron sublineages in the text.<br><br>With Omicron we refer to BA.1. During the time of sampling, as described in the text, only BA.1 and sublineages were circulating (BA.2 not yet detected).	done
358	we observed BA.1 with 1.44% and some other lineages and sub-lineages with abundances of less than 1% ("Other")	this does not mean it was an omicron sublineage. did you check when was the first time ba2 was detected?	changed the text to clarify	changed
376 ff.		where is the picture with these results?	We added references to the respective results in figure 4 and supplementary figure 2	changed

359 f.	…we performed parameter escalation experiments…	what is this?	We added a reference to Methods, where we describe our experimental setup.	done
400	supplement	number?	We added a reference to respective supplementary sections and figures	changed
401 ff.		is this result or method?	As described above, we would like to keep this part because we think it contributes to the understanding of our study and findings	answered
406	AAF		We could not access this comment, but we assume it concerns the definition of AAF, which is given in the text and in Methods.	answered
433 f	with varying parameter settings	what parameters?	We added some more explanation on the exact parameters that were explored here.	changed
Figure 3		x axis not defined	Thanks for catching this, we added an axis description to the caption.	done
452 f	with increasing reference size	genome size?	We rephrased this part to clarify that we refer to the number of reference genomes per lineage in the	changed

			reference data set	
477	5 sequences per lineage	how is this selection made?	We added the reasoning for choosing this setting out of the explored parameters in the following sentence.	changed
484	“It is apparent that the composition of the reference used must have a larger impact on the determination of relative SARS-CoV-2 abundances in wastewater sequence data”	it is not clear how the references were chosen	In the Methods section, we explained in detail how we chose the reference sets, e.g., circulating lineages based on GISAID data during the respective time frames. We extended that methodological part to make it clearer (see below).	done
528	The most remarkable difference was in the number of detected sub-lineages, which also directly correlates with the reference design	the reference design is very important	Yes, we agree	done
533 ff.	For the mutation-based approach and the implementation we used, it got increasingly difficult to select a representative set of marker mutations	aren’t these published?	We agree that the characteristic mutation profiles were published with each emerging variant. Or at least, one can derive characteristic mutations from clinical genome sequences per lineage or look	

			up the mutation profiles used to define a lineage. However, due to extreme overlaps, especially due to convergent evolution and more and more (sub)lineages being defined, it became increasingly challenging to (manually) select enough unique mutations among the 50 most prevalent mutations that were used as reference mutation set for calling a lineage via MAMUSS. This is the main point we wanted to make.<br><br>Apparently, this was not clear. For better clarity, we have added text on the selection of reference mutation sets in the methods, as commented by the reviewer in the previous comment. (L750-760)	
538	…of SARS-CoV-2 full genome sequences…	are wastewater full genome?	Thanks for catching this unclear statement. Here, we are not referring to wastewater sequencing data, but to SARS-	done

			CoV-2 genomes available on GISAID that were reconstructed from clinical data. We clarified this accordingly in the text.	
566	..sources for bias in their general behavior…	what behavior?	We removed the term “behaviors” and instead described the potential sources of bias we observed.	done
606 f.	..,low-frequency mutations might help better differentiate lineages.	This sentence does not seem correct. expand	We agree that this statement is not well connected to the preceding text, where we discuss the impact of parameter selection during reference reconstruction. We updated the text accordingly by giving a more elaborate description of our hypothesis.	done
609	Most importantly,...	this is not a correct way to start a sentence from beginning of paragraph - it seems more the continuation of previous	We agree and updated this section accordingly by embedding it into a now preceding paragraph	Done
655	…, the reference design must also be adjusted.	explain better	We updated the text accordingly to provide a more understandable explanation.	done
655 ff.	Otherwise, a	check	In the context of	done

	lineage defined with a delay….	grammar	the above comment, we rephrased this sentence	
669	The detection of cryptic (novel, undescribed)...	reference	Thanks for catching this position, we added the required reference.	done
682	..Synthetic mixture Standards	Call them either of these two definitions	We agree that the data set description is duplicated here. We removed “Synthetic mixture” and kept the name “Standards” to stay consistent throughout the manuscript.	done
692	near-full genome of SARS-CoV-2		We could not find a comment to reply to here.	done
693	We performed multiple sequencing runs	define how many	Thank you for pointing out. We have added information about the number of runs (2 runs).	done
702 ff.	Benchmark data set #2: Pan-EU-GER	this sentence needs to be re-written correctly as it does not reflect the beginning of a paragraph. as the one below it seems it has been copied and pasted from elsewhere	Thanks for the remark. Because we did not produce this data set, and because we already provided all necessary availability information on this data set in the Data section, we decided to remove this whole subsection.	changed

707 ff.	Benchmark data set #3: FFM-Airport	as above	As described above, we decided to remove this subsection, since all necessary information is already provided in the Data section.	changed
713	SARS-CoV-2 Research Plug-in Package	Is this available	Yes, it is an active plug-in used on ION_TORRENT. However, it is bound to the software/company, and thus, we can not provide any source code links. Details (version number) are provided in the Methods.	done
715	SARS_CoV_2_coverageAnalysis (v5.16)	reference or website	The coverage analysis is part of the plug-in suite of the Ion Torrent sequencer. We have now added the link to the website for the information. Please also note, that the plugin is cited in a similar way (if at all) in other publications, such as https://doi.org/10.1128/jcm.00649-21 and https://www.ncbi.nlm.nih.gov/pmc/articles/PMC9227152/	done
720	For mutation calls, …	too generic, please list	The specific plug-ins/tools used for variant calling are listed in the	done

			following sentences.	
730	…to reconstruct reference mutation…	repetitive	We agreed and updated the sentence accordingly.	Done
745 ff.	Here, the main idea is that quantification of different transcripts….	rephrase - I am not sure I understand since this is not an RNASeq experiment: there is no calculation of transcripts estimate	Exactly, there is no RNASeq involved, we are simply referring to a tool that repurposes a computational method that was developed to assign RNASeq data to transcripts. In this sentence we want to emphasize how the problem of assigning wastewater sequencing reads to their originating lineage genomes is computational very similar. Meaning, one can repurpose the tool (Kallisto), by replacing transcripts by lineage reference genomes and RNASeq data by wastewater sequencing reads.	Done
759 ff.	First, a reference data set …	the choice of these sequences is what will determine the success of the sequence based method. this should be	We agree. That is exactly one of the objectives of this study - To show the impact of the reference design on the analysis. This is, for example, mentioned in	Done

		mentioned somewhere in the text	lines 813-185 in the section "**Reconstruction of indices for the *sequence-based* approach**"	
775 ff.	…abundances are estimated similarly to …	rephrase to make it easy to understand as it creates confusion	We agree, mentioning the analogy of RNASeq analysis is more confusing than helpful here. We removed this part and replaced it with a more specific explanation of how Kallisto estimates lineage abundances using wastewater sequencing reads and the reconstructed reference data set.	Done
789	alternate allele frequency (AAF)	already defined	Thanks for catching this, we removed the definition here.	Done
805 f	…AAF filter passing mutations are captured at least…	repetitive as in line 792-793	We agree, our description of the adjustments we implemented are repetitive. Hence, we updated this paragraph aiming at a more understandable description.	Done
831	Now, we added	Replace now by then		changed
832	..the final reference set manually. Otherwise, the A.1 sequences	replace"manually. Otherwise" by "as otherwise"		changed
844 ff.	We did not only select …	this sentence is not	Thanks for catching this, we	done

		grammatically correct	corrected the sentence accordingly	
849	…sequences per lineage.	it is worth mentioning here that these GISAID references are from clinical cases. correct?	We agree, it might help to highlight that our reference data sets are built from clinical sequencing data. We added this information to the commented paragraph.	Done
852 f	..from European and South African samples…	does this mean flights from Europe and South Africa? if so, explain why. given the time frame and provenance of the Delta variants , other countries might have suited better.	Thanks for the remark. Indeed, this sentence required some clarification. We updated the sentence to emphasize that we filtered clinically derived genome sequences from GISAID records. With the FFM-Airport data set, we mainly wanted to focus on the potential to identify low abundant traces of a novel lineage (Omicron) amongst a noisy background of high and low-abundant Delta sub-lineages. We decided to compare the airport wastewater sequencing data with concurrent clinical sequencing data from South Africa and Europe to screen for	Done

			genomic signals of Omicron sublineages that might have been imported from SA (first observed clinical report) or other european countries where cases of Omicron sublineages were already reported before observed in Germany.	
921 ff.	However, …	This sentence is not grammatically correct	We rephrased the sentence	Done

Reviewer #2:

In this study, the authors initiate a novel exploration by employing parameter escalation experiments to assess the impact of reference size and alternative allele frequency cutoffs on the effects of virus lineage composition in wastewater samples and their references. The research provides valuable insights into how different parameter settings influence outcomes in test data sets, particularly highlighting the role of virus lineage composition in wastewater samples and the corresponding references. Detailed parameters for these analyses are made available in several bash files at osf.io/upbqj. Despite these significant contributions, certain areas could benefit from further enhancement:

Q: 1. The current methodology utilizes Ion Torrent for testing mock samples. However, this approach may not fully capture the variability in alignment and sub-lineage analysis. Incorporating additional sequencing data from PacBio, Nanopore, and Illumina would offer a more comprehensive examination of these aspects, potentially leading to more robust findings.

A: Thanks for the comment. We welcome the suggestion to include data from additional sequencing technologies such as PacBio, Nanopore, and Illumina. However, in our experience, sequencing technology does not greatly impact alignment and sublineage analysis variability - when the technology-specific sequence characteristics are considered, such as using specific tools for Nanopore variant calling. Besides, differences in the enrichment of genetic material from the wastewater matrix, choice of primer design and amplicon scheme, as well as the reference database used and the parameters in the bioinformatic analysis, are factors that are often neglected when the focus is on the choice of sequencing technology. But of course, we also agree that there are technology-specific

differences in sequencing options, such as INDELs at Nanopore [Delahaye et al. 2021, PloS One] or subtle differences between 2- and 4-color chemistry at Illumina [Stoler et al. 2021, NAR]. The same holds true for specific characteristics of Ion Torrent data [Bragg et al. 2013, PLoS Computational Biology]. However, we would like to clarify the scope and focus of our research to address this point.

Our study aimed to investigate the effects of reference sequence selection and parameter settings on estimating SARS-CoV-2 lineage abundance in wastewater sequencing data. The main objective was to demonstrate how the choice of reference databases and analytical parameters affects the results of such analyses. We deliberately chose Ion Torrent sequencing technology to demonstrate these effects in a specific, controlled context with which we have much experience.

The effects that different sequencing kits and platforms, including Ion Torrent, Nanopore, and Illumina, can have on alignment and sublineage identification variations due to their different error profiles and sequencing characteristics have already been investigated in various studies [Carbo et al. 2023, Eur J Clin Microbiol Infect Dis; Plitnick et al. 2021, J Clin Microbiol; von Sydow et al. 2023, Scientific Reports; Tshiabuila et al. 2022, BMC Genomics; Ramphal et al. 2023, Research Square]. Such differences are studied in the literature and contribute to a broader understanding of the performance of sequencing technologies in different applications, which is crucial when interpreting the results. However, our study does not aim to compare these technologies or benchmark the performance of sequencing platforms - although we fully agree that such investigations are likewise crucial - especially in environmental settings. Instead, we focus on the impact of reference database composition and analysis parameters on lineage abundance estimates - a topic that is of great importance regardless of the sequencing technology used, especially in the post-pandemic time with clinical sequencing going down and subsequent dilution of available reference sequences.

In addition, we selected Ion Torrent sequencing data for this study because our protocol, which has been optimized over the course of the pandemic [Agrawal et al. 2021, Sci Rep; Agrawal et al. 2022, Water Research; Calderon-Franco et al, 2022, Science of the Total Environment; Agrawal et al. 2021, BioRXiv], consistently achieves high horizontal genome coverage (see **Figure 1** below for an example). This high coverage is critical for accurately assessing the impact of reference bias on lineage abundance estimates. Our protocol has been regularly maintained and updated to ensure its relevance and applicability to current SARS-CoV-2 variant surveillance, and we are still running SARS-CoV-2 sequencing from wastewater samples routinely (see Figure 5 here: https://www.rki.de/EN/Content/Institute/DepartmentsUnits/InfDiseaseEpidem/Div32/WastewaterSurveillance/Report.html?__blob=publicationFile).

We know and acknowledge the potential benefits of a more diverse dataset that includes multiple sequencing technologies for a broader analysis of variability and robustness. However, such an investigation would greatly expand the scope and complexity of our study and take the focus away from the critical issue of reference bias. Indeed, future studies could benefit from a comparative analysis of different sequencing platforms to further elucidate the nuances of analyzing lineage composition in wastewater samples. We also discuss this potential limitation of our study now in the manuscript more prominently at the beginning of the “Potential Implications” section:

“In this study, we focus exclusively on Ion Torrent sequencing data to specifically investigate the influence of reference database composition and analysis parameters on lineage abundance estimates in wastewater sequencing. While acknowledging that incorporating data from additional platforms like PacBio, Nanopore, and Illumina could broaden the analysis of variability and robustness, we chose Ion Torrent due to its established efficacy in achieving high horizontal genome coverage in our sequencing runs \cite{agrawal2022genome,agrawal2022prevalence,agrawal2023comprehensive}, critical for assessing the impact of reference bias. This focused approach allows us to explore the considerable effects that reference selection and analytical settings have on lineage abundance results, a crucial area for accurate viral surveillance. Future studies might explore a comparative analysis across different platforms to enhance understanding of lineage composition and abundance estimation in wastewater samples. However, our current study is intentionally limited to specific research objectives related to reference bias in a \mutation{} and \kallisto{} setting and in the context of declining clinical sequencing and the dilution of available reference sequences.”

In summary, while we agree with the reviewer on the inherent value of incorporating diverse sequencing data, the specific aims of our study, coupled with the demonstrated efficacy of our optimized Ion Torrent sequencing protocol, justify our focused approach. Our results provide valuable insight into the distinct impact of reference database composition on lineage abundance estimation in wastewater sequencing and differences between a mutation-focused and sequence-focused approach, a topic of critical importance for future accurate viral pathogen surveillance and management — especially in the post-pandemic period.

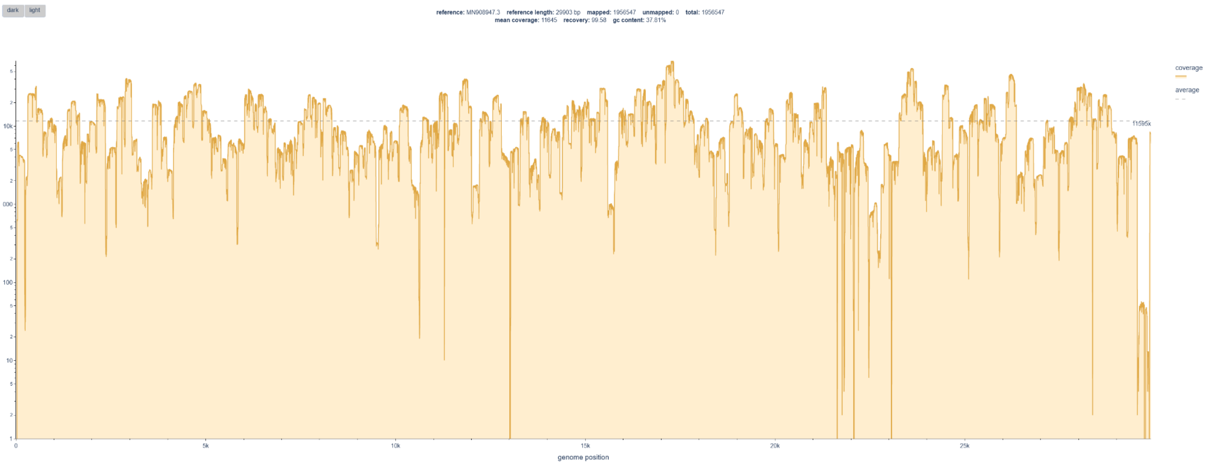

Fig. 1 Example genome coverage plot for a wastewater sample sequenced with the Ion Torrent protocol used in our study.

Q: 2. While the study showcases a variety of pipelines based on mutation-based and sequence-based tools in Table 1, the evaluation of three data sets was limited to only using MAMUSS (as a mutation-based reference) and VLQ-nf (as a sequence-based reference). For more conclusive guidance in pipeline selection, it is advisable for the authors to expand their analysis to include at least two or three more pipelines. This recommendation aligns with observations noted by the authors at line 619, suggesting a comprehensive benchmark comparison would significantly enhance the study's utility and appeal to readers seeking optimal pipeline strategies.

A: Thanks for the comment and the suggestion to extend our analysis to more pipelines to provide a more coherent study for selecting bioinformatics tools for lineage abundance estimation from wastewater sequencing data. Indeed, evaluating a broader range of tools/pipelines could greatly enrich our study by providing a more holistic view of the available methods and their relative performances under different conditions.

However, as outlined above, the focus of our study was to investigate the impact of reference bias when analyzing SARS-CoV-2 from wastewater sequencing data. The emphasis on reference bias was chosen because it is under-researched in the context of wastewater genomics and has a large impact on the accurate estimation of lineage abundance. The use of the MAMUSS (mutation-based) and VLQ-nf (sequence-based) pipelines in our analysis was determined by their relevance to the core objectives of the study and the specific hypothesis tested in relation to reference bias and comparing an exemplary mutation- and sequence-based approach. In addition, and as we wrote in the manuscript:

"A major benefit of implementing the representative methods [MAMUSS, VLQ-nf] was the complete control over code, parameters, and inputs, which allowed us to understand better, compare, and interpret the results of our benchmark study and the effects on the reference design."

The recommendation to perform a comprehensive benchmark comparison of additional pipelines is indeed valid and aligns with our recognition of their importance, as noted in line 658 of our manuscript. We agree with the reviewer that such an analysis would greatly enhance the utility of the study and provide valuable guidance to researchers in the field. However, the inclusion of comprehensive benchmarking would have been beyond the scope of this study, primarily due to the focus of our research question and the extensive resources that would be required for a rigorous and meaningful comparison of a wide range of bioinformatics tools.

However, recognizing that there is an urgent need for comprehensive benchmarks in this area, we are actively collaborating with international colleagues to fill this gap. Motivated by the outlook we're giving in the manuscript, we worked on a review of challenges and opportunities in wastewater genomic surveillance (https://arxiv.org/abs/2309.13326, submitted for peer review) and a rigorous comparison of different sequencing technologies, simulated data, and a variety of bioinformatics tools for lineage abundance estimation (in progress). This forthcoming work aims to provide the comprehensive comparison that both the reviewer and we believe is necessary to advance the field. We added a citation for the preprint of this ongoing work as evidence of our commitment to this important endeavor.

In summary, while our current study focuses on the specific problem of reference bias and showcases the difference between mutation- and sequence-based approaches to reconstruct this necessary reference, we recognize the reviewer's point. We are taking concrete steps to address the broader need for comprehensive benchmarking of bioinformatics pipelines in the context of wastewater genomic surveillance. We are confident that our upcoming review and benchmark study will significantly contribute to filling this gap and provide valuable insights to researchers seeking optimal pipeline strategies for SARS-CoV-2 analysis in wastewater samples.
