# Supplementary figures and images for: Impact of reference design on estimating SARS-CoV-2 lineage abundances from wastewater sequencing data
Source: Gigascience. 2024 Aug 8;13:giae051. doi: 10.1093/gigascience/giae051 (PMC11308188; doi:10.1093/gigascience/giae051)

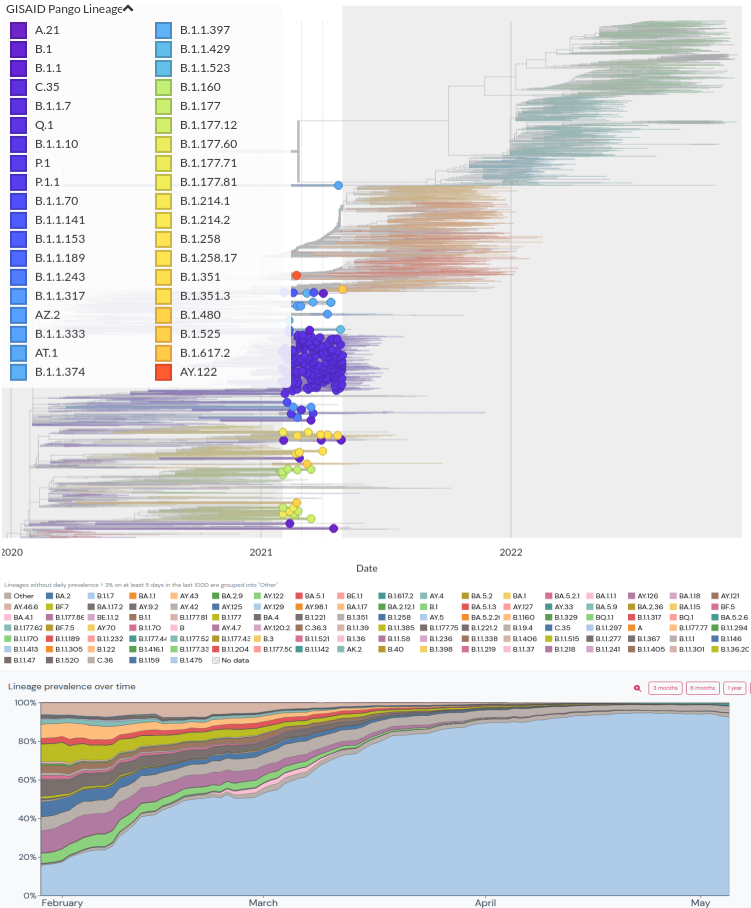

Supplement: giae051_Supplemental_Files [file giae051_supplemental_files.zip › FigureS1_Supplement.png]

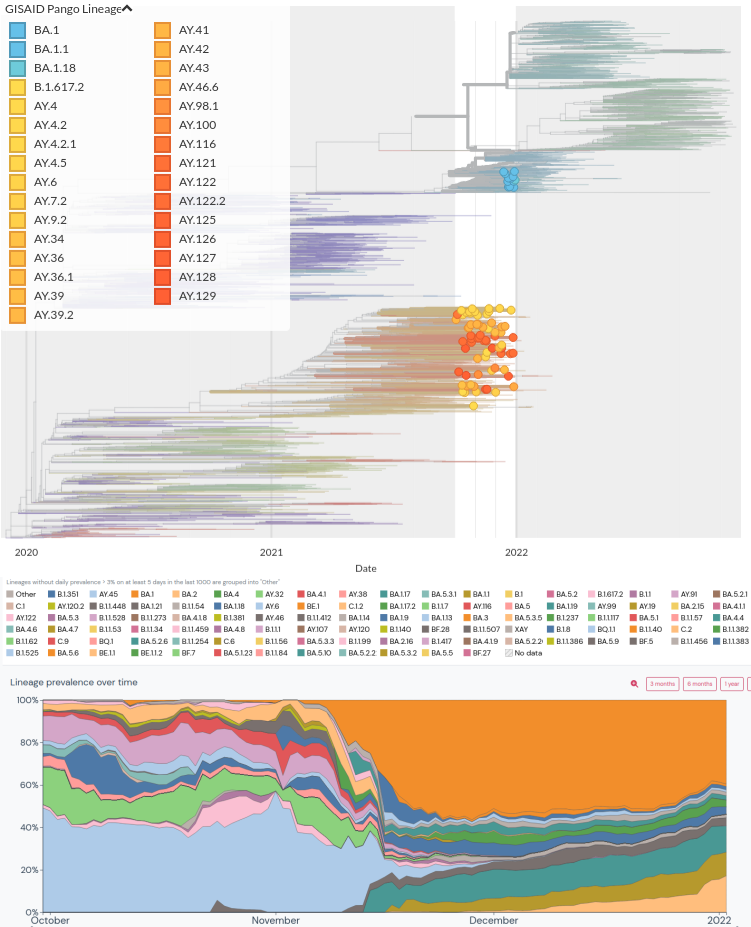

Supplement: giae051_Supplemental_Files [file giae051_supplemental_files.zip › FigureS2_Supplement.png]

PanEU-Ger AAF Experiment

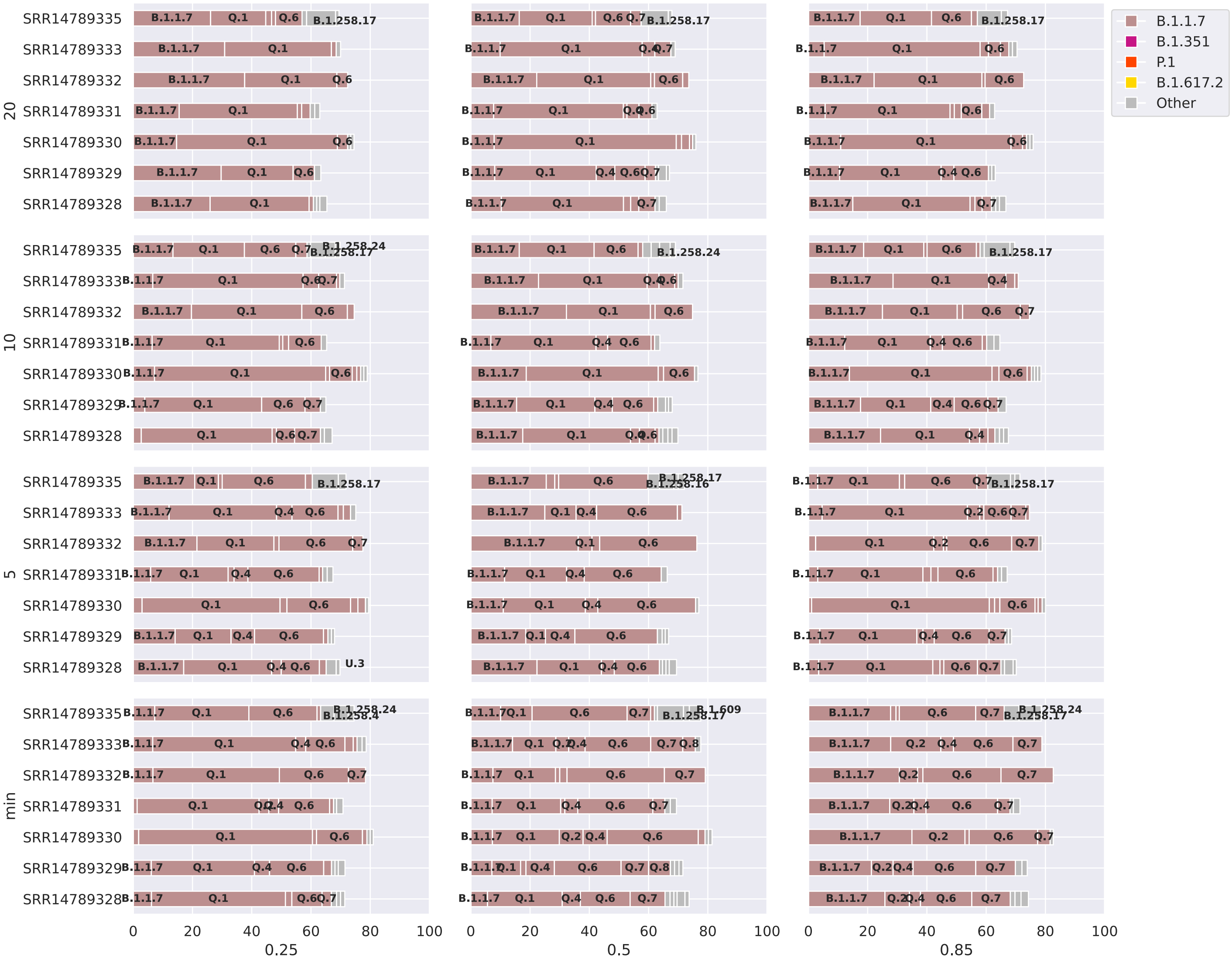

Supplement: giae051_Supplemental_Files [file giae051_supplemental_files.zip › FigureS3_Supplement.pdf]

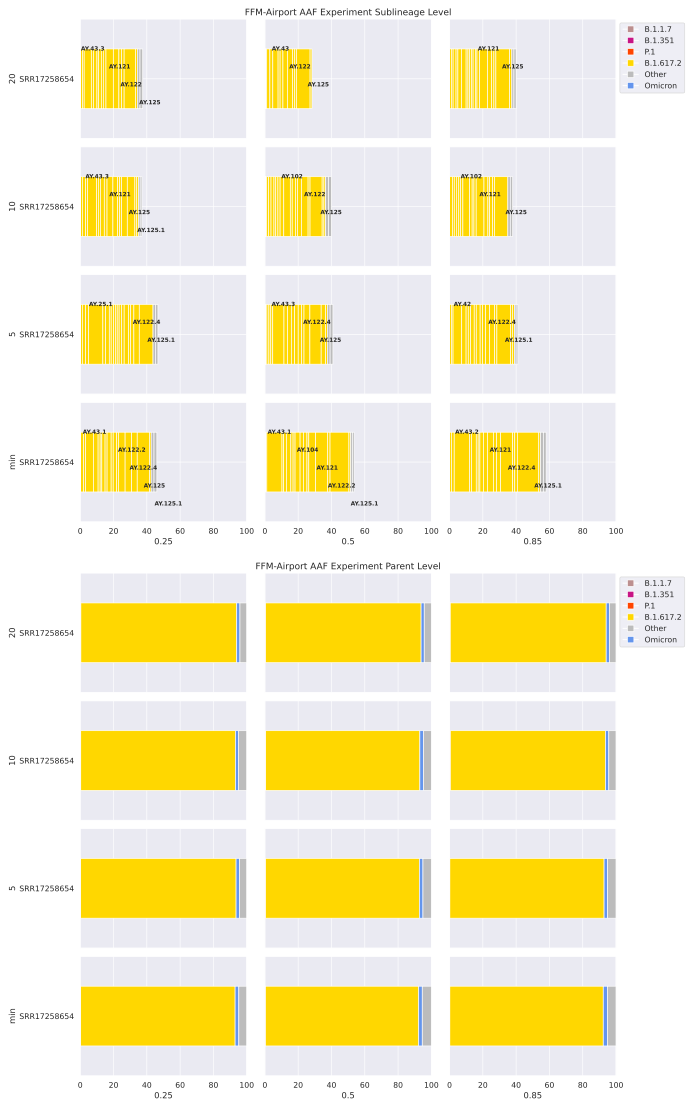

Supplement: giae051_Supplemental_Files [file giae051_supplemental_files.zip › FigureS4_Supplement.png]

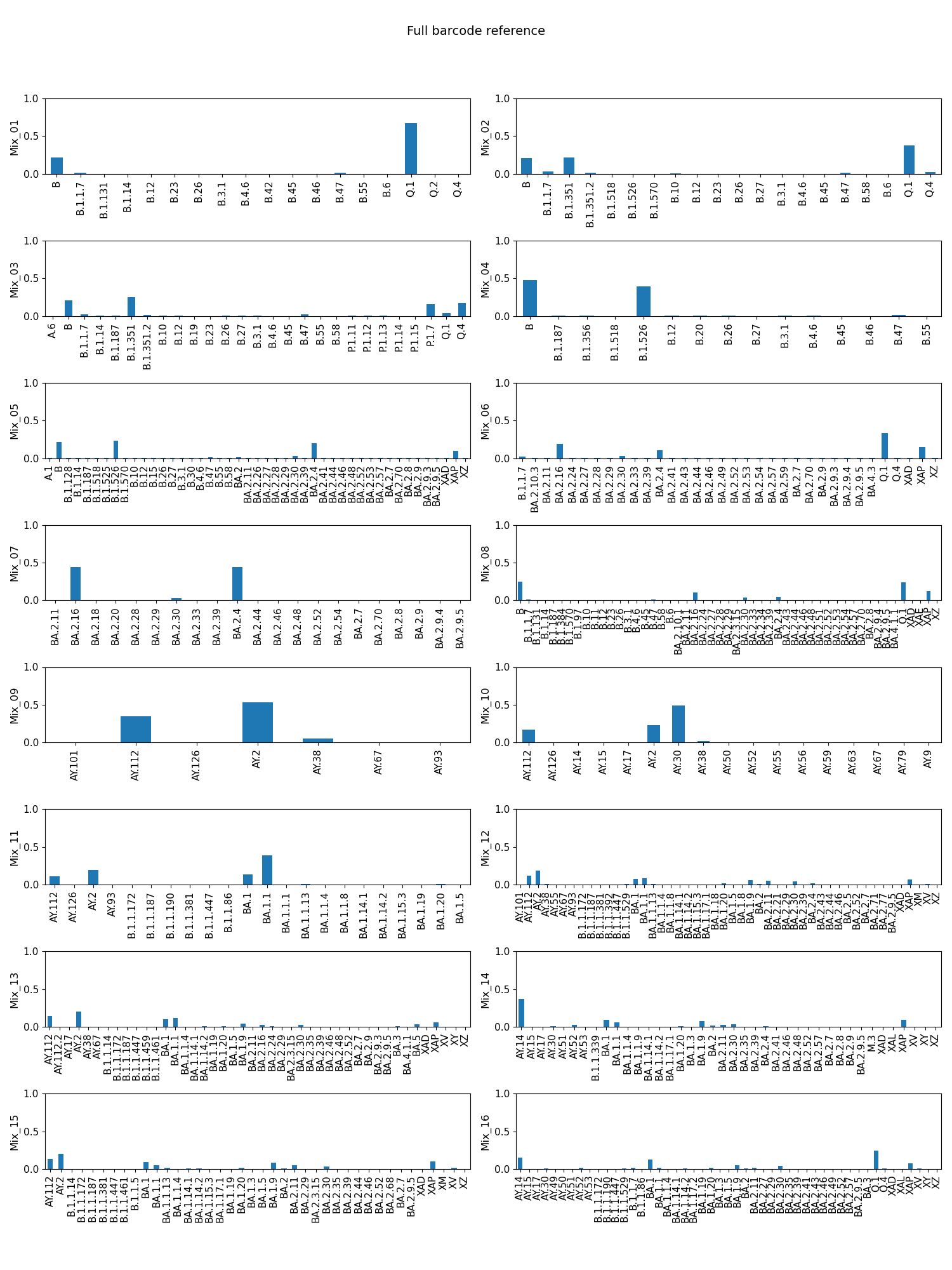

Supplement: giae051_Supplemental_Files [file giae051_supplemental_files.zip › FigureS5_Supplement.png]

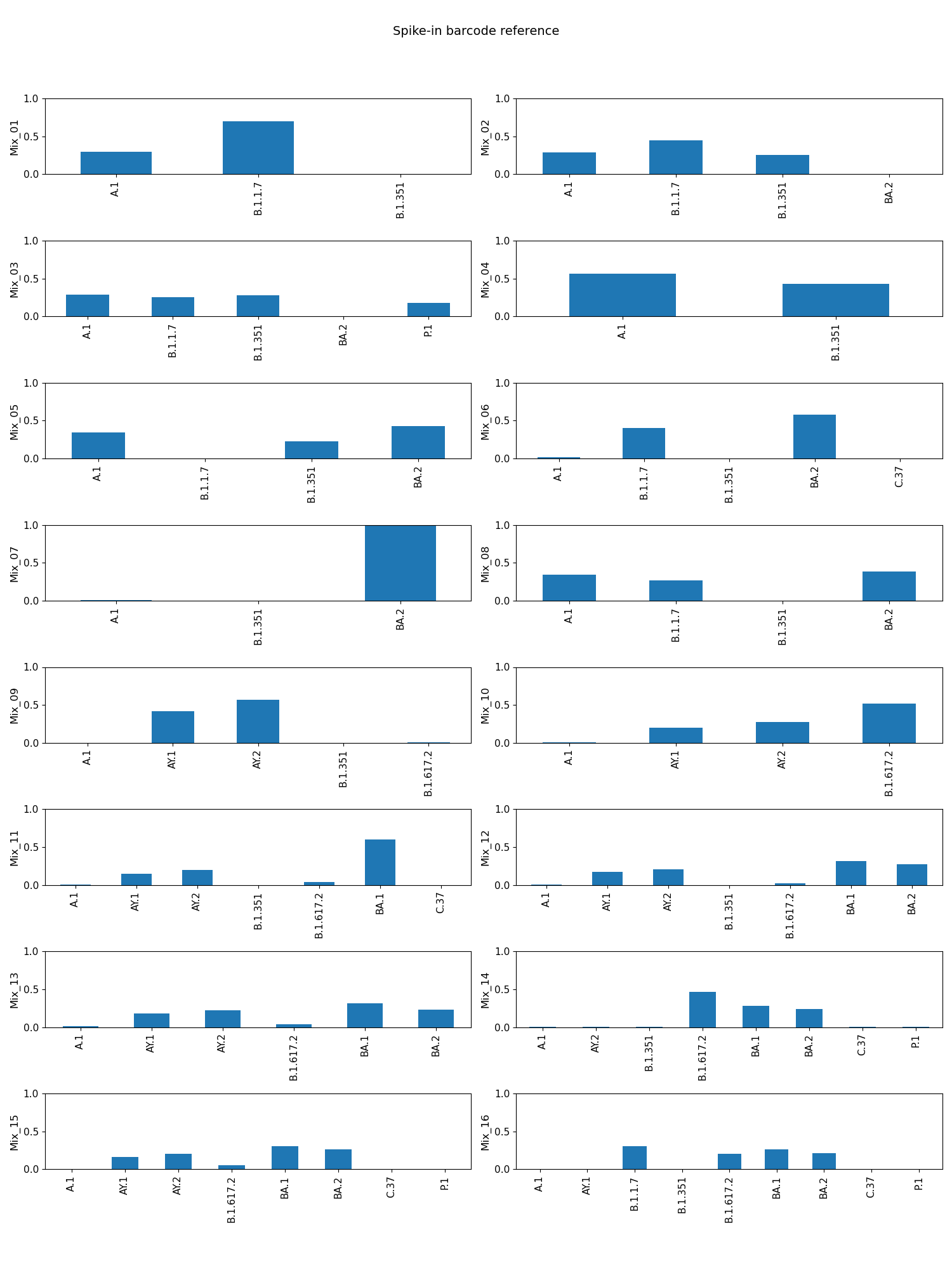

Supplement: giae051_Supplemental_Files [file giae051_supplemental_files.zip › FigureS6_Supplement.png]
