# Supplementary material for: Impact of reference design on estimating SARS-CoV-2 lineage abundances from wastewater sequencing data
Source: Gigascience. 2024 Aug 8;13:giae051. doi: 10.1093/gigascience/giae051 (PMC11308188; doi:10.1093/gigascience/giae051)
Supplement: giae051_Response_to_Reviewer_Comments_Original_Submission [file giae051_response_to_reviewer_comments_original_submission.pdf]

Author's Response To Reviewer Comments

Dear Dr. Zhou, Dear reviewers,

Thank you again for handling our manuscript titled “Impact of reference design on estimating SARS-CoV-2 lineage abundances from wastewater sequencing data" (GIGA-D-23-00161).

We appreciate the constructive comments of the two reviewers and are pleased to attach the revised version of the manuscript along with our detailed responses to the reviewers' comments. We attached our detailed response letter as an additional PDF. Please let us know if that did not work.

We look forward to the possibility of our study being published in GigaScience and believe it will make a valuable contribution to the ongoing efforts in understanding and utilizing wastewater sequencing data for public health surveillance.

Thank you for considering our revised manuscript. We are eager to see it contribute to the scientific community and help advance our understanding of SARS-CoV-2 dynamics in wastewater-based epidemiology.

Best,

Martin Hölzer
(on behalf of all co-authors)
