# Supplementary material for: Impact of reference design on estimating SARS-CoV-2 lineage abundances from wastewater sequencing data
Source: Gigascience. 2024 Aug 8;13:giae051. doi: 10.1093/gigascience/giae051 (PMC11308188; doi:10.1093/gigascience/giae051)
Supplement: giae051_Reviewer_1_Report_Original_Submission — Irene Bassano -- 9/20/2023 Reviewed [file giae051_reviewer_1_report_original_submission.pdf]

Reviewer Report

Title: Impact of reference design on estimating SARS-CoV-2 lineage abundances from wastewater sequencing data

Version: Original Submission **Date:** 9/20/2023

Reviewer name: Irene Bassano

Reviewer Comments to Author:

London, 19th September 2023
Dear all,
Please find attached my comments and suggestions to the manuscript.
In the manuscript "Impact of reference design on estimating SARS-CoV-2 lineage abundances from wastewater sequencing data" AÃŸmann et. al compare two methods, a sequence and mutation-based, respectively, to better understand the circulating lineages and sub-lineages in wastewater samples. Since the advent of wastewater-based epidemiology (WBE) as a tool to complement results from clinical data, there has been search for novel tools that can give robustness to the results and more importantly confidence in the data analysis. In this context, this manuscript is very important as it is contributing towards achieving that goal. This is clear in the fact that they have designed a new tool, namely MAMUSS.
1. One aspect however that the manuscript fails to mention is the difficulty in reconstructing full genome sequences from wastewater data. This has been one of the biggest problems since it is widely accepted that viral particles in water do degrade, and consequently what is being sequenced is a partial genome. Consensus sequences are therefore very difficult to obtain.
2. Another aspect that the authors fail to mention in the introduction or as a point of discussion, is how a variant is defined and how we take this information from clinical samples to then adopt it to define variants in environmental samples, although some relevant tools are mentioned such as COJAC and MMMVI. Yet, how these are used, it is not explained.
3. The manuscript is well written, there are some repetitive sentences that need to be removed (see comments on PDF) as well as a couple of sentences which are not grammatically correct (see comments on PDF).
4. It is worth mentioning that the words "variants" and "lineages" are used interchangeably. I do suggest they choose one term only.
5. The manuscript mentions several times the presence of false and true positive, however does not mention how these were calculated. These need to be supported by a small statistical test.
6. There are minor corrections throughout the manuscript that need to be address. All these are highlighted as comments in the original manuscript.
Please do not hesitate to get back in touch should you require further assistance.

Methods

Are the methods appropriate to the aims of the study, are they well described, and are necessary controls included? Choose an item.

Conclusions

Are the conclusions adequately supported by the data shown? Choose an item.

Reporting Standards

Does the manuscript adhere to the journal's guidelines on minimum standards of reporting? Choose an item.

Choose an item.

Statistics

Are you able to assess all statistics in the manuscript, including the appropriateness of statistical tests used? Choose an item.

Quality of Written English

Please indicate the quality of language in the manuscript: Choose an item.

Declaration of Competing Interests

Please complete a declaration of competing interests, considering the following questions:

- Have you in the past five years received reimbursements, fees, funding, or salary from an organisation that may in any way gain or lose financially from the publication of this manuscript, either now or in the future?
- Do you hold any stocks or shares in an organisation that may in any way gain or lose financially from the publication of this manuscript, either now or in the future?
- Do you hold or are you currently applying for any patents relating to the content of the manuscript?
- Have you received reimbursements, fees, funding, or salary from an organization that holds or has applied for patents relating to the content of the manuscript?
- Do you have any other financial competing interests?
- Do you have any non-financial competing interests in relation to this paper?

If you can answer no to all of the above, write 'I declare that I have no competing interests' below. If your reply is yes to any, please give details below.

I declare that I have no competing interests

I agree to the open peer review policy of the journal. I understand that my name will be included on my report to the authors and, if the manuscript is accepted for publication, my named report including any attachments I upload will be posted on the website along with the authors' responses. I agree for my report to be made available under an Open Access Creative Commons CC-BY license (http://creativecommons.org/licenses/by/4.0/). I understand that any comments which I do not wish to be included in my named report can be included as confidential comments to the editors, which will not be published.

Choose an item.

To further support our reviewers, we have joined with Publons, where you can gain additional credit to further highlight your hard work (see: https://publons.com/journal/530/gigascience). On publication of this paper, your review will be automatically added to Publons, you can then choose whether or not to claim your Publons credit. I understand this statement.

Yes Choose an item.
