# Supplementary material for: Impact of reference design on estimating SARS-CoV-2 lineage abundances from wastewater sequencing data
Source: Gigascience. 2024 Aug 8;13:giae051. doi: 10.1093/gigascience/giae051 (PMC11308188; doi:10.1093/gigascience/giae051)
Supplement: giae051_Reviewer_2_Report_Original_Submission — Liuyang Zhao -- 1/15/2024 Reviewed [file giae051_reviewer_2_report_original_submission.pdf]

Reviewer Report

Title: Impact of reference design on estimating SARS-CoV-2 lineage abundances from wastewater sequencing data

Version: Original Submission **Date:** 1/15/2024

Reviewer name: Liuyang Zhao

Reviewer Comments to Author:

In this study, the authors initiate a novel exploration by employing parameter escalation experiments to assess the impact of reference size and alternative allele frequency cutoffs on the effects of virus lineage composition in wastewater samples and their references. The research provides valuable insights into how different parameter settings influence outcomes in test data sets, particularly highlighting the role of virus lineage composition in wastewater samples and the corresponding references. Detailed parameters for these analyses are made available in several bash files at osf.io/upbqj. Despite these significant contributions, certain areas could benefit from further enhancement:

1.The current methodology utilizes Ion Torrent for testing mock samples. However, this approach may not fully capture the variability in alignment and sub-lineage analysis. Incorporating additional sequencing data from PacBio, Nanopore, and Illumina would offer a more comprehensive examination of these aspects, potentially leading to more robust findings.

2.While the study showcases a variety of pipelines based on mutation-based and sequence-based tools in Table 1, the evaluation of three data sets was limited to only using MAMUSS (as a mutation-based reference) and VLQ-nf (as a sequence-based reference). For more conclusive guidance in pipeline selection, it is advisable for the authors to expand their analysis to include at least two or three more pipelines. This recommendation aligns with observations noted by the authors at line 619, suggesting a comprehensive benchmark comparison would significantly enhance the study's utility and appeal to readers seeking optimal pipeline strategies.

Methods

Are the methods appropriate to the aims of the study, are they well described, and are necessary controls included? Choose an item.

Conclusions

Are the conclusions adequately supported by the data shown? Choose an item.

Reporting Standards

Does the manuscript adhere to the journal’s guidelines on minimum standards of reporting? Choose an item.

Choose an item.

Statistics

Are you able to assess all statistics in the manuscript, including the appropriateness of statistical tests used? Choose an item.

Quality of Written English

Please indicate the quality of language in the manuscript: Choose an item.

Declaration of Competing Interests

Please complete a declaration of competing interests, considering the following questions:

- Have you in the past five years received reimbursements, fees, funding, or salary from an organisation that may in any way gain or lose financially from the publication of this manuscript, either now or in the future?
- Do you hold any stocks or shares in an organisation that may in any way gain or lose financially from the publication of this manuscript, either now or in the future?
- Do you hold or are you currently applying for any patents relating to the content of the manuscript?
- Have you received reimbursements, fees, funding, or salary from an organization that holds or has applied for patents relating to the content of the manuscript?
- Do you have any other financial competing interests?
- Do you have any non-financial competing interests in relation to this paper?

If you can answer no to all of the above, write 'I declare that I have no competing interests' below. If your reply is yes to any, please give details below.

I declare that I have no competing interests.

I agree to the open peer review policy of the journal. I understand that my name will be included on my report to the authors and, if the manuscript is accepted for publication, my named report including any attachments I upload will be posted on the website along with the authors' responses. I agree for my report to be made available under an Open Access Creative Commons CC-BY license (http://creativecommons.org/licenses/by/4.0/). I understand that any comments which I do not wish to be included in my named report can be included as confidential comments to the editors, which will not be published.

Choose an item.

To further support our reviewers, we have joined with Publons, where you can gain additional credit to further highlight your hard work (see: https://publons.com/journal/530/gigascience). On publication of this paper, your review will be automatically added to Publons, you can then choose whether or not to claim your Publons credit. I understand this statement.

Yes Choose an item.
